# *Acinetobacter baumannii* Global Clone-Specific Resistomes Explored in Clinical Isolates Recovered from Egypt

**DOI:** 10.3390/antibiotics12071149

**Published:** 2023-07-04

**Authors:** Samira M. Hamed, Walid F. Elkhatib, Hanka Brangsch, Ahmed S. Gesraha, Shawky Moustafa, Dalia F. Khater, Mathias W. Pletz, Lisa D. Sprague, Heinrich Neubauer, Gamal Wareth

**Affiliations:** 1 Department of Microbiology and Immunology, Faculty of Pharmacy, October University for Modern Sciences and Arts (MSA), Giza 12451, Egypt; satwa@msa.edu.eg; 2 Microbiology and Immunology Department, Faculty of Pharmacy, Ain Shams University, African Union Organization Street, Cairo 11566, Egypt; 3 Department of Microbiology and Immunology, Faculty of Pharmacy, Galala University, Suez 43727, Egypt; 4 Institute of Bacterial Infections and Zoonoses, Friedrich-Loeffler Institut, 07743 Jena, Germany; hanka.brangsch@fli.de (H.B.); lisa.sprague@fli.de (L.D.S.); heinrich.neubauer@fli.de (H.N.); 5 Department of Pharmacology and Toxicology, Faculty of Pharmacy, Tanta University, Tanta 31511, Egypt; pg_171873@pharm.tanta.edu.eg; 6 Faculty of Veterinary Medicine, Benha University, Toukh 13736, Egypt; shawky.mustafa@fvtm.bu.edu.eg; 7 Tanta Laboratory, Animal Health Research Institute, Agricultural Research Center, Tanta 31511, Egypt; dr.daliakhater@gmail.com; 8 Institute of Infectious Diseases and Infection Control, Jena University Hospital, 07747 Jena, Germany; mathias.pletz@med.uni-jena.de

**Keywords:** *Acinetobacter baumannii*, WGS, global clones, resistance, resistome, Egypt, MDR, XDR, PDR

## Abstract

*Acinetobacter baumannii* (*A. baumannii*) is a highly problematic pathogen with an enormous capacity to acquire or upregulate antibiotic drug resistance determinants. The genomic epidemiology and resistome structure of 46 *A. baumannii* clinical isolates were studied using whole-genome sequencing. The isolates were chosen based on reduced susceptibility to at least three classes of antimicrobial compounds and were initially identified using MALDI-TOF/MS, followed by polymerase chain reaction amplification of *bla*_OXA-51-like_ genes. The susceptibility profiles were determined using a broth microdilution assay. Multi-, extensive-, and pan-drug resistance was shown by 34.8%, 63.0%, and 2.2% of the isolates, respectively. These were most susceptible to colistin (95.7%), amikacin, and trimethoprim/sulfamethoxazole (32.6% each), while only 26.1% of isolates were susceptible to tigecycline. In silico multi-locus sequence typing revealed 8 Pasteur and 22 Oxford sequence types (STs) including four novel STs (ST^Oxf^ 2805, 2806, 2807, and 2808). The majority of the isolates belonged to Global Clone (GC) 2 (76.4%), GC5 (19.6%), GC4 (6.5%), GC9 (4.3%), and GC7 (2.2%) lineages. An extensive resistome potentially conferring resistance to the majority of the tested antimicrobials was identified in silico. Of all known carbapenem resistance genes, *bla*_OXA-23_ was carried by most of the isolates (69.6%), followed by IS*Aba1*-amplified *bla*_ADC_ (56.5%), *bla*_NDM-1_ and *bla*_GES-11_ (21.7% each), and *bla*_GES-35_ (2.2%) genes. A significant correlation was found between carbapenem resistance and *carO* mutations, which were evident in 35 (76.0%) isolates. A lower proportion of carbapenem resistance was noted for strains possessing both *bla*_OXA-23_- and *bla*_GES-11_. Amikacin resistance was most probably mediated by *armA*, *aac(6′)-Ib9*, and *aph(3′)-VI*, most commonly coexisting in GC2 isolates. No mutations were found in *pmrABC* or *lpxACD* operons in the colistin-resistant isolates. Tigecycline resistance was associated with *adeS* (N268Y) and *baeS* (A436T) mutations. While the lineage-specific distribution of some genes (e.g., *bla*_ADC_ and *bla*_OXA-51-like_ alleles) was evident, some resistance genes, such as *bla*_OXA-23_ and *sul1*, were found in all GCs. The data generated here highlight the contribution of five GCs in *A. baumannii* infections in Egypt and enable the comprehensive analysis of GC-specific resistomes, thus revealing the dissemination of the carbapenem resistance gene *bla*_OXA-23_ in isolates encompassing all GCs.

## 1. Introduction

*Acinetobacter baumannii* (*A. baumannii*) is among the most notorious and worrisome nosocomial pathogens currently receiving much attention [1]. Together with its extraordinary propensity to accumulate resistance genes, its distinct capacity for surviving harsh hospital environments and the very high potential of biofilm formation greatly contribute to the burden of hospital-acquired *A. baumannii* infections [2,3,4]. While mostly affecting intensive-care patients, reports of *A. baumannii* infections in other healthcare facilities are frequent [3]. Community-acquired cases of *A. baumannii* infections in patients with pre-existing comorbidities have also been reported [5,6].

Resistance to last-line antimicrobial agents has been globally encountered in *A. baumannii* infections [7,8]. Numerous reports on pan-drug-resistant *A. baumannii*, linked to high mortality, have been published [7,9,10]. Moreover, carbapenem-resistant *A. baumannii* was ranked by the World Health Organization (WHO) as the foremost pathogen for which new antimicrobial agents are urgently required [11]. Egypt is one of the countries most affected by carbapenem- and multidrug-resistant *A. baumannii* infections [12,13,14,15,16]. The isolation of carbapenem-resistant *A. baumannii* from foreigners returning from Egypt has been reported previously [17]. Deciphering the resistance background of *A. baumannii* isolates from Egypt could, therefore, add to our knowledge about this pathogen.

One aspect of antimicrobial resistance in *A. baumannii* that has not been thoroughly studied is the extent of variation between global clones. A clonal population structure has been identified for *A. baumannii* over the years [18], with at least nine successful globally disseminated clonal lineages known so far. These included global clones (GCs) 1 to 9 that are mostly dominated by GC2 and, to a lesser extent, GC1 [18,19,20]. An inter-clone variation of the pathogenic potential of *A. baumannii* has been demonstrated previously [18,21,22]. Likewise, a variation might also exist in their resistomes. Initially, the global clones only carried genes conferring resistance to aminoglycosides, sulfonamides, and tetracyclines. Due to various genetic mechanisms, sub-lineages also resistant to other antimicrobials, including carbapenems, could emerge successfully [21]. GC1 and GC2 have long been known to comprise the majority of isolates with multi-, extensive-, and pan-drug resistance phenotypes. Due to the dynamic nature and plasticity of the *A. baumannii* genome, a growing number of studies have reported the emergence of antimicrobial resistance phenotypes in other GCs as well [1,20,23,24]. Together with their pathogenic and epidemic potential, the resistome differences between apparently identical strains might reshape our perception of the risk posed by individual GCs and the public health precautions required for each. This might be facilitated by coupling the genomic epidemiology of *A. baumannii* isolates recovered in different parts of the world to their resistome structures.

Here, we conducted a large-scale whole-genome sequencing (WGS)-based study to explore the resistome structure of *A. baumannii* recovered from patients admitted to hospitals in Cairo, Egypt. In addition, we explored the contribution of different GCs to *A. baumannii* infections in Egypt and investigated their specific resistomes.

## 2. Results

### 2.1. Bacterial Isolates and Their Antimicrobial Susceptibility Profiles

**Figure 1:**
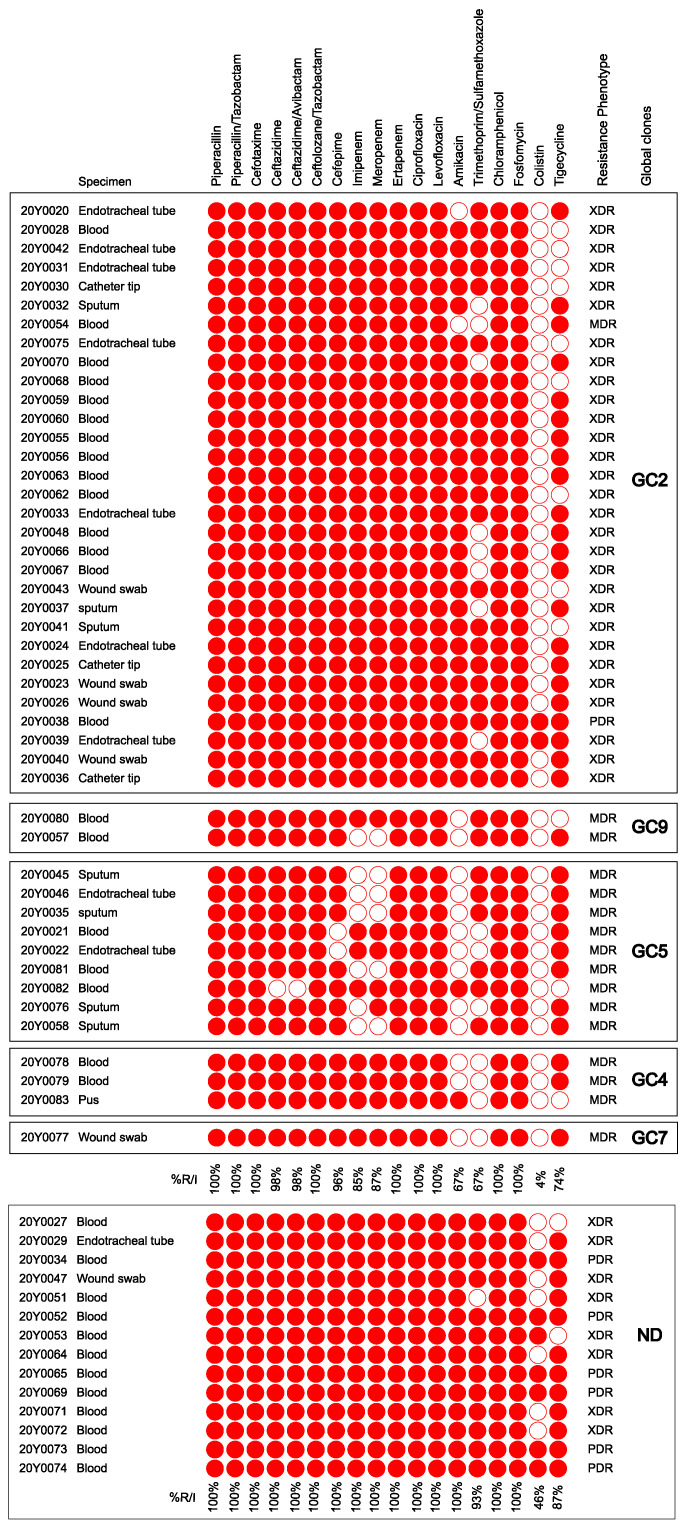
Clinical specimens and antimicrobial susceptibility profiles of all isolates. Reduced susceptibility (intermediate or resistance) is denoted by red icons, while the white ones correspond to full susceptibility. The isolates were segregated according to their global clones. MDR, multidrug-resistant; XDR, extensively drug-resistant; PDR, pan-drug resistant; GC, global clone; ND, the global clones could not be determined due to poor quality of draft genomes that were excluded from all subsequent genomic analyses; %R/I, percentage of reduced susceptibility to each antimicrobial agent. The figure was created using the MORPHEUS online tool (https://software.broadinstitute.org/morpheus, accessed on 3 January 2023) and edited using Inkscape version 1.2 (https://inkscape.org, accessed on 10 September 2022).

Based on the quality of the WGS results, i.e., purity of sample material, only 46 of the 60 original *A. baumannii* isolates were further analysed. The metadata of the isolates are given in Supplementary Materials Table S2. Of all tested antimicrobial agents, the isolates were most often susceptible to colistin (95.7%). A lower proportion of isolates showed susceptibility to amikacin and trimethoprim/sulfamethoxazole (32.6%), while only 12 (26.1%) isolates were susceptible to TG. Even though all isolates were resistant to ertapenem, 15.2% and 13.0% of them were susceptible to imipenem and meropenem, respectively. Full or intermediate resistance was exhibited by all isolates to penicillins and the cephalosporins cefotaxime and ceftolozane/tazobactam. Other cephalosporins (ceftazidime, ceftazidime/avibactam, and cefepime) were inactive against the majority (95.6–97.9%) of the isolates. Considering the full susceptibility profile of each isolate, 1 (2.2%), 16 (34.8%), and 29 (63.0%) isolates were classified as PDR, MDR, and XDR, respectively (Figure 1).

### 2.2. Draft Genomes and Annotation Features

Based on the short-read sequences, draft genomes consisting of 76 to 187 contigs were assembled with a mean N50 of 80,333 bp and covering 4approx. 81 to 86% of the reference genome (Table S3). The total length of the de novo assemblies varied between 3,798,591 bp and 4,247,432 bp with GC contents between 38.9% and 39.15%.

### 2.3. Phylogeny and Sequence Types

More than half of the isolates belonged to GC2 [25], encompassing four Pasteur STs and ten Oxford STs, as shown in Table S2. With less prevalence, one ST (ST602^Pas^/ST732^Oxf^) that belonged to GC5 [20] was also found in our collection, comprising 19.6% of the isolates. Three isolates (6.5%) belonged to GC4 [18] (ST15^Pas^/ST1115^Oxf^/). GC9 [19] was represented by two isolates that belonged to ST85^Pas^ and two Oxford STs (ST1580^Oxf^ and ST2808^Oxf^). Only one isolate was found to belong to GC7 [26] (ST113^Pas^/ST2246^Oxf^). Additionally, MLST analysis of the isolates revealed four novel Oxford allelic profiles in eight isolates, to which novel Oxford STs were assigned. The novel STs comprised ST2805^Oxf^ (GC2), ST2806^Oxf^ (GC2), ST2807^Oxf^ (GC2), and ST2808^Oxf^ (GC9). Minimum spanning trees depicting Pasteur and Oxford STs are identified here and their clonal complexes are shown in Figures S1 and S2, respectively.

**Figure 2:**
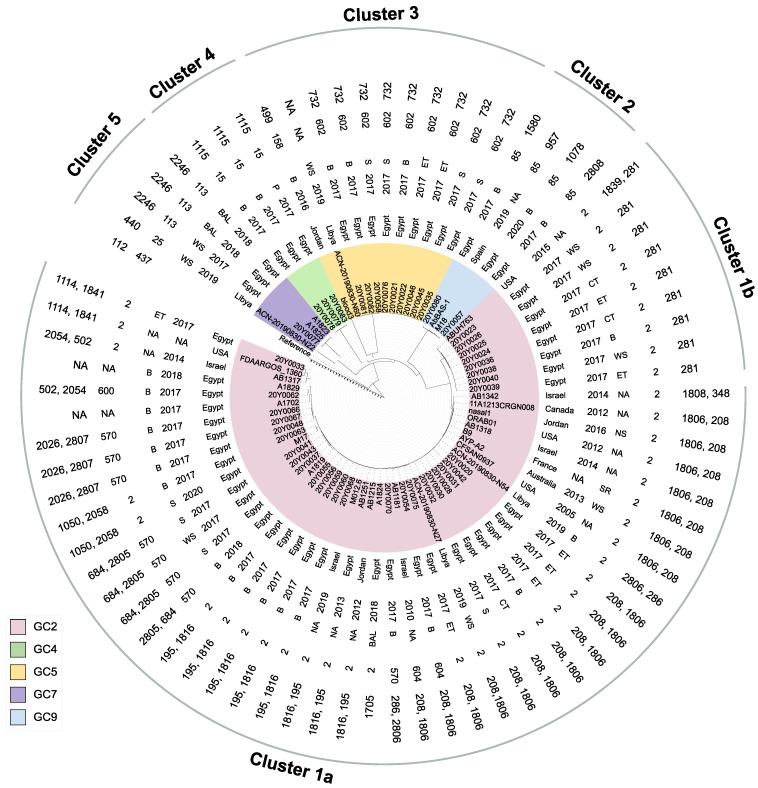
Maximum likelihood tree based on cgSNP alignment using *A. baumannii* ATCC 17978 (GCF_001593425.2) as the reference genome. Concentric labels refer to (from inside to outside): the country of isolation, year of isolation, specimen type, ST^(Pas)^, and ST^(Oxf)^. Clusters are coloured according to their global clones (GCs). B, blood; S, sputum; P, pus; WS, wound swab; ET, endotracheal tube; CT, catheter tip; BAL, bronchoalveolar lavage; and SR, Seine River. The tree was visualized using the interactive tree of life (iTOL) online tool v6.7 (https://itol.embl.de/, accessed on 12 March 2023) and edited using Inkscape version 1.2 (https://inkscape.org, accessed on 10 September 2022).

The cgSNP-based analysis encompassing the Egyptian isolates and global strains identified 64,994 SNPs. Five clusters that fully matched the GCs of the isolates were differentiated (Figure 2). GC2 isolates comprised the largest cluster (cluster 1), which was divided into two subclusters (1a and 1b). The larger one (cluster 1a) comprised all GC2 isolates except those belonging to ST281^Oxf^. Members of cluster 1a showed the highest genomic diversity of all tested isolates. Up to 4012 SNP differences were found among cluster 1a isolates, with some showing higher similarity to foreign strains. Isolates 20Y0037, 20Y0041, and 20Y0043 differed by only four SNPs from strain A1819 isolated in Egypt in 2020. As few as 13, 16, 20, 29, and 31 SNPs separated several isolates of cluster 1a from foreign strains collected in the USA, Libya, France, Jordan, and Israel. Six ST208/1806^Oxf^ strains differed by only 13 SNPs from the US-American strain CFSAN, which dated back to 2005. Cluster 1b included ST281^Oxf^ isolates. These showed high similarity with an SNP range of 0 to 4. In contrast, the distance to strain ABUH763, which was collected in the USA in 2015, was very high (minimum SNPs = 647). Within cluster 2 harbouring GC9 isolates, isolate 20Y0057 showed a closer SNP distance (*n* = 265) to strain AbBAS-1 from Spain than strain M18 (352 SNPs) from Egypt isolated in 2020. Moreover, isolate 20Y0057 differed from isolate 20Y0080 collected in the current study by 692 SNPs. Only five SNPs were found in strain ACN-20190830-N60 isolated in Libya in 2019 compared with GC5 isolates collected in the current study. These, in turn, showed SNP differences ranging from 0 to 7. GC4 and GC5 contained the fourth and fifth cluster, respectively. Even though isolate 20Y0077 showed a relatively close SNP distance (SNPs = 19–25) to other Egyptian strains, it exhibited 12,290 SNPs compared with a strain from Libya collected in 2019.

### 2.4. Correlation between Antimicrobial Resistance and Global Clones

Compared with the other investigated isolates, the isolates that belonged to GC2 showed higher resistance to amikacin (93.5% vs. 13.3%), colistin (6.5% vs. 0.0%), trimethoprim/sulfamethoxazole (74.2% vs. 53.3%), ceftazidime (100.0 vs. 93.3%), ceftazidime/avibactam (100.0 vs. 93.3%), imipenem (100.0% vs. 53.3%), and meropenem (100.0% vs. 60.0%). This was statistically significant only for amikacin, imipenem, and meropenem with *p*-values < 0.001. However, resistance to TG was lower in GC2 isolates than in the isolates belonging to other GCs (71.0% vs. 80.0%, *p*-value = 0.723). The XDR phenotype was exclusively found in GC2 isolates (*p*-value < 0.001).

### 2.5. Resistome Structure and Its Correlation to the Resistance Phenotypes

**Figure 3:**
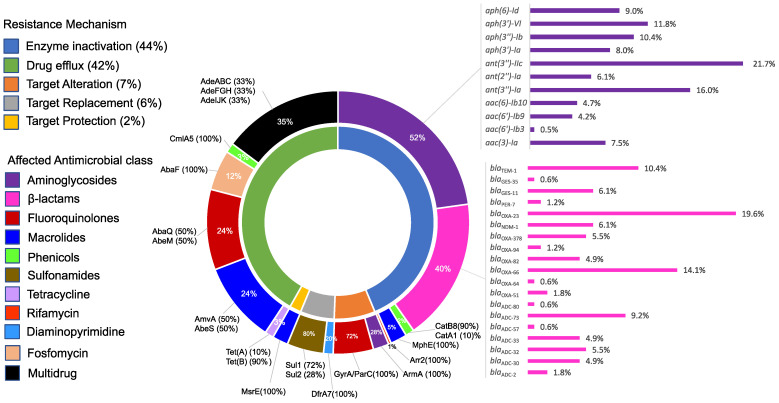
Graphical representation of the in silico identified potential resistance determinants of the total collection of *A. baumannii* isolates. The figure shows the contribution of different resistance mechanisms to the resistome and the antimicrobial classes affected by each. For each antimicrobial class, the contribution of individual resistance determinants is also shown. The figure was created using Microsoft Office Excel 2016 and edited using Inkscape version 1.2 (https://inkscape.org, accessed on 10 September 2022).

Scanning the draft genomes for antimicrobial resistance genes revealed a large resistome comprising at least 51 resistance determinants known to potentially confer resistance to ten antimicrobial classes through five resistance mechanisms, shown in Figure 3. Of these, antibiotic enzyme inactivation prevailed, followed by drug efflux, target alteration, target replacement, and finally target protection. Inactivating enzymes most frequently targeted aminoglycosides and β-lactams, including carbapenems. Detailed descriptions of the resistome and the potential contribution of different determinants to antimicrobial resistance exhibited by our collection are also shown in Figure 3.

A minimum of eleven resistance determinants were carried by all isolates. These included the efflux pump-coding genes *ade*ABC, *ade*FGH, *ade*IJK, *aba*F, *aba*Q, *amv*A, *abeM*, and *abe*S; the intrinsic aminoglycoside modifying enzyme-coding gene *ant(3″)-IIc* [27]; and, finally, six variants of the *bla*_OXA51-like_ genes and seven alleles of the *Acinetobacter*-derived cephalosporinase (ADC)-coding genes conferring intrinsic resistance to β-lactams [28,29].

**Figure 4:**
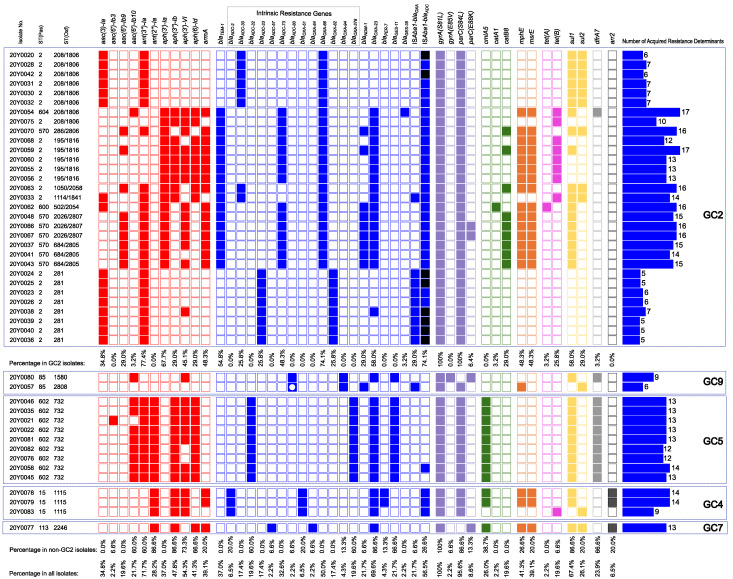
Distribution of resistance determinants potentially conferring resistance to different antimicrobial classes in all isolates. The isolates were arranged according to their phylogenetic relationships and were grouped into global clones. The presence or absence of resistance determinants is denoted by coloured or white squares, respectively. The white circle within the blue square denotes an interrupted *bla*_ADC_ gene. The figure is divided into panels of icons with different colours corresponding to antimicrobial classes affected by different resistance determinants. Red, aminoglycosides; blue, β-lactams; purple, fluoroquinolones; green, phenicoles; deep rose, tetracyclines; yellow, sulfonamides; grey, diaminopyrimidine; and dark grey, rifamycin. Undetermined IS*Aba1* upstream to *bla*_OXA_ or *bla*_ADC_ is denoted by black squares. The total numbers of acquired resistance genes carried by each isolate are represented by blue bars. The figure was created using the iTOL online tool v6.7 (https://itol.embl.de/, accessed on 12 March 2023) and edited using Inkscape version 1.2 (https://inkscape.org, accessed on 10 September 2022).

As inferred by Resfinder, the acquired genes conferring resistance to the last-line antimicrobials commonly used for treating MDR *A. baumannii* infections are detailed below. In addition, resistance to other antimicrobial classes was also acquired by our isolates, as shown in Figure 4. Most frequently, they targeted fluoroquinolones (100%) through mutations affecting the quinolone resistance-determining regions (QRDRs) of *gyrA* and *parC*. At least one sulfonamide/diaminopyrimidine resistance gene was carried by 71.7% of the isolates. Genes with the potential to confer resistance to chloramphenicol, macrolides, tetracyclines, and rifamycin were detected in 47.8%, 41.3%, 21.7%, and 6.5% of the isolates, respectively. Up to 17 acquired resistance determinants coexisted in our isolates.

#### 2.5.1. Carbapenem Resistance

In addition to the genes coding narrow (*bla*_TEM-1_) and extended-spectrum (*bla*_PER-7_) β-lactamases affecting penicillins and different generations of cephalosporins, carbapenem resistance determinants were also abundantly present in our collection. Of all known carbapenem resistance genes, *bla*_OXA-23_ prevailed (69.6%). Less frequently, the isolates carried IS*Aba1*-amplified *bla*_ADC_ (56.5%), *bla*_NDM-1_ and *bla*_GES-11_ (21.7% each), and finally *bla*_GES-35_ (2.2%). These were more likely to co-exist in combinations of two (50.0%) or three determinants (23.9%), as shown in Table 1. All except *bla*_GES-11_ were more frequently carried by carbapenem-resistant isolates compared with others. This was significant only for IS*Aba1*-preceded *bla*_ADC_ (Fisher’s Exact Test: *p*-value = 0.015 for imipenem and 0.036 for meropenem). The association between combinations of carbapenemase-coding genes and carbapenem resistance is shown in Table 1.

The contribution of the carbapenem susceptibility porin (CarO) to carbapenem resistance was also analysed. For this purpose, the nucleotide sequence of *carO* was extracted from all draft genomes. Compared with the wildtype gene sequence of *A. baumannii* ATCC 19606, multiple mutations were identified. The wildtype sequence of *carO* was fully retained by only 11 isolates comprising the GCs 5 and 9. Susceptibility to carbapenems was significantly more prevalent among the isolates carrying the wildtype genes (Fisher’s Exact Test, *p*-value < 0.001). None of the isolates carrying the mutated *carO* gene (35 isolates) were susceptible to any of the tested carbapenems. Multiple sequence alignment of the predicted amino acid structure of the protein product of the *carO* gene from all isolates compared with that of *A. baumannii* ATCC 19606 is shown in Figure S3.

#### 2.5.2. Amikacin Resistance

As shown in Figure 3, the aminoglycoside-modifying enzymes (AMEs) had the highest proportion among the genes encoding antimicrobial-inactivating enzymes found in the isolates. At least 11 acquired AME-coding genes were detected. Specifically, those affecting amikacin were thoroughly analysed. Only *aph(3′)-VI* and *aac(6′)-Ib* are known to affect amikacin. Corresponding genetic determinants were found in 54.3% and 43.5% of the isolates, respectively. An additional enzyme conferring broad-spectrum aminoglycoside resistance is the 16S rRNA methyltransferase enzyme coded by *armA*. The gene was identified in 39.1% of the isolates spanning three GCs (GC2, GC4, and GC7). Notably, the three genes coexisted exclusively in GC2 isolates. This corresponded to the significantly higher amikacin resistance recorded for GC2 isolates compared with others (93.5% versus 13.3%, *p*-value < 0.001). The combinations of amikacin resistance genes, their prevalence in different global clones, and their correlation to amikacin resistance are shown in Table 2.

Amikacin resistance was more common among the isolates carrying *armA* (77.8%) and *aph(3′)-VI* (56.0%) compared with others. Among the isolates with the potential for *aac(6′)-Ib* production, only those carrying *aac(6′)-Ib9* were non-susceptible to amikacin (Fisher’s Exact Test, *p*-value = 0.018). The association of *aph(3′)-VI* with an upstream IS*Aba125*, through which overexpression is known to be mediated [30], was also analysed. The analysis showed that *aph(3′)-VI* carried by all *bla*_NDM-1_-positive GC2 isolates was preceded by IS*Aba14* rather than IS*Aba125*. Interestingly, none of these were susceptible to amikacin.

#### 2.5.3. Colistin Resistance

Only two isolates (20Y0037 and 20Y0066) from our collection were non-susceptible to colistin. Both belonged to GC2, ST570^Pas,^ and a novel Oxford ST (684/2805^Oxf^ for 20Y0037 and 2026/2807^Oxf^ for 20Y0066). To explore the potential mechanisms underlying the colistin resistance in the two isolates, the *pmrCAB* and *lpxACD* operons were analysed. Using *A. baumannii* ATCC 19606 as reference, the gene sequences exhibited multiple missense mutations that resulted in amino acid alterations in *pmr*C (V58I—F166L—N300D—A370S—K531T), *pmr*B (A138T—N440H—A444V), *lpx*C (N287D), and *lpx*D (E117K). Similar analysis of other colistin-susceptible isolates with the same STs revealed the same mutation pattern, which was therefore considered a polymorphism. No unique genes were identified in the colistin-resistant isolates compared to other isolates with the same sequence type.

#### 2.5.4. Tigecycline Resistance

A total of 34 TG-resistant isolates was identified in our collection. Mutations previously linked to TG resistance [31,32,33,34] were carefully analysed. None of the TG-resistant isolates carried target site mutations in the ribosomal protein S10-coding gene (*rpsJ*). Mutations affecting the regulatory genes of the *ade*ABC efflux system, for which TG is a well-known substrate, were also analysed. These include *ade*RS and *bae*SR. Multiple mutations were found in the two regulatory systems carried by all strains when compared with *A. baumannii* ATCC 19606. Multiple sequence alignments of the predicted amino acid sequences of *ade*R, *ade*S, *bae*R, and *bae*S are shown in Figures S4–S7. Mutation hotspots of all genes and their correlation to TG resistance are also summarised in Table S4. Mutation patterns were found to be correlated with GCs rather than TG susceptibility profiles. With few exceptions, no mutations were found in TG-resistant isolates compared to TG-susceptible isolates of the same ST/GC. Nonetheless, one missense mutation in *adeS* resulting in the amino acid alteration N268Y was associated with TG resistance in all affected isolates. Similarly, threonine replaced alanine in position 436 of BaeS in the TG-resistant isolate 20Y0057 compared with the TG-susceptible isolate 20Y0080 which belonged to the same GC. While missense mutations were found in *adeRS* and *baeRS* carried by the TG-resistant isolate 20Y0077 (the solitary GC7 isolate) compared with *A. baumannii* ATCC19606, these were also found in closely related genomes retrieved from the BV-BRC database. While recently linked to TG resistance, *tet(A)* was carried by only one isolate in our collection that retained susceptibility to TG.

### 2.6. Global Clone-Specific Resistomes

As shown in Figure 4, the average number of acquired resistance determinants was highest (14.6) among a subset of GC2 isolates possessing both *bla*_OXA-23_ and *bla*_TEM-1_. Moreover, fewer determinants were carried by isolates of GC7 (13), GC5 (12.8), GC4 (12.3), and GC9 (7.5) than those belonging to GC2 that did not possess *bla*_OXA-23_ and *bla*_TEM-1_ (6).

**Figure 5:**
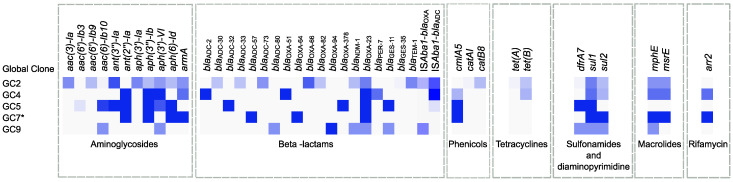
Heatmap showing the distribution of different resistance determinants in different GCs of Egyptian *A. baumannii* isolates. The intensity of the blue colour is directly proportional to the prevalence of each resistance gene in the isolates that belong to each GC. * Results shown for GC7 are based on one isolate only. The figure was created using the MORPHEUS online tool (https://software.broadinstitute.org/morpheus, accessed on 3 January 2023) and edited using Inkscape version 1.2 (https://inkscape.org, accessed on 10 September 2022).

In addition to the GC-specific mutations identified in the efflux pump regulatory genes described above, uneven distribution of some resistance determinants was also evident in different GCs (Figure 5). Some genes were unique for specific GCs, while others were common to some or all GCs. Additionally, some combinations of carbapenemase resistance determinants and amikacin resistance determinants were also unique for specific GCs, as shown in Table 1 and Table 2. Only two resistance genes were found to be present in all GCs (Figure 5). These included the carbapenemase-coding gene *bla*_OXA-23_ and the sulfonamide resistance gene *sul1*.

To sketch a preliminary image for the GC-specific resistomes identified in our isolates, we categorised the resistance genes into three groups: unique genes (found exclusively in one GC), conserved genes (found in all isolates of one or more GCs), and finally genes that were present but not fully conserved in one or more GCs (Table 3). Note that GC7 was not included in this analysis, as it was represented by a single isolate.

## 3. Discussion

The present study explored the genomic epidemiology of MDR, XDR, and PDR *A. baumannii* isolates recovered from tertiary care hospitals in Egypt. The resistome of the isolates and the global clone-specific resistomes were explored in vitro and using WGS. The phenotypic analysis of the isolates showed that the majority were XDR (63.0%), while 34.8% were MDR. Only one isolate showed a PDR phenotype. MDR and XDR *A. baumannii* have been abundantly isolated from Egyptian patients in previous studies [12,15,16,35,36]. A study from Egypt reported the isolation of PDR *A. baumannii* from a patient with ventilator-associated pneumonia; however, the authors did not test the susceptibility of the isolate to tigecycline [37]. In our collection, the highest susceptibility was shown for colistin, followed by amikacin and trimethoprim/sulfamethoxazole. Colistin resistance in carbapenem-resistant *A. baumannii* was first reported in Egypt by Abdulzahra et al. in 2018 [38]. While a high prevalence of colistin resistance was reported by Fam et al. [39], comparatively low levels of resistance were found among the present collection of isolates and by other studies from Egypt [12,13,15]. Similar susceptibility levels were shown by our isolates to both amikacin and trimethoprim/sulfamethoxazole. Moreover, trimethoprim/sulfamethoxazole showed superior activity against GC2 isolates compared with amikacin. The overall resistance to amikacin found here was comparable to the regional levels [13,15]. The relatively high susceptibility to trimethoprim/sulfamethoxazole supports the recent calls for reviving old antimicrobials such as trimethoprim/sulfamethoxazole, alone or in combination with colistin, for treatment of carbapenem-resistant *A. baumannii* infections [40,41]. Contrary to our findings, a higher level of resistance to trimethoprim/sulfamethoxazole was reported by other studies from Egypt [12,15].

The resistance of our isolates to penicillins and cephalosporins ranged from 96 to 100%. Due to their intrinsic class C and D β-lactamases, the majority of *A. baumannii* strains isolated locally [13,15] or globally [42,43] are non-susceptible to penicillins and cephalosporins. Of these, the highest activity against our isolates was shown by cefepime, the least affected by class C β-lactamases [44]. Up to 87% of the isolates were non-susceptible to imipenem/meropenem. This is comparable to the results of other studies from Egypt [13,45,46]. Meanwhile, all of the isolates were non-susceptible to ertapenem, which is known for its limited activity against glucose-non-fermenting Gram-negative bacteria including *A. baumannii* [47,48,49]. No activity was found for the tested fluoroquinolones against any of our isolates. Similar findings have also been reported by others [12,45]. In contrast to previous findings [12,15,50], a high number of TG-resistant isolates were found in our collection. Fosfomycin and chloramphenicol are not listed among the antimicrobial agents recommended by the CLSI for routine susceptibility testing in *A. baumannii* infections. Nevertheless, some studies have demonstrated their synergistic activity with colistin against *A. baumannii* [51,52,53]. The susceptibility of our isolates to both agents was investigated to assess potential effectiveness in treating MDR *A. baumannii* infections. However, all isolates were non-susceptible to both agents.

All STs assigned to the isolates included in our study were found to belong to high-risk GCs (GC2, GC4, GC5, GC7, and GC9), most of which have been previously reported in Egypt by other studies [12,15,39,54]. Although GC1 predominated in one study [12], none of our isolates belonged to this GC. However, GC2 isolates prevailed in our collection, as observed in previous studies from Egypt [15,39,54,55]. The prevalence of GC2 isolates among multidrug-resistant *A. baumannii* has been also reported globally [20,56,57,58]. A considerable share of our isolates had the ST602^Pas^/732^Oxf^ and, as determined by Al-Hassan et al. [20], belonged to GC5. Notably, this ST was first identified in *A. baumannii* isolates originating from Egypt [46] and was later reported from other countries such as Libya [59], Tunisia [60], Jordan [61], Germany [62], and Sudan [20]. Moreover, the cgSNP-based analysis of the isolates revealed a close evolutionary relationship to international strains, reflecting the great potential of the STs identified here for spreading across continents.

An extensive resistome comprising almost all known resistance mechanisms affecting several classes of antimicrobials was identified in silico in our collection. Unexpectedly, no plasmid-mediated quinolone resistance genes were identified [63,64]. However, all isolates carried mutations in the QRDRs of *gyrA* and *parC* genes. A highly conserved mutation pattern was observed in the isolates with only a few exceptions. While the *gyrA* mutation associated with S81L amino acid alteration was fully conserved in all isolates, some variability was found in *parC* mutations. The majority (91.3%) of isolates displayed the mutation pattern *gyrA*^S81L^/*parC*^S84L^. This mutation pattern has been linked to high-level fluoroquinolone resistance in *A. baumannii* [63,65,66]. However, some isolates showed different mutation patterns. In addition to the *gyrA*^S81L^ mutation, the isolates 20Y0066 and 20Y0067 carried two *parC* mutations, resulting in the amino acid alterations S84L and E88K. In agreement with [67], a single mutation in codon 88 of *parC* (E88K) in association with the *gyrA*^S81L^ mutation could be the reason for fluoroquinolone resistance in isolate 20Y0077. Isolate 20Y0080 carried a single *parC* mutation (E88K) and two mutations in *gyrA* (S81L and E85V). Mutations affecting codon 85 of *gyrA* have been observed in ciprofloxacin-resistant *A. baumannii* [67]. In addition to target site alterations, fluoroquinolones are extruded by a variety of efflux pumps whose coding genes were carried by all of our isolates. These included *adeABC*, *adeIJK*, *adeFGH*, *abeM*, and *abeS*.

Resistance to chloramphenicol shown by all of our isolates was conferred by the efflux pumps *ade*ABC, *ade*IJK, *ade*FGH, and *abe*M [68], whose coding genes were carried by all isolates. Only a small fraction of the isolates carried the chloramphenicol acetyltransferase-coding genes *catA4* and *catB5* and the major facilitator superfamily (MFS) efflux pump-coding gene *cmlA5*. Likewise, fosfomycin resistance was probably conferred by the major facilitator superfamily (MFS) transporter AbaF. Target site alteration was the major resistance mechanism for the antifolate combination of trimethoprim and sulfamethoxazole. Here, we identified *dfrA7*, which encoded a trimethoprim-resistant dihydrofolate reductase in 23.9% of the isolates, predominantly in GC5. Additionally, the sulfonamide-resistant dihydropteroate synthase-encoding genes *sul1* and *sul2* were carried by 67.4% and 26.1% of the isolates, respectively. Notably, they were distributed in all GCs.

Carbapenems have been the most appropriate treatment option for *A. baumannii* infections until the significant rise of carbapenem resistance throughout the world [69,70,71]. The *bla*_OXA-23_, the first known oxacillinase with carbapenem hydrolysing capacity [72] and the major contributor to carbapenem resistance in *A. baumannii* [73], was the most common (69.7%) carbapenem resistance determinant identified in our collection across all GCs. In line with this finding, a high prevalence has been reported from Egypt by other authors [74,75,76]. Most commonly, *bla*_OXA-23_ was combined with other potential carbapenem resistance genes. IS*Aba1-*amplified intrinsic *bla*_OXA_- and *bla*_ADC_-type β-lactamase-coding genes affecting carbapenems [77] were also found with a high prevalence. While IS*Aba1*-preceded-*bla*_OXA-51-like_ genes were confined to GC2 and GC9, IS*Aba1*-*bla*_ADC_ was found in GC2, GC4, and GC5. IS*Aba1*-amplified *bla*_OXA-51-like_ genes likely compensated for the lack of the *bla*_OXA-23_ gene in ST281^Oxf^/*bla*_OXA-82_ carbapenem-resistant isolates in which they were identified as the sole known carbapenem resistance mechanism. This has also been reported by Zander et al. [78].

Metallo-β-lactamases have been known to possess a 100–1000 fold higher carbapenemase activity than OXA-type carbapenemases [18]. Of these, *bla*_NDM-1_ predominated in most studies conducted on *A. baumannii* [15,56,74,76], and in our collection, too. Here, the gene *bla*_NDM-1_ was exclusively carried by some GC2 and GC9 isolates. The *bla*_NDM-1_ production was previously linked to ST85 (GC9) [18], which was thought to be a reservoir for *bla*_NDM-1_ in the Middle East [16,20,79]. With the same prevalence as *bla*_NDM-1_, the potential for Guyana extended spectrum β-lactamase variant 11 (GES-11) production was also evident in our collection. However, the weak potential of GES-11 for the hydrolysis of carbapenems was previously documented by [80]. The authors reported an imipenem MIC of 1 µg/mL for the *bla*_GES-11_ *A. baumannii* transconjugant compared with MIC of >24 µg/mL for the transconjugant carrying *bla*_OXA-23_. A lower imipenem resistance was noted in our collection among the group of isolates in which *bla*_OXA-23_ coexisted with *bla*_GES-11_ compared with those carrying *bla*_OXA-23_ with or without other carbapenem resistance genes. Despite the low sequence homology between the OXA-23 and GES-11, the two enzymes were previously reported to be anchored in the periplasmic space of *A. baumannii* AB5075 by interacting with the same lysine sites of the periplasmic domain of the outer membrane protein A (OmpA) [81]. The impact of this competitive binding to OmpA on the carbapenemase activity of OXA-23 might be of importance. Further studies are required to support this finding. Notably, *bla*_GES-11_ was confined to GC5 isolates and one GC9 isolate, and the allele *bla*_GES-35_ coexisted with *bla*_OXA-23_ in one carbapenem-resistant isolate. GES production has been observed in *A. baumannii* isolates from Egypt [13,76,82]. While previously linked to carbapenem resistance in *A. baumannii* isolates from Egypt [74,75,82], genes for *Klebsiella pneumoniae* carbapenemase (KPC) were not found in any of our isolates.

The influx of imipenem in *A. baumannii* has been known to be partly mediated by the outer membrane channel protein CarO [83]. Hence, amino acid alterations in CarO have been implicated in imipenem resistance. While overlooked by most studies, mutations affecting the CarO protein and their association with carbapenem resistance were investigated in the current study. A significant association was found between carbapenem resistance and *carO* mutations, corroborating previous studies [84,85].

Amikacin is one of the few antimicrobials for which susceptibility was retained by a substantial fraction of *A. baumannii* strains, isolated locally [15] and globally [86]. Among other aminoglycosides, resistance to amikacin is known to be imparted by *armA*, carried by 39.1% of the isolates. Additionally, numerous genes coding for aminoglycoside-modifying enzymes with the potential to inactivate different aminoglycosides, including amikacin (Table 2), were identified in our collection. Most of the isolates carrying either *armA*, *aph(3′)-VI*, or *aac(6′)-Ib9* were non-susceptible to amikacin. This was statistically significant only for *aac(6′)-Ib9*. However, 13/15 isolates without the mentioned genes were resistant to amikacin, highlighting the significant contribution of the efflux pumps in aminoglycosides resistance and the contribution of other resistance mechanisms that were hitherto unknown. High-level amikacin resistance due to *aph(3′)-VI* has been previously linked to IS*Aba125*-mediated overexpression of *aph(3′)-VI* (*aphA6*) [30,87]. As reported by others [88,89], we found that the gene was preceded by IS*Aba14* rather than IS*Aba125* in some isolates that were all non-susceptible to amikacin. This suggests that IS*Aba14* may have the same impact on *aph(3′)-VI* expression as IS*Aba125*. The synergistic effect of the amikacin resistance mechanism was also indicated by the significantly higher prevalence of amikacin resistance in GC2 isolates that carried combinations of *armA*, *aph(3′)-VI*, and *aac(6′)-Ib.*

Colistin and TG are currently the last resort for the treatment of multidrug resistant *A. baumannii* infections [90]. Colistin resistance has been linked to either the complete loss or modification of the outer membrane lipopolysaccharides, as well as to the presence of the mobilised colistin resistance (*mcr*) gene. The former impairs the membrane’s integrity, thereby potentiating susceptibility to other antimicrobial agents. This is mediated by mutations affecting lipid A biosynthesis genes (*lpxACD*) [91]. More commonly, colistin resistance arises due to the modification of bacterial lipopolysaccharides resulting from *pmrCAB* mutations [92]. Only two isolates in our collection of novel Oxford sequence types were non-susceptible to colistin. While missense mutations were found in the genes *pmrB*, *pmrC*, *lpxC*, and *lpxD* carried by the two strains compared with the corresponding genes of *A. baumannii* ATCC 19606, they were also found in colistin-susceptible isolates that belonged to the same STs. The altered expression of *pmrC* under the control of unexplored regulatory systems or another unknown colistin resistance mechanism might be the reason.

Although TG was specifically derived from tetracycline to evade tetracycline resistance both in Gram-negative and Gram-positive bacteria [93], sporadic cases of TG resistance have been reported recently. TG is not a substrate of the efflux pump *tet*(B), whose coding genes were identified in 19.6% of the isolates [94]. Nevertheless, a big fraction (74%) of our isolates were non-susceptible to TG. This far exceeds the numbers reported by other studies in the North African region [12,50]. While little is known about the mechanisms underlying TG resistance, few primarily chromosomal mechanisms have been confirmed for TG resistance in *A. baumannii*. These include target site alteration by mutations affecting the ribosomal protein S10 (*rpsJ*) [31,32] and overactive resistance nodulation division (RND) efflux pumps, particularly AdeABC [33], which, in turn, is controlled by the two-component systems *ade*SR [95] and BaeSR [34]. The first enzymatic resistance mechanism to TG was reported by [96], who described the TG-inactivating enzyme flavin-dependent monooxygenase *tet*(X). Plasmid-encoded *tet*(X), which confers high-level TG resistance, has been described previously [97,98]. Of the aforementioned mechanisms, only mutations affecting *adeS* (N268Y) and *baeS* (A436T) were exclusively found in TG-resistant isolates compared with TG-sensitive isolates of the same ST. Mutations affecting AdeABC regulatory systems were also reported as the sole TG-resistance mechanisms in *A. baumannii* in other studies [99]. While the amino acid alteration N268H was reported before as polymorphism in GC2 isolates [100], the impact of N268Y on the expression level of AdeABC and, hence, TG resistance has yet to be investigated. A recent study by Sawant et al. [101] reported the same prevalence of TG-resistant isolates as in our collection. The authors additionally demonstrated the overexpression of the *ade*ABC efflux pump associated with *adeRS* mutations including N268H. In addition to the previously mentioned TG resistance mechanisms, a recent study by Foong et al. [102] concluded that the MFS efflux pump *tet*A is up-regulated upon exposure to TG. The single-component pump then expels TG to the periplasm, from where it is then extruded by the tripartite RND efflux pumps *ade*ABC and *ade*IJK. The gene *tet*(A) was carried by only one isolate in our collection. However, the isolate was, surprisingly, susceptible to TG.

Of all our isolates, a subset of GC2 co-harbouring *bla*_TEM-1_ and *bla*_OXA-23_ can be considered to be of the highest public health concern. They carried the highest number of acquired resistance genes and exhibited resistance to the majority of antimicrobial classes. Moreover, they comprised 54.8% of GC2 and 36.9% of all isolates, which reflects their epidemic potential. In addition, GC5, GC4, and finally GC9 should also be closely monitored, due to their prevalence and the number of resistance genes. Although isolate 20Y0077 allocated to GC7 carried 13 acquired resistance genes, no reliable conclusion could be drawn about the threat imparted by GC7 as it was only represented by a single isolate. Additionally, the GC7-specific resistome could not be deduced from a single isolate. Further studies including a larger number of isolates belonging to all GCs are still required.

We also attempted to provide helpful markers for specific clones that might be utilised for inferring the burden of GCs in healthcare facilities with limited resources and where the seven locus-based MLST is not feasible. Such markers are based on antimicrobial resistance genes that might be routinely screened in microbiology laboratories. For this purpose, we categorised the antimicrobial resistance genes into three categories: unique, fully conserved, and not fully conserved. Of these, the unique genes that are fully conserved are best utilised for roughly assigning GCs. Concerning Table 3, unique genes mostly included *bla*_ADC_ and *bla*_OXA-51-like_ gene alleles. Similar findings were also reported by others [1,12,15,24,55,103]. Expanding our analysis to include the results of other studies revealed more intra-clonal conservation for *bla*_OXA-51_ compared with *bla*_ADC_ alleles [57,103]. While single-locus sequence typing might be required for defining specific *bla*_ADC_ and *bla*_OXA-51-like_ alleles, PCR detection of combinations of resistance genes fully conserved in each GC might provide an alternative epidemiologic tool within every laboratory’s reach.

## 4. Materials and Methods

### 4.1. Bacterial Isolates

A collection of nonreplicated 60 *A. baumannii* isolates recovered from various clinical specimens and showing non-susceptibility to at least three classes of antimicrobial agents were selected for WGS. All isolates were obtained from the strain collection at the microbiology laboratory of the Faculty of Pharmacy, October University for Modern Sciences and Arts; they were originally recovered from patients admitted to tertiary care hospitals in Cairo, Egypt, in 2017 as part of routine clinical care. The specimens from which the isolates were recovered included blood (33; 55.0%), endotracheal tubes (10; 16.7%), sputum (7; 11.7%), wound swabs (6; 10.0%), catheter tips (3; 5.0%), and pus (1; 1.7%). All isolates were preliminarily identified using conventional microbiological techniques [104]. The identity of the isolates was subsequently confirmed using Matrix-Assisted Laser Desorption/Ionisation Time-of-Flight Mass Spectrometry (MALDI-TOF MS) and PCR detection of intrinsic *bla*_OXA-51-like_ carbapenemase, *bla*_OXA-23-like_, *bla*_OXA-24-like_, *bla*_OXA-58-like_, and IS*Aba*1 genes as previously described [105].

### 4.2. Antimicrobial Susceptibility Testing

The initial screening for antimicrobial susceptibility was done using the Kirby–Bauer disk diffusion test performed and interpreted according to the Clinical and Laboratory Standards Institute (CLSI) criteria [106]. This was later confirmed by determining the minimum inhibitory concentrations (MICs) with broth microdilution assay in Mueller Hinton II broth using an automated MICRONAUT-S system (MICRONAUT, MERLIN Diagnostics GmbH, Bornheim-Hersel Germany). Micronaut-S-MDR-MRGN-Screening 3 and Micronaut-S β-Lactamases MIC plates were used for determining the MIC values of a large panel of antimicrobial agents according to the manufacturer’s instructions [106]. The tested panel of antimicrobial agents included: penicillins (piperacillin and piperacillin/tazobactam); cephalosporins (cefotaxime, ceftazidime, ceftazidime/Avibactam, cefepime, and ceftolozane/tazobactam); carbapenems (imipenem, meropenem, and ertapenem); lipopeptides (colistin); aminoglycosides (amikacin); fluoroquinolones (ciprofloxacin and levofloxacin); and folate pathway antagonists (trimethoprim/sulfamethoxazole). In addition, susceptibility to tigecycline (TG), chloramphenicol, and fosfomycin was tested. According to the definitions proposed by Magiorakos et al. [107], the isolates were classified into multidrug-resistant (MDR), extensively drug-resistant (XDR), and pan-drug-resistant (PDR). Isolates that were non-susceptible to at least one agent in three or more antimicrobial classes were considered MDR, while the XDR phenotype was assigned to the isolates that were susceptible to only one or two antimicrobial classes. PDR was defined as non-susceptibility to all agents in all antimicrobial classes.

### 4.3. DNA Extraction, Library Preparation, and Whole-Genome Sequencing

Genomic DNA was extracted from pure colonies using a High Pure PCR Template Preparation Kit (Roche Diagnostics GmbH, Mannheim, Germany) according to the manufacturer’s instructions. DNA was sequenced using Illumina MiSeq technology. For this purpose, the genomic libraries of the samples were prepared using a Nextera XT library preparation kit (Illumina Inc., San Diego, CA, USA). The samples were run on a MiSeq platform (Illumina Inc., San Diego, CA, USA) in paired-end mode as previously described [86].

### 4.4. Genome Assembly and Annotation

The raw read quality was assessed using FASTQC v0.11.7 (https://www.bioinformatics.babraham.ac.uk/projects/fastqc/, accessed on 1 July 2022) and de novo assemblies were generated with Shovill v.1.0.4 (https://github.com/tseemann/shovill, accessed on 1 July 2022) using SPAdes. Assembly statistics were assessed with QUAST v5.0.2 [108] and coding sequences were annotated using Prokka v.1.14.5 [109].

### 4.5. Multi-Locus Sequence Typing and Phylogenetic Analysis

For multi-locus sequence typing of the isolates, an in silico approach was chosen. Using the tool mlst v2.19.0 (https://github.com/tseemann/mlst) with the schemes Pasteur and Oxford deposited in PubMLST [110] (accessed on 6 July 2022), the strains were assigned to MLST sequence types (STs). GoeBURST analysis was used to group the STs of the isolates into clonal complexes (CCs) that were then allocated to the GCs described previously [1,18,20,26,111,112]. To do this, a minimum spanning tree combining all allelic profiles of both schemes was created using Phyloviz software v2.0 [113].

For a more in-depth strain differentiation, core genome single nucleotide polymorphisms (cgSNPs) were analysed. Here, data from foreign strains deposited in NCBI’s Sequence Read Archive were also included (Table S1). SNP calling was carried out with Snippy v.4.6.0 (https://github.com/tseemann/snippy, accessed on 1 July 2022) using *A. baumannii* strain ATCC 17978 (GCF_001593425.2) as reference genome. The alignment of core genome SNPs was used as input for RAxML v8.2.12 [114] for Maximum likelihood analysis using the GTRGAMMA model of rate heterogeneity. The cgSNP-based phylogenetic tree was visualised using the interactive tree of life (iTOL) online tool v6.7 (https://itol.embl.de/itol.cgi, accessed on 12 March 2023).

### 4.6. Screening of Antimicrobial Resistance Determinants

Abricate v0.8.10 (https://github.com/tseemann/abricate, accessed on 1 July 2022) was used for screening the newly assembled isolates for potential antimicrobial resistance determinants. For this purpose, the databases Resfinder [115], CARD [116], and AMRFinder [117] were used. Mutations previously linked to reduced susceptibility to fluoroquinolones, colistin, and TG were investigated. With regard to the corresponding genes in *A. baumannii* ATCC 19606, mutations were analysed using the MSA and SNP analysis tool provided by the Bacterial and Viral Bioinformatics Resource Center (BV-BRC), available at: https://www.bv-brc.org/ (accessed on 1 March 2023). Multiple sequence alignments of the predicted amino acid sequences were done using the Clustal Omega (1.2.4) multiple sequence alignment service provided by the EMBL’s European Bioinformatics Institute (EMBL-EBI) website (https://www.ebi.ac.uk/Tools/msa/clustalo/, accessed on 12 March 2023) and visualised in a coloured alignment format using the multiple alignment viewer tool MView 1.63, also hosted by EMBL-EBI and available at (https://www.ebi.ac.uk/Tools/msa/mview/, accessed on 12 March 2023).

### 4.7. Statistical Analysis

Chi-square and Fisher’s Exact Tests were used for assessing the association between categorical variables such as resistance phenotypes, resistance determinants, GCs, and STs. All tests were two-tailed and were performed using IBM Statistical Package for the Social Sciences (SPSS) Statistics for Windows version 20.0 (IBM Corp., Armonk, NY, USA). *p*-values ≤ 0.05 were considered statistically significant.

## 5. Conclusions

Here, we demonstrated the contribution of five GCs in *A. baumannii* infections in Egypt, all showing MDR, XDR, or PDR phenotypes. We defined the resistome structure of the isolates and the potential contribution of specific resistance mechanisms. In more detail, we analysed the genetic background of non-susceptibility to last-line antimicrobials in silico. The association of different combinations of resistance mechanisms with resistance phenotypes was also examined. A preliminary image was drawn for the GC-specific resistomes.

## Figures and Tables

**Table 1:** Correlation between potential carbapenem resistance determinants and carbapenem resistance.

Carbapenem Resistance Determinants	No. of Isolates	GC	IMP^R^	MER^R^
None	2	2	100.0%	100.0%
IS*Aba1-*preceded *bla*_OXA-51-like_ only	5	2	100.0%	100.0%
IS*Aba1-*preceded *bla*_ADC_ only	4	2	100.0%	100.0%
*bla*_OXA-23_ only	1	7	100.0%	100.0%
IS*Aba1-*preceded *bla*_OXA-51-like_ + IS*Aba1-*preceded *bla*_ADC_	2	2	100.0%	100.0%
*bla*_OXA-23_ + IS*Aba1-*preceded *bla*_OXA-51-like_	1	2	100.0%	100.0%
*bla*_OXA-23_ + IS*Aba1-*preceded *bla*_ADC_	9	2 and 4	100.0%	100.0%
*bla*_NDM-1_ + IS*Aba1-*preceded *bla*_OXA-51-like_	1	9	0.0%	0.0%
*bla*_OXA-23_ + *bla*_NDM-1_	1	2	100.0%	100.0%
*bla*_OXA-23_ + *bla*_GES-11_	9	5 and 9	44.4%	55.6%
*bla*_OXA-23_ + IS*Aba1-*preceded *bla*_OXA-51-like_ + IS*Aba1-*preceded *bla*_ADC_	1	2	100.0%	100.0%
*bla*_OXA-23_ + *bla*_NDM-1_ + IS*Aba1-*preceded *bla*_ADC_	8	2	100.0%	100.0%
*bla*_OXA-23_ + *bla*_GES-11_ + IS*Aba1-*preceded *bla*_ADC_	1	5	0.0%	0.0%
*bla*_OXA-23_ + *bla*_GES-35_ + IS*Aba1-*preceded *bla*_ADC_	1	2	100.0%	100.0%

IMP^R^, imipenem resistance and MER^R^, meropenem resistance.

**Table 2:** Amikacin resistance genes found in the Egyptian *A. baumannii* isolates and their correlation to observed amikacin resistance.

Aminoglycoside Resistance Determinants	No. of Isolates	GC	AMK^R^
None	15	2 and 9	86.7%
*aph(3′)-VI* only	3	2 and 4	100.0%
*aac(6′)-Ib10* only	2	5	50.0%
*armA* only	2	2 and 7	50.0%
*armA + aac(6′)-Ib9*	2	2	100.0%
*armA + aph(3′)-VI*	6	2 and 4	66.7%
*aph(3′)-VI + aac(6′)-Ib10*	7	5 and 9	0.0%
*aph(3′)-VI + aac(6′)-Ib3*	1	5	0.0%
*aph(3′)-VI + aac(6′)-Ib10 + armA*	1	2	0.0%
*aph(3′)-VI + aac(6′)-Ib9 + armA*	7	2	100.0%

AMK^R^, amikacin resistance.

**Table 3:** Categories of antimicrobial resistance genes detected in Egyptian *A. baumannii* isolates based on their occurrence in different GCs.

GC	Unique	Fully Conserved	Not Fully Conserved
*bla*_OXA-23_/*bla*_TEM-1_-positive GC2	*aph(3′)-Ia/bla*_TEM-1_ */bla*_ADC-73_ */bla*_GES-35_ */catA1/catB8/tetA*	*aph(3′)-Ia/bla*_TEM-1_*/bla*_OXA-66_*/bla*_OXA-23_*/*IS*Aba1*-*bla*_ADC_	*aac(3)-Ia/aac(6′)-Ib/ant(3″)-Ia/aph(3″)-Ib/aph(3′)-VI/aph(6)-Id/armA/bla*_ADC-30_ ^*^/bla_ADC-73_*/bla*_NDM-1_*/bla*_GES-35_*/*IS*Aba1*-*bla*_OXA-51-like_*/catA1/catB8/mphE/msrE/tetA/tetB/sul1/sul2/dfrA7*
*bla*_OXA-23_/*bla*_TEM-1_-negative GC2	*bla*_ADC-33_ */bla*_OXA-82_		*aac(3)-Ia/ant(3″)-Ia/aph(3′)-VI/bla*_ADC-30_ ^*^/bla_ADC-33_*/bla*_OXA-66_*/bla*_OXA-82_*/bla*_OXA-23_*/*IS*Aba1*-*bla*_OXA-51-like_*/*IS*Aba1*-*bla*_ADC_*/sul1/sul2*
GC4	*bla*_ADC-2_ */bla*_OXA-51_ */bla*_PER-7_	*ant(2″)-Ia/aph(3″)-Ib/aph(3′)-VI/bla*_ADC-2_*/bla*_OXA-51_*/bla*_OXA-23_*/*IS*Aba1*-*bla*_ADC_	*armA/bla*_PER-7_ */cmlA5/mphE/msrE/tetB/sul1/sul2/arr2*
GC5	*bla*_ADC-32_ */bla*_OXA-378_	*aac(6′)-Ib/ant(3″)-Ia/ant(2″)-Ia/aph(3″)-Ib/aph(6)-Id/bla*_ADC-32_ */bla*_OXA-378_ */bla*_OXA-23_ */bla*_GES-11_ */cmlA5/sul1/dfrA7*	*aph(3′)-VI/*IS*Aba1*-*bla*_ADC_
GC9	*bla*_ADC-32_ */bla*_OXA-94_	*bla*_OXA-94_	*aac(6′)-Ib/aph(3′)-VI/bla*_ADC-32_*/bla*_NDM-1_*/bla*_OXA-23_*/bla*_GES-11_*/*IS*Aba1*-*bla*_OXA-51-like_*/mphE/sul1/sul2/dfrA7*

*, the gene is unique for GC2.

## Data Availability

The raw sequencing data were uploaded to the European Nucleotide Archive and are available under the project PRJEB58012.

## Supplementary Materials

The following supporting information can be downloaded at: https://www.mdpi.com/article/10.3390/antibiotics12071149/s1, Table S1: SRA accessions and metadata of foreign strains included in the cgSNP-based phylogeny analysis; Table S2: Metadata and STs of the isolates included in the current study; Table S3: Post-assembly metrics of the draft genomes generated in the current study; Figure S1: A minimum spanning tree based on Pasteur MLST typing scheme of *A. baumannii*; Figure S2: A minimum spanning tree based on Oxford MLST typing scheme of *A. baumannii*; Figure S3: Multiple sequence alignment of the predicted amino acid sequences of CarO in all isolates compared with that of *A. baumannii* ATCC 19606; Figure S4: Multiple sequence alignment of the predicted amino acid sequences of AdeR in all isolates compared with that of *A. baumannii* ATCC 19606; Figure S5: Multiple sequence alignment of the predicted amino acid sequences of AdeS in all isolates compared with that of *A. baumannii* ATCC 19606; Figure S6: Multiple sequence alignment of the predicted amino acid sequences of BaeR in all isolates compared with that of *A. baumannii* ATCC 19606; Figure S7: Multiple sequence alignment of the predicted amino acid sequences of BaeS in all isolates compared with that of *A. baumannii* ATCC 19606; Table S4: Summary of mutations affecting *adeRS* and *baeRS* and correlation to tigecycline resistance.
