# Supplementary material for: Acinetobacter baumannii Global Clone-Specific Resistomes Explored in Clinical Isolates Recovered from Egypt
Source: Antibiotics (Basel). 2023 Jul 4;12(7):1149. doi: 10.3390/antibiotics12071149 (PMC10376554; doi:10.3390/antibiotics12071149)
Supplement: Supplementary file 1 [file antibiotics-12-01149-s001.zip › antibiotics-2469038-supplementary.pdf]

Supplementary material

Table S1. SRA accessions and metadata of foreign strains included in the cgSNP-based phylogeny analysis.

Strain	SRA	Country	Year	Specimen	Oxford ST	Pasteur ST
**AYP-A2**	SRR5891495	Australia	2013	wound	1806, 208	2
**11A1213CRGN008**	SRR5739056	Canada	2012	NA	1806, 208	2
**11A1314CRGN088**	SRR5739066	Canada	2013	NA	1806, 208	2
**09A16CRGN003B**	SRR5739115	Canada	2016	NA	1806, 208	2
**AB1251**	SRR11589159	Egypt	2013	NA	1816, 195	2
**M17**	SRR14102306	Egypt	2020	sputum	1050, 2058	2
**M18**	SRR14102521	Egypt	2020	blood	1078	85
**A1829**	SRR13327188	Egypt	2018	blood	NA	NA
**A1825**	SRR13327192	Egypt	2018	bronchoalveolar lavage	2246	113
**A1824**	SRR13327193	Egypt	2018	bronchoalveolar lavage	1705	2
**A1823**	SRR13327194	Egypt	2018	bronchoalveolar lavage	2246	113
**A1819**	SRR13327199	Egypt	2018	blood	2805, 684	570
**A1813**	SRR13327205	Egypt	2018	blood	1816, 195	2
**M11**	SRR14100602	Egypt	2020	Pleural fluid	1089	85
**A1702**	SRR13327208	Egypt	2017	blood	NA	NA
**B9**	SRR15911746	France	NA	Seine River	1806/208	2
**AB1318**	SRR11589121	Israel	2014	NA	1806, 208	2
**M012.6**	SRR15973856	Israel	2019	NA	1816, 195	2
**AB1317**	SRR11589122	Israel	2014	NA	2054, 502	2
**AB1181**	SRR11589183	Israel	2010	NA	1806, 208	604
**AB1342**	SRR11589278	Israel	2014	NA	1808, 348	2
**Ab105**	SRR16951843	Israel	2019	blood	1806, 208	2
**AB1218**	SRR11589173	Jordan	2012	NA	1816, 195	2
**nasal1**	SRR15185780	Jordan	2016	Nasal swab	1806, 208	2
**blood3**	SRR15185781	Jordan	2016	blood	499	158
**ACN-20190830-N22**	SRR12905336	Libya	2019	wound	440	25
**ACN-20190830-N60**	SRR12905337	Libya	2019	wound	NA	NA
**ACN-20190830-N54**	SRR12905338	Libya	2019	blood	2806, 286	2
**ACN-20190830-N45**	SRR12905339	Libya	2019	throat swab	NA	NA
**ACN-20190830-N27**	SRR12905341	Libya	2019	wound	1806, 208	2
**AbBAS-1**	SRR12349738	Spain	2019	NA	957	85
**FDAARGOS_1360**	SRR13674446	USA	NA	NA	1114, 1841	2
**CFSAN0937**	SRR9883198	USA	2005	NA	1806, 208	2
**ORAB01**	SRR1819797	USA	2012	NA	1806, 208	2
**ABUH763**	SRR3666894	USA	2015	NA	1839, 281	2

Table S2: Metadata and STs of the isolates included in the current study

Strain	Specimen	Oxford STs	Pasteur STs
**20Y0020**	Endotracheal Tube	208, 1806	2
**20Y0021**	Blood	732	602
**20Y0022**	Endotracheal Tube	732	602
**20Y0023**	Wound swab	281	2
**20Y0024**	Endotracheal Tube	281	2
**20Y0025**	Catheter Tip	281	2
**20Y0026**	Wound swab	281	2
**20Y0028**	Blood	208, 1806	2
**20Y0030**	Catheter Tip	208, 1806	2
**20Y0031**	Endotracheal Tube	208, 1806	2
**20Y0032**	Sputum	208, 1806	2
**20Y0033**	Endotracheal Tube	1114, 1841	2
**20Y0035**	Sputum	732	602
**20Y0036**	Catheter Tip	281	2
**20Y0037**	Sputum	684, 2805	570
**20Y0038**	Blood	281	2
**20Y0039**	Endotracheal Tube	281	2
**20Y0040**	Wound swab	281	2
**20Y0041**	Sputum	684, 2805	570
**20Y0042**	Endotracheal Tube	208, 1806	2
**20Y0043**	Wound swab	684, 2805	570
**20Y0045**	Sputum	732	602
**20Y0046**	Endotracheal Tube	732	602
**20Y0048**	Blood	2026, 2807	570
**20Y0054**	Blood	208, 1806	604
**20Y0055**	Blood	195, 1816	2
**20Y0056**	Blood	195, 1816	2
**20Y0057**	Blood	2808	85
**20Y0058**	Sputum	732	602
**20Y0059**	Blood	195, 1816	2
**20Y0060**	Blood	195, 1816	2
**20Y0062**	Blood	502, 2054	600
**20Y0063**	Blood	1050, 2058	2
**20Y0066**	Blood	2026, 2807	570
**20Y0067**	Blood	2026, 2807	570
**20Y0068**	Blood	195, 1816	2
**20Y0070**	Blood	286, 2806	570
**20Y0075**	Endotracheal Tube	208, 1806	2
**20Y0076**	Sputum	732	602
**20Y0077**	Wound swab	2246	113
**20Y0078**	Blood	1115	15
**20Y0079**	Blood	1115	15
**20Y0080**	Blood	1580	85
**20Y0081**	Blood	732	602
**20Y0082**	Blood	732	602
**20Y0083**	Pus	1115	15

Table S3: Post-assembly metrics of the draft genomes generated in the current study

ID	# Contigs	GC (%)	Genome fraction (%)	L50	N50	Total Length (>= 0 bp)
**20Y0020**	124	39.12	82.464	17	63,013	3,949,756
**20Y0021**	120	38.97	84.246	19	67,691	4,052,277
**20Y0022**	76	38.95	84.320	8	180,645	4,067,317
**20Y0023**	169	39.08	82.365	25	46,184	3,798,591
**20Y0024**	88	39.02	82.789	12	86,520	3,815,456
**20Y0025**	83	39.02	82.606	12	105,392	3,816,242
**20Y0026**	164	39.07	82.573	23	51,975	3,804,414
**20Y0028**	112	39.11	82.328	15	83,486	3,954,504
**20Y0030**	116	39.11	82.477	15	98,676	3,937,117
**20Y0031**	144	39.13	82.214	21	49,789	3,935,324
**20Y0032**	76	39.10	82.561	11	133,123	3,946,657
**20Y0033**	133	39.02	83.217	18	76,709	4,067,300
**20Y0035**	123	38.99	84.143	11	88,807	4,064,120
**20Y0036**	116	39.02	82.915	14	89,001	3,855,350
**20Y0037**	85	38.99	82.079	12	110,515	3,868,356
**20Y0038**	147	38.95	82.780	19	61,103	3,918,603
**20Y0039**	120	39.02	82.885	15	75,801	3,855,053
**20Y0040**	143	39.05	82.584	17	61,060	3,844,840
**20Y0041**	111	39.01	81.837	17	79,886	3,864,308
**20Y0042**	150	39.15	82.192	22	61,206	3,932,666
**20Y0043**	90	38.99	81.748	14	99,619	3,862,699
**20Y0045**	124	38.99	84.231	15	73,045	4,061,666
**20Y0046**	96	38.94	84.355	11	122,870	4,061,701
**20Y0048**	121	38.91	81.782	17	74,174	3,942,770
**20Y0054**	107	38.90	82.705	16	85,520	4,036,875
**20Y0055**	88	38.86	82.258	18	78,512	3,960,451
**20Y0056**	112	38.89	82.195	16	82,756	3,950,723
**20Y0057**	144	39.02	81.283	15	70,792	3,978,669
**20Y0058**	77	38.95	84.427	12	105,476	4,066,863
**20Y0059**	187	38.90	82.697	15	110,212	4,247,432
**20Y0060**	82	38.86	82.098	13	110,241	3,957,597
**20Y0062**	126	38.97	81.891	15	81,618	4,108,098
**20Y0063**	130	38.96	82.611	17	61,439	3,992,810
**20Y0066**	116	39.01	81.726	16	65,743	3,871,707
**20Y0067**	150	39.06	81.417	24	54,557	3,865,554
**20Y0068**	107	39.00	82.190	17	84,571	3,883,930
**20Y0070**	146	39.05	81.852	21	55,067	3,865,723
**20Y0075**	95	38.87	82.312	18	79,920	3,969,708
**20Y0076**	108	38.95	84.284	15	79,196	4,051,733
**20Y0077**	163	39.08	84.662	20	51,316	4,161,645
**20Y0078**	152	39.00	86.573	24	51,538	4,136,194
**20Y0079**	133	38.97	86.861	22	56,881	4,142,505
**20Y0080**	129	38.86	82.687	15	78,173	3,979,601
**20Y0081**	82	38.93	84.349	12	97,676	4,053,101
**20Y0082**	128	38.98	84.191	19	61,878	4,041,800
**20Y0083**	152	38.96	86.455	24	51,962	4,108,172

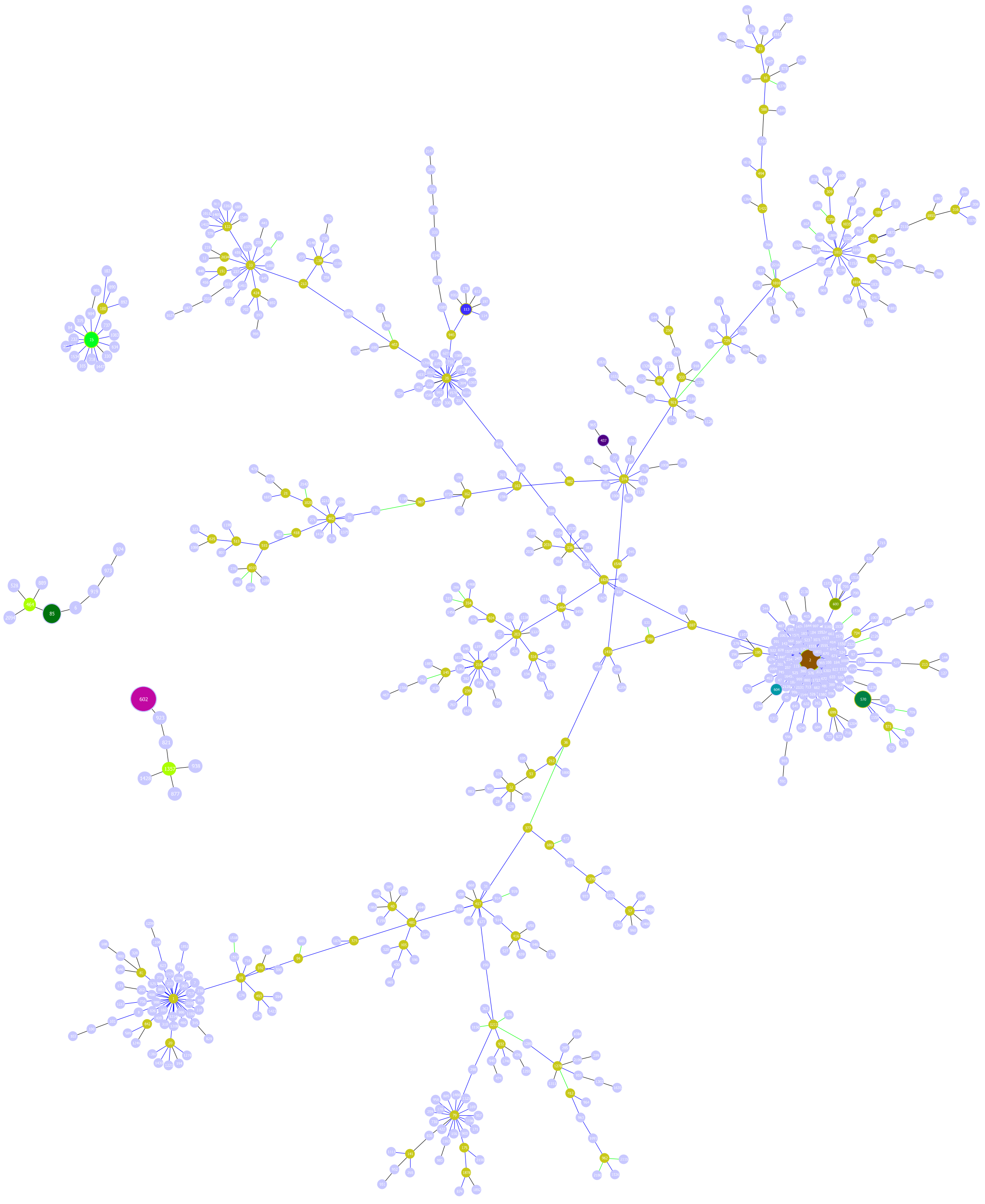

Figure S1: A minimum spanning tree based on Pasteur MLST typing scheme of *A. baumannii*. The numbers inside the circles refer to STs while light blue and light green circles correspond to STs in the database and clonal complexes, respectively. Circles of other colors correspond to the STs of the isolates sequenced in the current study.

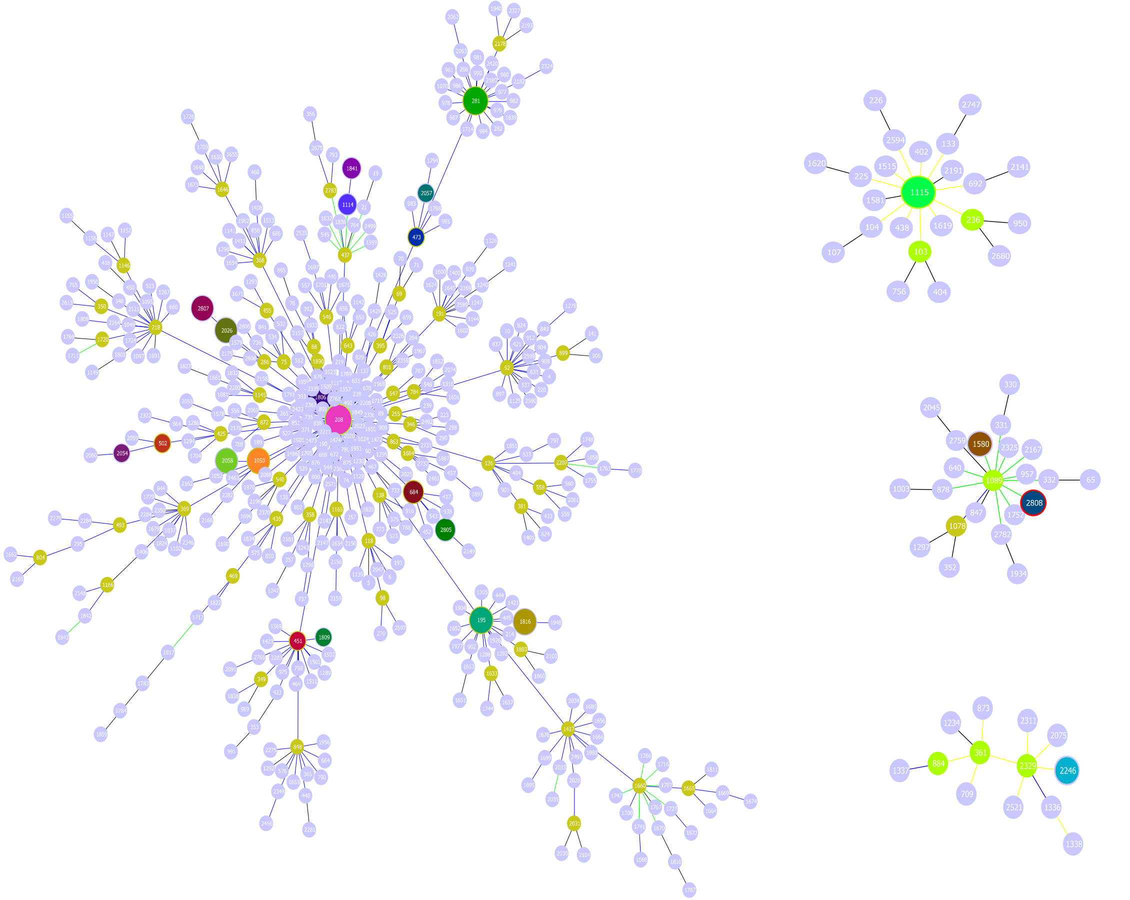

Figure S2: A minimum spanning tree based on Oxford MLST typing scheme of *A. baumannii*. The numbers inside the circles refer to STs while light blue and light green circles correspond to STs in the database and clonal complexes, respectively. Circles of other colors correspond to the STs of the isolates sequenced in the current study.

```
            cov   pid   1     10    20    30    40    50    60    70    80    90    100   110   120
                        +---------+---------+---------+---------+---------+---------+---------+---------+---------+---------+---------+---------+---------+
1 ATCC19606 100.0% 100.0%
MKVLRVLVTTTALLAAGAAMADEAVVHDSYAFDKNQLIPVGARAEVGTTGYGGALLWQANPYVGLALGYNGGDISWTDDVSVNGTKYDLDMDNNNVYLNAEIRPWGASTNPWAQGLYIAA
2 20Y0021   100.0% 100.0%
MKVLRVLVTTTALLAAGAAMADEAVVHDSYAFDKNQLIPVGARAEVGTTGYGGALLWQANPYVGLALGYNGGDISWTDDVSVNGTKYDLDMDNNNVYLNAEIRPWGASTNPWAQGLYIAA
3 20Y0082   100.0% 100.0%
MKVLRVLVTTTALLAAGAAMADEAVVHDSYAFDKNQLIPVGARAEVGTTGYGGALLWQANPYVGLALGYNGGDISWTDDVSVNGTKYDLDMDNNNVYLNAEIRPWGASTNPWAQGLYIAA
4 20Y0022   100.0% 100.0%
MKVLRVLVTTTALLAAGAAMADEAVVHDSYAFDKNQLIPVGARAEVGTTGYGGALLWQANPYVGLALGYNGGDISWTDDVSVNGTKYDLDMDNNNVYLNAEIRPWGASTNPWAQGLYIAA
5 20Y0035   100.0% 100.0%
MKVLRVLVTTTALLAAGAAMADEAVVHDSYAFDKNQLIPVGARAEVGTTGYGGALLWQANPYVGLALGYNGGDISWTDDVSVNGTKYDLDMDNNNVYLNAEIRPWGASTNPWAQGLYIAA
6 20Y0046   100.0% 100.0%
MKVLRVLVTTTALLAAGAAMADEAVVHDSYAFDKNQLIPVGARAEVGTTGYGGALLWQANPYVGLALGYNGGDISWTDDVSVNGTKYDLDMDNNNVYLNAEIRPWGASTNPWAQGLYIAA
7 20Y0057   100.0% 100.0%
MKVLRVLVTTTALLAAGAAMADEAVVHDSYAFDKNQLIPVGARAEVGTTGYGGALLWQANPYVGLALGYNGGDISWTDDVSVNGTKYDLDMDNNNVYLNAEIRPWGASTNPWAQGLYIAA
8 20Y0045   100.0% 100.0%
MKVLRVLVTTTALLAAGAAMADEAVVHDSYAFDKNQLIPVGARAEVGTTGYGGALLWQANPYVGLALGYNGGDISWTDDVSVNGTKYDLDMDNNNVYLNAEIRPWGASTNPWAQGLYIAA
9 20Y0058   100.0% 100.0%
MKVLRVLVTTTALLAAGAAMADEAVVHDSYAFDKNQLIPVGARAEVGTTGYGGALLWQANPYVGLALGYNGGDISWTDDVSVNGTKYDLDMDNNNVYLNAEIRPWGASTNPWAQGLYIAA
10 20Y0076   100.0% 100.0%
MKVLRVLVTTTALLAAGAAMADEAVVHDSYAFDKNQLIPVGARAEVGTTGYGGALLWQANPYVGLALGYNGGDISWTDDVSVNGTKYDLDMDNNNVYLNAEIRPWGASTNPWAQGLYIAA
11 20Y0080   100.0% 100.0%
MKVLRVLVTTTALLAAGAAMADEAVVHDSYAFDKNQLIPVGARAEVGTTGYGGALLWQANPYVGLALGYNGGDISWTDDVSVNGTKYDLDMDNNNVYLNAEIRPWGASTNPWAQGLYIAA
12 20Y0081   100.0% 100.0%
MKVLRVLVTTTALLAAGAAMADEAVVHDSYAFDKNQLIPVGARAEVGTTGYGGALLWQANPYVGLALGYNGGDISWTDDVSVNGTKYDLDMDNNNVYLNAEIRPWGASTNPWAQGLYIAA
13 20Y0020    98.8%  75.9%
MKVLRVLVTTTALLAAGAAMADEAVVHDSYAFDKNQLIPVGARAEVGTTGYGGALLWQANPYVGLALGYNGGDISWSDDVKVNGSTYDLDMDNNNVYLNAEIRPWGASTNRWAQGLYVAA
14 20Y0042    98.8%  75.9%
MKVLRVLVTTTALLAAGAAMADEAVVHDSYAFDKNQLIPVGARAEVGTTGYGGALLWQANPYVGLALGYNGGDISWSDDVKVNGSTYDLDMDNNNVYLNAEIRPWGASTNRWAQGLYVAA
15 20Y0048    98.8%  75.9%
MKVLRVLVTTTALLAAGAAMADEAVVHDSYAFDKNQLIPVGARAEVGTTGYGGALLWQANPYVGLALGYNGGDISWSDDVKVNGSTYDLDMDNNNVYLNAEIRPWGASTNRWAQGLYVAA
16 20Y0075    98.8%  75.9%
MKVLRVLVTTTALLAAGAAMADEAVVHDSYAFDKNQLIPVGARAEVGTTGYGGALLWQANPYVGLALGYNGGDISWSDDVKVNGSTYDLDMDNNNVYLNAEIRPWGASTNRWAQGLYVAA
17 20Y0041    98.8%  75.9%
MKVLRVLVTTTALLAAGAAMADEAVVHDSYAFDKNQLIPVGARAEVGTTGYGGALLWQANPYVGLALGYNGGDISWSDDVKVNGSTYDLDMDNNNVYLNAEIRPWGASTNRWAQGLYVAA
18 20Y0070    98.8%  75.9%
MKVLRVLVTTTALLAAGAAMADEAVVHDSYAFDKNQLIPVGARAEVGTTGYGGALLWQANPYVGLALGYNGGDISWSDDVKVNGSTYDLDMDNNNVYLNAEIRPWGASTNRWAQGLYVAA
19 20Y0067    98.8%  75.9%
MKVLRVLVTTTALLAAGAAMADEAVVHDSYAFDKNQLIPVGARAEVGTTGYGGALLWQANPYVGLALGYNGGDISWSDDVKVNGSTYDLDMDNNNVYLNAEIRPWGASTNRWAQGLYVAA
20 20Y0063    98.8%  75.9%
MKVLRVLVTTTALLAAGAAMADEAVVHDSYAFDKNQLIPVGARAEVGTTGYGGALLWQANPYVGLALGYNGGDISWSDDVKVNGSTYDLDMDNNNVYLNAEIRPWGASTNRWAQGLYVAA
21 20Y0054    98.8%  75.9%
MKVLRVLVTTTALLAAGAAMADEAVVHDSYAFDKNQLIPVGARAEVGTTGYGGALLWQANPYVGLALGYNGGDISWSDDVKVNGSTYDLDMDNNNVYLNAEIRPWGASTNRWAQGLYVAA
22 20Y0056    98.8%  75.9%
MKVLRVLVTTTALLAAGAAMADEAVVHDSYAFDKNQLIPVGARAEVGTTGYGGALLWQANPYVGLALGYNGGDISWSDDVKVNGSTYDLDMDNNNVYLNAEIRPWGASTNRWAQGLYVAA
23 20Y0068    98.8%  75.9%
MKVLRVLVTTTALLAAGAAMADEAVVHDSYAFDKNQLIPVGARAEVGTTGYGGALLWQANPYVGLALGYNGGDISWSDDVKVNGSTYDLDMDNNNVYLNAEIRPWGASTNRWAQGLYVAA
24 20Y0055    98.8%  75.9%
MKVLRVLVTTTALLAAGAAMADEAVVHDSYAFDKNQLIPVGARAEVGTTGYGGALLWQANPYVGLALGYNGGDISWSDDVKVNGSTYDLDMDNNNVYLNAEIRPWGASTNRWAQGLYVAA
25 20Y0066    98.8%  75.9%
MKVLRVLVTTTALLAAGAAMADEAVVHDSYAFDKNQLIPVGARAEVGTTGYGGALLWQANPYVGLALGYNGGDISWSDDVKVNGSTYDLDMDNNNVYLNAEIRPWGASTNRWAQGLYVAA
26 20Y0059    98.8%  75.9%
MKVLRVLVTTTALLAAGAAMADEAVVHDSYAFDKNQLIPVGARAEVGTTGYGGALLWQANPYVGLALGYNGGDISWSDDVKVNGSTYDLDMDNNNVYLNAEIRPWGASTNRWAQGLYVAA
27 20Y0030    98.8%  75.9%
MKVLRVLVTTTALLAAGAAMADEAVVHDSYAFDKNQLIPVGARAEVGTTGYGGALLWQANPYVGLALGYNGGDISWSDDVKVNGSTYDLDMDNNNVYLNAEIRPWGASTNRWAQGLYVAA
28 20Y0028    98.8%  75.9%
MKVLRVLVTTTALLAAGAAMADEAVVHDSYAFDKNQLIPVGARAEVGTTGYGGALLWQANPYVGLALGYNGGDISWSDDVKVNGSTYDLDMDNNNVYLNAEIRPWGASTNRWAQGLYVAA
29 20Y0037    98.8%  75.9%
MKVLRVLVTTTALLAAGAAMADEAVVHDSYAFDKNQLIPVGARAEVGTTGYGGALLWQANPYVGLALGYNGGDISWSDDVKVNGSTYDLDMDNNNVYLNAEIRPWGASTNRWAQGLYVAA
30 20Y0043    98.8%  75.9%
MKVLRVLVTTTALLAAGAAMADEAVVHDSYAFDKNQLIPVGARAEVGTTGYGGALLWQANPYVGLALGYNGGDISWSDDVKVNGSTYDLDMDNNNVYLNAEIRPWGASTNRWAQGLYVAA
31 20Y0060    98.8%  75.9%
MKVLRVLVTTTALLAAGAAMADEAVVHDSYAFDKNQLIPVGARAEVGTTGYGGALLWQANPYVGLALGYNGGDISWSDDVKVNGSTYDLDMDNNNVYLNAEIRPWGASTNRWAQGLYVAA
32 20Y0033    98.8%  75.9%
MKVLRVLVTTTALLAAGAAMADEAVVHDSYAFDKNQLIPVGARAEVGTTGYGGALLWQANPYVGLALGYNGGDISWSDDVKVNGSTYDLDMDNNNVYLNAEIRPWGASTNRWAQGLYVAA
33 20Y0031    98.8%  75.9%
MKVLRVLVTTTALLAAGAAMADEAVVHDSYAFDKNQLIPVGARAEVGTTGYGGALLWQANPYVGLALGYNGGDISWSDDVKVNGSTYDLDMDNNNVYLNAEIRPWGASTNRWAQGLYVAA
34 20Y0032    98.8%  75.9%
MKVLRVLVTTTALLAAGAAMADEAVVHDSYAFDKNQLIPVGARAEVGTTGYGGALLWQANPYVGLALGYNGGDISWSDDVKVNGSTYDLDMDNNNVYLNAEIRPWGASTNRWAQGLYVAA
35 20Y0062    98.8%  75.9%
MKVLRVLVTTTALLAAGAAMADEAVVHDSYAFDKNQLIPVGARAEVGTTGYGGALLWQANPYVGLALGYNGGDISWSDDVKVNGSTYDLDMDNNNVYLNAEIRPWGASTNRWAQGLYVAA
36 20Y0078    98.8%  75.9%
MKVLRVLVTTTALLAAGAAMADEAVVHDSYAFDKNQLIPVGARAEVGTTGYGGALLWQANPYVGLALGYNGGDISWSDDVKVNGSTYDLDMDNNNVYLNAEIRPWGASTNRWAQGLYVAA
37 20Y0079    98.8%  75.9%
MKVLRVLVTTTALLAAGAAMADEAVVHDSYAFDKNQLIPVGARAEVGTTGYGGALLWQANPYVGLALGYNGGDISWSDDVKVNGSTYDLDMDNNNVYLNAEIRPWGASTNRWAQGLYVAA
38 20Y0083    98.8%  75.9%
MKVLRVLVTTTALLAAGAAMADEAVVHDSYAFDKNQLIPVGARAEVGTTGYGGALLWQANPYVGLALGYNGGDISWSDDVKVNGSTYDLDMDNNNVYLNAEIRPWGASTNRWAQGLYVAA
39 20Y0023    99.2%  73.1%
MKVLRVLVTTTALLAAGAAMADEAVVHDSYAFDKNQLIPVGARAEVGTTGYGGALLWQANPYVGLALGYNGGDISWSDDLSINGTKYDMDMDNKLAYLNAEIRPWGASTNPWAQGLYVAA
40 20Y0036    99.2%  73.1%
MKVLRVLVTTTALLAAGAAMADEAVVHDSYAFDKNQLIPVGARAEVGTTGYGGALLWQANPYVGLALGYNGGDISWSDDLSINGTKYDMDMDNKLAYLNAEIRPWGASTNPWAQGLYVAA
41 20Y0024    99.2%  73.1%
MKVLRVLVTTTALLAAGAAMADEAVVHDSYAFDKNQLIPVGARAEVGTTGYGGALLWQANPYVGLALGYNGGDISWSDDLSINGTKYDMDMDNKLAYLNAEIRPWGASTNPWAQGLYVAA
42 20Y0025    99.2%  73.1%
MKVLRVLVTTTALLAAGAAMADEAVVHDSYAFDKNQLIPVGARAEVGTTGYGGALLWQANPYVGLALGYNGGDISWSDDLSINGTKYDMDMDNKLAYLNAEIRPWGASTNPWAQGLYVAA
43 20Y0026    99.2%  73.1%
MKVLRVLVTTTALLAAGAAMADEAVVHDSYAFDKNQLIPVGARAEVGTTGYGGALLWQANPYVGLALGYNGGDISWSDDLSINGTKYDMDMDNKLAYLNAEIRPWGASTNPWAQGLYVAA
44 20Y0038    99.2%  73.1%
MKVLRVLVTTTALLAAGAAMADEAVVHDSYAFDKNQLIPVGARAEVGTTGYGGALLWQANPYVGLALGYNGGDISWSDDLSINGTKYDMDMDNKLAYLNAEIRPWGASTNPWAQGLYVAA
45 20Y0039    99.2%  73.1%
MKVLRVLVTTTALLAAGAAMADEAVVHDSYAFDKNQLIPVGARAEVGTTGYGGALLWQANPYVGLALGYNGGDISWSDDLSINGTKYDMDMDNKLAYLNAEIRPWGASTNPWAQGLYVAA
46 20Y0040    99.2%  73.1%
MKVLRVLVTTTALLAAGAAMADEAVVHDSYAFDKNQLIPVGARAEVGTTGYGGALLWQANPYVGLALGYNGGDISWSDDLSINGTKYDMDMDNKLAYLNAEIRPWGASTNPWAQGLYVAA
47 20Y0077    97.2%  78.3%
MKVLRVLVTTTALLAAGAAMADEAVVHDSYAFDKNQLIPVGARAEVGTTGYGGALLWQANPYVGLALGYNGGDISWRDDLSINGTKYDVDMDNNNVYLNAEIRPWGASTNRWAQGLYVAA
```

```
          cov   pid   121   130   140   150   160   170   180   190   200   210   220   230   240
                      +--------+---------+---------+---------+---------+---------+---------+---------+---------+---------+---------+---------+---------+
1 ATCC19606 100.0% 100.0%
GAAYLDNDYDLAKRIGNGDTLSIDGKNYQQAVPGQEGGVRGKMSYKNDIAPYLGFGFAPKISKNWGVFGEVGAYYTGNPKVELTQYNLAPVTGNPTSAQDAVDKEANEIRNDNKYEWMPV
2 20Y0021   100.0% 100.0%
GAAYLDNDYDLAKRIGNGDTLSIDGKNYQQAVPGQEGGVRGKMSYKNDIAPYLGFGFAPKISKNWGVFGEVGAYYTGNPKVELTQYNLAPVTGNPTSAQDAVDKEANEIRNDNKYEWMPV
3 20Y0082   100.0% 100.0%
GAAYLDNDYDLAKRIGNGDTLSIDGKNYQQAVPGQEGGVRGKMSYKNDIAPYLGFGFAPKISKNWGVFGEVGAYYTGNPKVELTQYNLAPVTGNPTSAQDAVDKEANEIRNDNKYEWMPV
4 20Y0022   100.0% 100.0%
GAAYLDNDYDLAKRIGNGDTLSIDGKNYQQAVPGQEGGVRGKMSYKNDIAPYLGFGFAPKISKNWGVFGEVGAYYTGNPKVELTQYNLAPVTGNPTSAQDAVDKEANEIRNDNKYEWMPV
5 20Y0035   100.0% 100.0%
GAAYLDNDYDLAKRIGNGDTLSIDGKNYQQAVPGQEGGVRGKMSYKNDIAPYLGFGFAPKISKNWGVFGEVGAYYTGNPKVELTQYNLAPVTGNPTSAQDAVDKEANEIRNDNKYEWMPV
6 20Y0046   100.0% 100.0%
GAAYLDNDYDLAKRIGNGDTLSIDGKNYQQAVPGQEGGVRGKMSYKNDIAPYLGFGFAPKISKNWGVFGEVGAYYTGNPKVELTQYNLAPVTGNPTSAQDAVDKEANEIRNDNKYEWMPV
7 20Y0057   100.0% 100.0%
GAAYLDNDYDLAKRIGNGDTLSIDGKNYQQAVPGQEGGVRGKMSYKNDIAPYLGFGFAPKISKNWGVFGEVGAYYTGNPKVELTQYNLAPVTGNPTSAQDAVDKEANEIRNDNKYEWMPV
8 20Y0045   100.0% 100.0%
GAAYLDNDYDLAKRIGNGDTLSIDGKNYQQAVPGQEGGVRGKMSYKNDIAPYLGFGFAPKISKNWGVFGEVGAYYTGNPKVELTQYNLAPVTGNPTSAQDAVDKEANEIRNDNKYEWMPV
9 20Y0058   100.0% 100.0%
GAAYLDNDYDLAKRIGNGDTLSIDGKNYQQAVPGQEGGVRGKMSYKNDIAPYLGFGFAPKISKNWGVFGEVGAYYTGNPKVELTQYNLAPVTGNPTSAQDAVDKEANEIRNDNKYEWMPV
10 20Y0076   100.0% 100.0%
GAAYLDNDYDLAKRIGNGDTLSIDGKNYQQAVPGQEGGVRGKMSYKNDIAPYLGFGFAPKISKNWGVFGEVGAYYTGNPKVELTQYNLAPVTGNPTSAQDAVDKEANEIRNDNKYEWMPV
11 20Y0080   100.0% 100.0%
GAAYLDNDYDLAKRIGNGDTLSIDGKNYQQAVPGQEGGVRGKMSYKNDIAPYLGFGFAPKISKNWGVFGEVGAYYTGNPKVELTQYNLAPVTGNPTSAQDAVDKEANEIRNDNKYEWMPV
12 20Y0081   100.0% 100.0%
GAAYLDNDYDLAKRIGNGDTLSIDGKNYQQAVPGQEGGVRGKMSYKNDIAPYLGFGFAPKISKNWGVFGEVGAYYTGNPKVELTQYNLAPVTGNPTSAQDAVDKEANEIRNDNKYEWMPV
13 20Y0020    98.8%  75.9%    GAAYLDNDYDLTRNVDATRSFRVNNQDFIAGADG--VKINGQMSYKNDIAPYLGFGFAPKINKNWGVFGEVGAYYTGNPTVKLVSSGSAVTTG-
DQSLEEAVNAEARKIANDDKYKWLPV
14 20Y0042    98.8%  75.9%    GAAYLDNDYDLTRNVDATRSFRVNNQDFIAGADG--VKINGQMSYKNDIAPYLGFGFAPKINKNWGVFGEVGAYYTGNPTVKLVSSGSAVTTG-
DQSLEEAVNAEARKIANDDKYKWLPV
15 20Y0048    98.8%  75.9%    GAAYLDNDYDLTRNVDATRSFRVNNQDFIAGADG--VKINGQMSYKNDIAPYLGFGFAPKINKNWGVFGEVGAYYTGNPTVKLVSSGSAVTTG-
DQSLEEAVNAEARKIANDDKYKWLPV
16 20Y0075    98.8%  75.9%    GAAYLDNDYDLTRNVDATRSFRVNNQDFIAGADG--VKINGQMSYKNDIAPYLGFGFAPKINKNWGVFGEVGAYYTGNPTVKLVSSGSAVTTG-
DQSLEEAVNAEARKIANDDKYKWLPV
17 20Y0041    98.8%  75.9%    GAAYLDNDYDLTRNVDATRSFRVNNQDFIAGADG--VKINGQMSYKNDIAPYLGFGFAPKINKNWGVFGEVGAYYTGNPTVKLVSSGSAVTTG-
DQSLEEAVNAEARKIANDDKYKWLPV
18 20Y0070    98.8%  75.9%    GAAYLDNDYDLTRNVDATRSFRVNNQDFIAGADG--VKINGQMSYKNDIAPYLGFGFAPKINKNWGVFGEVGAYYTGNPTVKLVSSGSAVTTG-
DQSLEEAVNAEARKIANDDKYKWLPV
19 20Y0067    98.8%  75.9%    GAAYLDNDYDLTRNVDATRSFRVNNQDFIAGADG--VKINGQMSYKNDIAPYLGFGFAPKINKNWGVFGEVGAYYTGNPTVKLVSSGSAVTTG-
DQSLEEAVNAEARKIANDDKYKWLPV
20 20Y0063    98.8%  75.9%    GAAYLDNDYDLTRNVDATRSFRVNNQDFIAGADG--VKINGQMSYKNDIAPYLGFGFAPKINKNWGVFGEVGAYYTGNPTVKLVSSGSAVTTG-
DQSLEEAVNAEARKIANDDKYKWLPV
21 20Y0054    98.8%  75.9%    GAAYLDNDYDLTRNVDATRSFRVNNQDFIAGADG--VKINGQMSYKNDIAPYLGFGFAPKINKNWGVFGEVGAYYTGNPTVKLVSSGSAVTTG-
DQSLEEAVNAEARKIANDDKYKWLPV
22 20Y0056    98.8%  75.9%    GAAYLDNDYDLTRNVDATRSFRVNNQDFIAGADG--VKINGQMSYKNDIAPYLGFGFAPKINKNWGVFGEVGAYYTGNPTVKLVSSGSAVTTG-
DQSLEEAVNAEARKIANDDKYKWLPV
23 20Y0068    98.8%  75.9%    GAAYLDNDYDLTRNVDATRSFRVNNQDFIAGADG--VKINGQMSYKNDIAPYLGFGFAPKINKNWGVFGEVGAYYTGNPTVKLVSSGSAVTTG-
DQSLEEAVNAEARKIANDDKYKWLPV
24 20Y0055    98.8%  75.9%    GAAYLDNDYDLTRNVDATRSFRVNNQDFIAGADG--VKINGQMSYKNDIAPYLGFGFAPKINKNWGVFGEVGAYYTGNPTVKLVSSGSAVTTG-
DQSLEEAVNAEARKIANDDKYKWLPV
25 20Y0066    98.8%  75.9%    GAAYLDNDYDLTRNVDATRSFRVNNQDFIAGADG--VKINGQMSYKNDIAPYLGFGFAPKINKNWGVFGEVGAYYTGNPTVKLVSSGSAVTTG-
DQSLEEAVNAEARKIANDDKYKWLPV
26 20Y0059    98.8%  75.9%    GAAYLDNDYDLTRNVDATRSFRVNNQDFIAGADG--VKINGQMSYKNDIAPYLGFGFAPKINKNWGVFGEVGAYYTGNPTVKLVSSGSAVTTG-
DQSLEEAVNAEARKIANDDKYKWLPV
27 20Y0030    98.8%  75.9%    GAAYLDNDYDLTRNVDATRSFRVNNQDFIAGADG--VKINGQMSYKNDIAPYLGFGFAPKINKNWGVFGEVGAYYTGNPTVKLVSSGSAVTTG-
DQSLEEAVNAEARKIANDDKYKWLPV
28 20Y0028    98.8%  75.9%    GAAYLDNDYDLTRNVDATRSFRVNNQDFIAGADG--VKINGQMSYKNDIAPYLGFGFAPKINKNWGVFGEVGAYYTGNPTVKLVSSGSAVTTG-
DQSLEEAVNAEARKIANDDKYKWLPV
29 20Y0037    98.8%  75.9%    GAAYLDNDYDLTRNVDATRSFRVNNQDFIAGADG--VKINGQMSYKNDIAPYLGFGFAPKINKNWGVFGEVGAYYTGNPTVKLVSSGSAVTTG-
DQSLEEAVNAEARKIANDDKYKWLPV
30 20Y0043    98.8%  75.9%    GAAYLDNDYDLTRNVDATRSFRVNNQDFIAGADG--VKINGQMSYKNDIAPYLGFGFAPKINKNWGVFGEVGAYYTGNPTVKLVSSGSAVTTG-
DQSLEEAVNAEARKIANDDKYKWLPV
31 20Y0060    98.8%  75.9%    GAAYLDNDYDLTRNVDATRSFRVNNQDFIAGADG--VKINGQMSYKNDIAPYLGFGFAPKINKNWGVFGEVGAYYTGNPTVKLVSSGSAVTTG-
DQSLEEAVNAEARKIANDDKYKWLPV
32 20Y0033    98.8%  75.9%    GAAYLDNDYDLTRNVDATRSFRVNNQDFIAGADG--VKINGQMSYKNDIAPYLGFGFAPKINKNWGVFGEVGAYYTGNPTVKLVSSGSAVTTG-
DQSLEEAVNAEARKIANDDKYKWLPV
33 20Y0031    98.8%  75.9%    GAAYLDNDYDLTRNVDATRSFRVNNQDFIAGADG--VKINGQMSYKNDIAPYLGFGFAPKINKNWGVFGEVGAYYTGNPTVKLVSSGSAVTTG-
DQSLEEAVNAEARKIANDDKYKWLPV
34 20Y0032    98.8%  75.9%    GAAYLDNDYDLTRNVDATRSFRVNNQDFIAGADG--VKINGQMSYKNDIAPYLGFGFAPKINKNWGVFGEVGAYYTGNPTVKLVSSGSAVTTG-
DQSLEEAVNAEARKIANDDKYKWLPV
35 20Y0062    98.8%  75.9%    GAAYLDNDYDLTRNVDATRSFRVNNQDFIAGADG--VKINGQMSYKNDIAPYLGFGFAPKINKNWGVFGEVGAYYTGNPTVKLVSSGSAVTTG-
DQSLEEAVNAEARKIANDDKYKWLPV
36 20Y0078    98.8%  75.9%    GAAYLDNDYDLTRNVDATRSFRVNNQDFIAGADG--VKINGQMSYKNDIAPYLGFGFAPKINKNWGVFGEVGAYYTGNPTVKLVSSGSAVTTG-
DQSLEKAVNAEARKIANDDKYKWLPV
37 20Y0079    98.8%  75.9%    GAAYLDNDYDLTRNVDATRSFRVNNQDFIAGADG--VKINGQMSYKNDIAPYLGFGFAPKINKNWGVFGEVGAYYTGNPTVKLVSSGSAVTTG-
DQSLEKAVNAEARKIANDDKYKWLPV
38 20Y0083    98.8%  75.9%    GAAYLDNDYDLTRNVDATRSFRVNNQDFIAGADG--VKINGQMSYKNDIAPYLGFGFAPKINKNWGVFGEVGAYYTGNPTVKLVSSGSAVTTG-
DQSLEKAVNAEARKIANDDKYKWLPV
39 20Y0023    99.2%  73.1%    GAAYVDNQYDLTKNVGTNASVEIDGNRFNGGANG--
VSIAGNLKYDNDIAPYIGFGFAPKFSKNWGVFGEVGAYYSGNPKVSLASNNDALIGSDGRTLGQTLDDQERKIANDDKYKWLPV
40 20Y0036    99.2%  73.1%    GAAYVDNQYDLTKNVGTNASVEIDGNRFNGGANG--
VSIAGNLKYDNDIAPYIGFGFAPKFSKNWGVFGEVGAYYSGNPKVSLASNNDALIGSDGRTLGQTLDDQERKIANDDKYKWLPV
41 20Y0024    99.2%  73.1%    GAAYVDNQYDLTKNVGTNASVEIDGNRFNGGANG--
VSIAGNLKYDNDIAPYIGFGFAPKFSKNWGVFGEVGAYYSGNPKVSLASNNDALIGSDGRTLGQTLDDQERKIANDDKYKWLPV
42 20Y0025    99.2%  73.1%    GAAYVDNQYDLTKNVGTNASVEIDGNRFNGGANG--
VSIAGNLKYDNDIAPYIGFGFAPKFSKNWGVFGEVGAYYSGNPKVSLASNNDALIGSDGRTLGQTLDDQERKIANDDKYKWLPV
43 20Y0026    99.2%  73.1%    GAAYVDNQYDLTKNVGTNASVEIDGNRFNGGANG--
VSIAGNLKYDNDIAPYIGFGFAPKFSKNWGVFGEVGAYYSGNPKVSLASNNDALIGSDGRTLGQTLDDQERKIANDDKYKWLPV
44 20Y0038    99.2%  73.1%    GAAYVDNQYDLTKNVGTNASVEIDGNRFNGGANG--
VSIAGNLKYDNDIAPYIGFGFAPKFSKNWGVFGEVGAYYSGNPKVSLASNNDALIGSDGRTLGQTLDDQERKIANDDKYKWLPV
45 20Y0039    99.2%  73.1%    GAAYVDNQYDLTKNVGTNASVEIDGNRFNGGANG--
VSIAGNLKYDNDIAPYIGFGFAPKFSKNWGVFGEVGAYYSGNPKVSLASNNDALIGSDGRTLGQTLDDQERKIANDDKYKWLPV
46 20Y0040    99.2%  73.1%    GAAYVDNQYDLTKNVGTNASVEIDGNRFNGGANG--
VSIAGNLKYDNDIAPYIGFGFAPKFSKNWGVFGEVGAYYSGNPKVSLASNNDALIGSDGRTLGQTLDDQERKIANDDKYKWLPV
```

47 20Y0077 97.2% 78.3% GAAYLDNDYDLTKRSSDG-TIKINGTNY-----SYNGSVNGQLSYKNDIAPYLGFGFAPKINKNWGVFGEVGAYYTGNPTVELDKQGTF-
VNAAGGNADADLRAEENKIRNDDKYKWLPV

		cov	pid	241 249
				+-------+
1	ATCC19606	100.0%	100.0%	GKVGVNFYW
2	20Y0021	100.0%	100.0%	GKVGVNFYW
3	20Y0082	100.0%	100.0%	GKVGVNFYW
4	20Y0022	100.0%	100.0%	GKVGVNFYW
5	20Y0035	100.0%	100.0%	GKVGVNFYW
6	20Y0046	100.0%	100.0%	GKVGVNFYW
7	20Y0057	100.0%	100.0%	GKVGVNFYW
8	20Y0045	100.0%	100.0%	GKVGVNFYW
9	20Y0058	100.0%	100.0%	GKVGVNFYW
10	20Y0076	100.0%	100.0%	GKVGVNFYW
11	20Y0080	100.0%	100.0%	GKVGVNFYW
12	20Y0081	100.0%	100.0%	GKVGVNFYW
13	20Y0020	98.8%	75.9%	GKVGVNFFW
14	20Y0042	98.8%	75.9%	GKVGVNFFW
15	20Y0048	98.8%	75.9%	GKVGVNFFW
16	20Y0075	98.8%	75.9%	GKVGVNFFW
17	20Y0041	98.8%	75.9%	GKVGVNFFW
18	20Y0070	98.8%	75.9%	GKVGVNFFW
19	20Y0067	98.8%	75.9%	GKVGVNFFW
20	20Y0063	98.8%	75.9%	GKVGVNFFW
21	20Y0054	98.8%	75.9%	GKVGVNFFW
22	20Y0056	98.8%	75.9%	GKVGVNFFW
23	20Y0068	98.8%	75.9%	GKVGVNFFW
24	20Y0055	98.8%	75.9%	GKVGVNFFW
25	20Y0066	98.8%	75.9%	GKVGVNFFW
26	20Y0059	98.8%	75.9%	GKVGVNFFW
27	20Y0030	98.8%	75.9%	GKVGVNFFW
28	20Y0028	98.8%	75.9%	GKVGVNFFW
29	20Y0037	98.8%	75.9%	GKVGVNFFW
30	20Y0043	98.8%	75.9%	GKVGVNFFW
31	20Y0060	98.8%	75.9%	GKVGVNFFW
32	20Y0033	98.8%	75.9%	GKVGVNFFW
33	20Y0031	98.8%	75.9%	GKVGVNFFW
34	20Y0032	98.8%	75.9%	GKVGVNFFW
35	20Y0062	98.8%	75.9%	GKVGVNFFW
36	20Y0078	98.8%	75.9%	GKVGVNFFW
37	20Y0079	98.8%	75.9%	GKVGVNFFW
38	20Y0083	98.8%	75.9%	GKVGVNFFW
39	20Y0023	99.2%	73.1%	GKVGVNFYW
40	20Y0036	99.2%	73.1%	GKVGVNFYW
41	20Y0024	99.2%	73.1%	GKVGVNFYW
42	20Y0025	99.2%	73.1%	GKVGVNFYW
43	20Y0026	99.2%	73.1%	GKVGVNFYW
44	20Y0038	99.2%	73.1%	GKVGVNFYW
45	20Y0039	99.2%	73.1%	GKVGVNFYW
46	20Y0040	99.2%	73.1%	GKVGVNFYW
47	20Y0077	97.2%	78.3%	GKVGVNFFW

Figure S3: Multiple sequence alignment of the predicted amino acid sequences of CarO in all isolates compared to that of *A. baumannii* ATCC 19606. The alignment was visualized using MView version 1.63. cov, coverage; pid, percent identity.

```
          cov    pid    1       10       20       30       40       50       60       70       80       90      100      110      120
                        +---------+---------+---------+---------+---------+---------+---------+---------+---------+---------+---------+---------+
1 ATCC19606 100.0% 100.0%
MFDHSFSFDCQDKVILVVEDDYDIGDIIENYLKREGMSVIRAMNGKQAIELHASQPIDLILLDIKLPELNGWEVLNKIRQKAQTPVIMLTALDQDIDKVMALRIGADDFVVKPFNPNEVV
2 20Y0020   100.0%  98.8%
MFDHSFSFDCQDKVILVVEDDYDIGDIIENYLKREGMSVIRAMNGKQAIELHASQPIDLILLDIKLPELNGWEVLNKIRQKAQTPVIMLTALDQDIDKVMALRIGADDFVVKPFNPNEVI
3 20Y0021   100.0%  98.0%
MFDHSFSFDCQDKVILVVEDDYDIGDIIENYLKREGMSVIRAMNGKQAIELHASQPIDLILLDIKLPELNGWEVLNKIRQKAQTPVIMLTALDQDIDKVMALRIGADDFVVKPFNPNEVI
4 20Y0023   100.0%  98.8%
MFDHSFSFDCQDKVILVVEDDYDIGDIIENYLKREGMSVIRAMNGKQAIELHASQPIDLILLDIKLPELNGWEVLNKIRQKAQTPVIMLTALDQDIDKVMALRIGADDFVVKPFNPNEVI
5 20Y0025   100.0%  98.8%
MFDHSFSFDCQDKVILVVEDDYDIGDIIENYLKREGMSVIRAMNGKQAIELHASQPIDLILLDIKLPELNGWEVLNKIRQKAQTPVIMLTALDQDIDKVMALRIGADDFVVKPFNPNEVI
6 20Y0024   100.0%  98.8%
MFDHSFSFDCQDKVILVVEDDYDIGDIIENYLKREGMSVIRAMNGKQAIELHASQPIDLILLDIKLPELNGWEVLNKIRQKAQTPVIMLTALDQDIDKVMALRIGADDFVVKPFNPNEVI
7 20Y0022   100.0%  98.0%
MFDHSFSFDCQDKVILVVEDDYDIGDIIENYLKREGMSVIRAMNGKQAIELHASQPIDLILLDIKLPELNGWEVLNKIRQKAQTPVIMLTALDQDIDKVMALRIGADDFVVKPFNPNEVI
8 20Y0060   100.0%  98.8%
MFDHSFSFDCQDKVILVVEDDYDIGDIIENYLKREGMSVIRAMNGKQAIELHASQPIDLILLDIKLPELNGWEVLNKIRQKAQTPVIMLTALDQDIDKVMALRIGADDFVVKPFNPNEVI
9 20Y0026   100.0%  98.8%
MFDHSFSFDCQDKVILVVEDDYDIGDIIENYLKREGMSVIRAMNGKQAIELHASQPIDLILLDIKLPELNGWEVLNKIRQKAQTPVIMLTALDQDIDKVMALRIGADDFVVKPFNPNEVI
10 20Y0032   100.0%  98.8%
MFDHSFSFDCQDKVILVVEDDYDIGDIIENYLKREGMSVIRAMNGKQAIELHASQPIDLILLDIKLPELNGWEVLNKIRQKAQTPVIMLTALDQDIDKVMALRIGADDFVVKPFNPNEVI
11 20Y0033   100.0%  98.8%
MFDHSFSFDCQDKVILVVEDDYDIGDIIENYLKREGMSVIRAMNGKQAIELHASQPIDLILLDIKLPELNGWEVLNKIRQKAQTPVIMLTALDQDIDKVMALRIGADDFVVKPFNPNEVI
12 20Y0035   100.0%  98.0%
MFDHSFSFDCQDKVILVVEDDYDIGDIIENYLKREGMSVIRAMNGKQAIELHASQPIDLILLDIKLPELNGWEVLNKIRQKAQTPVIMLTALDQDIDKVMALRIGADDFVVKPFNPNEVI
13 20Y0037   100.0%  98.8%
MFDHSFSFDCQDKVILVVEDDYDIGDIIENYLKREGMSVIRAMNGKQAIELHASQPIDLILLDIKLPELNGWEVLNKIRQKAQTPVIMLTALDQDIDKVMALRIGADDFVVKPFNPNEVI
14 20Y0038   100.0%  98.8%
MFDHSFSFDCQDKVILVVEDDYDIGDIIENYLKREGMSVIRAMNGKQAIELHASQPIDLILLDIKLPELNGWEVLNKIRQKAQTPVIMLTALDQDIDKVMALRIGADDFVVKPFNPNEVI
15 20Y0059   100.0%  98.8%
MFDHSFSFDCQDKVILVVEDDYDIGDIIENYLKREGMSVIRAMNGKQAIELHASQPIDLILLDIKLPELNGWEVLNKIRQKAQTPVIMLTALDQDIDKVMALRIGADDFVVKPFNPNEVI
16 20Y0039   100.0%  98.8%
MFDHSFSFDCQDKVILVVEDDYDIGDIIENYLKREGMSVIRAMNGKQAIELHASQPIDLILLDIKLPELNGWEVLNKIRQKAQTPVIMLTALDQDIDKVMALRIGADDFVVKPFNPNEVI
17 20Y0066   100.0%  98.8%
MFDHSFSFDCQDKVILVVEDDYDIGDIIENYLKREGMSVIRAMNGKQAIELHASQPIDLILLDIKLPELNGWEVLNKIRQKAQTPVIMLTALDQDIDKVMALRIGADDFVVKPFNPNEVI
18 20Y0078   100.0%  98.8%
MFDHSFSFDCQDKVILVVEDDYDIGDIIENYLKREGMSVIRAMNGKQAIELHASQPIDLILLDIKLPELNGWEVLNKIRQKAQTPVIMLTALDQDIDKVMALRIGADDFVVKPFNPNEVV
19 20Y0054   100.0%  98.8%
MFDHSFSFDCQDKVILVVEDDYDIGDIIENYLKREGMSVIRAMNGKQAIELHASQPIDLILLDIKLPELNGWEVLNKIRQKAQTPVIMLTALDQDIDKVMALRIGADDFVVKPFNPNEVI
20 20Y0055   100.0%  98.8%
MFDHSFSFDCQDKVILVVEDDYDIGDIIENYLKREGMSVIRAMNGKQAIELHASQPIDLILLDIKLPELNGWEVLNKIRQKAQTPVIMLTALDQDIDKVMALRIGADDFVVKPFNPNEVI
21 20Y0056   100.0%  98.8%
MFDHSFSFDCQDKVILVVEDDYDIGDIIENYLKREGMSVIRAMNGKQAIELHASQPIDLILLDIKLPELNGWEVLNKIRQKAQTPVIMLTALDQDIDKVMALRIGADDFVVKPFNPNEVI
22 20Y0058   100.0%  98.0%
MFDHSFSFDCQDKVILVVEDDYDIGDIIENYLKREGMSVIRAMNGKQAIELHASQPIDLILLDIKLPELNGWEVLNKIRQKAQTPVIMLTALDQDIDKVMALRIGADDFVVKPFNPNEVI
23 20Y0063   100.0%  98.8%
MFDHSFSFDCQDKVILVVEDDYDIGDIIENYLKREGMSVIRAMNGKQAIELHASQPIDLILLDIKLPELNGWEVLNKIRQKAQTPVIMLTALDQDIDKVMALRIGADDFVVKPFNPNEVI
24 20Y0067   100.0%  98.8%
MFDHSFSFDCQDKVILVVEDDYDIGDIIENYLKREGMSVIRAMNGKQAIELHASQPIDLILLDIKLPELNGWEVLNKIRQKAQTPVIMLTALDQDIDKVMALRIGADDFVVKPFNPNEVI
25 20Y0076   100.0%  98.0%
MFDHSFSFDCQDKVILVVEDDYDIGDIIENYLKREGMSVIRAMNGKQAIELHASQPIDLILLDIKLPELNGWEVLNKIRQKAQTPVIMLTALDQDIDKVMALRIGADDFVVKPFNPNEVI
26 20Y0070   100.0%  98.8%
MFDHSFSFDCQDKVILVVEDDYDIGDIIENYLKREGMSVIRAMNGKQAIELHASQPIDLILLDIKLPELNGWEVLNKIRQKAQTPVIMLTALDQDIDKVMALRIGADDFVVKPFNPNEVI
27 20Y0077   100.0%  97.2%
MFDHSFSFDCQDKVILVVEDDYDMGDIIENYLKREGMSVIRAMNGKQAIELHASQSIDLILLDIKLPELNGWEVLNKIRQKAQTPVIMLTALDQDIDKVMALRIGADDFVVKPFNPNEVI
28 20Y0079   100.0%  98.8%
MFDHSFSFDCQDKVILVVEDDYDIGDIIENYLKREGMSVIRAMNGKQAIELHASQPIDLILLDIKLPELNGWEVLNKIRQKAQTPVIMLTALDQDIDKVMALRIGADDFVVKPFNPNEVV
29 20Y0081   100.0%  98.0%
MFDHSFSFDCQDKVILVVEDDYDIGDIIENYLKREGMSVIRAMNGKQAIELHASQPIDLILLDIKLPELNGWEVLNKIRQKAQTPVIMLTALDQDIDKVMALRIGADDFVVKPFNPNEVI
30 20Y0057   100.0%  99.6%
MFDHSFSFDCQDKVILVVEDDYDIGDIIENYLKREGMSVIRAMNGKQAIELHASQPIDLILLDIKLPELNGWEVLNKIRQKAQTPVIMLTALDQDIDKVMALRIGADDFVVKPFNPNEVV
31 20Y0028   100.0%  98.8%
MFDHSFSFDCQDKVILVVEDDYDIGDIIENYLKREGMSVIRAMNGKQAIELHASQPIDLILLDIKLPELNGWEVLNKIRQKAQTPVIMLTALDQDIDKVMALRIGADDFVVKPFNPNEVI
32 20Y0041   100.0%  98.8%
MFDHSFSFDCQDKVILVVEDDYDIGDIIENYLKREGMSVIRAMNGKQAIELHASQPIDLILLDIKLPELNGWEVLNKIRQKAQTPVIMLTALDQDIDKVMALRIGADDFVVKPFNPNEVI
33 20Y0031   100.0%  98.8%
MFDHSFSFDCQDKVILVVEDDYDIGDIIENYLKREGMSVIRAMNGKQAIELHASQPIDLILLDIKLPELNGWEVLNKIRQKAQTPVIMLTALDQDIDKVMALRIGADDFVVKPFNPNEVI
34 20Y0030   100.0%  98.8%
MFDHSFSFDCQDKVILVVEDDYDIGDIIENYLKREGMSVIRAMNGKQAIELHASQPIDLILLDIKLPELNGWEVLNKIRQKAQTPVIMLTALDQDIDKVMALRIGADDFVVKPFNPNEVI
35 20Y0042   100.0%  98.8%
MFDHSFSFDCQDKVILVVEDDYDIGDIIENYLKREGMSVIRAMNGKQAIELHASQPIDLILLDIKLPELNGWEVLNKIRQKAQTPVIMLTALDQDIDKVMALRIGADDFVVKPFNPNEVI
36 20Y0068   100.0%  98.8%
MFDHSFSFDCQDKVILVVEDDYDIGDIIENYLKREGMSVIRAMNGKQAIELHASQPIDLILLDIKLPELNGWEVLNKIRQKAQTPVIMLTALDQDIDKVMALRIGADDFVVKPFNPNEVI
37 20Y0062   100.0%  98.8%
MFDHSFSFDCQDKVILVVEDDYDIGDIIENYLKREGMSVIRAMNGKQAIELHASQPIDLILLDIKLPELNGWEVLNKIRQKAQTPVIMLTALDQDIDKVMALRIGADDFVVKPFNPNEVI
38 20Y0075   100.0%  98.8%
MFDHSFSFDCQDKVILVVEDDYDIGDIIENYLKREGMSVIRAMNGKQAIELHASQPIDLILLDIKLPELNGWEVLNKIRQKAQTPVIMLTALDQDIDKVMALRIGADDFVVKPFNPNEVI
39 20Y0080   100.0%  99.6%
MFDHSFSFDCQDKVILVVEDDYDIGDIIENYLKREGMSVIRAMNGKQAIELHASQPIDLILLDIKLPELNGWEVLNKIRQKAQTPVIMLTALDQDIDKVMALRIGADDFVVKPFNPNEVV
40 20Y0082   100.0%  98.0%
MFDHSFSFDCQDKVILVVEDDYDIGDIIENYLKREGMSVIRAMNGKQAIELHASQPIDLILLDIKLPELNGWEVLNKIRQKAQTPVIMLTALDQDIDKVMALRIGADDFVVKPFNPNEVI
41 20Y0083   100.0%  98.8%
MFDHSFSFDCQDKVILVVEDDYDIGDIIENYLKREGMSVIRAMNGKQAIELHASQPIDLILLDIKLPELNGWEVLNKIRQKAQTPVIMLTALDQDIDKVMALRIGADDFVVKPFNPNEVV
42 20Y0043   100.0%  98.8%
MFDHSFSFDCQDKVILVVEDDYDIGDIIENYLKREGMSVIRAMNGKQAIELHASQPIDLILLDIKLPELNGWEVLNKIRQKAQTPVIMLTALDQDIDKVMALRIGADDFVVKPFNPNEVI
43 20Y0040   100.0%  98.8%
MFDHSFSFDCQDKVILVVEDDYDIGDIIENYLKREGMSVIRAMNGKQAIELHASQPIDLILLDIKLPELNGWEVLNKIRQKAQTPVIMLTALDQDIDKVMALRIGADDFVVKPFNPNEVI
44 20Y0045   100.0%  98.0%
MFDHSFSFDCQDKVILVVEDDYDIGDIIENYLKREGMSVIRAMNGKQAIELHASQPIDLILLDIKLPELNGWEVLNKIRQKAQTPVIMLTALDQDIDKVMALRIGADDFVVKPFNPNEVI
45 20Y0046   100.0%  98.0%
MFDHSFSFDCQDKVILVVEDDYDIGDIIENYLKREGMSVIRAMNGKQAIELHASQPIDLILLDIKLPELNGWEVLNKIRQKAQTPVIMLTALDQDIDKVMALRIGADDFVVKPFNPNEVI
46 20Y0048   100.0%  98.8%
MFDHSFSFDCQDKVILVVEDDYDIGDIIENYLKREGMSVIRAMNGKQAIELHASQPIDLILLDIKLPELNGWEVLNKIRQKAQTPVIMLTALDQDIDKVMALRIGADDFVVKPFNPNEVI
47 20Y0036   100.0%  98.8%
MFDHSFSFDCQDKVILVVEDDYDIGDIIENYLKREGMSVIRAMNGKQAIELHASQPIDLILLDIKLPELNGWEVLNKIRQKAQTPVIMLTALDQDIDKVMALRIGADDFVVKPFNPNEVI
```

cov pid 121 130 140 150 160 170 180 190 200 210 220 230 240

+--------+---------+---------+---------+---------+---------+---------+---------+---------+---------+---------+---------+---------+

1 ATCC19606 100.0% 100.0%
ARVQAVLRRTQFANKATNKNKLYKNIEIDTDTHSVYIHSENKKILLNLTLTEYKIISFMIDQPHKVFTRGELMNHCMNDSDALERTVDSHVSKLRKKLEEQGIFQMLINVRGVGYRLDNP

2 20Y0020 100.0% 98.8%
ARVQAVLRRTQFANKVTNKNKLYKNIEIDTDTHSVYIHSENKKILLNLTLTEYKIISFMIDQPHKVFTRGELMNHCMNDSDALERTVDSHVSKLRKKLEEQGIFQMLINVRGVGYRLDNP

3 20Y0021 100.0% 98.0%
ARVQAVLRRTQFANKATNKNKLYKNIEIDNDTHSVYILSENKKILLNLTLTEYKIISFMIDQPHKVFTRGELMNQCMNDSDALERTVDSHVSKLRKKLEEQGIFQMLINVRGVGYRLDNP

4 20Y0023 100.0% 98.8%
ARVQAVLRRTQFANKVTNKNKLYKNIEIDTDTHSVYIHSENKKILLNLTLTEYKIISFMIDQPHKVFTRGELMNHCMNDSDALERTVDSHVSKLRKKLEEQGIFQMLINVRGVGYRLDNP

5 20Y0025 100.0% 98.8%
ARVQAVLRRTQFANKVTNKNKLYKNIEIDTDTHSVYIHSENKKILLNLTLTEYKIISFMIDQPHKVFTRGELMNHCMNDSDALERTVDSHVSKLRKKLEEQGIFQMLINVRGVGYRLDNP

6 20Y0024 100.0% 98.8%
ARVQAVLRRTQFANKVTNKNKLYKNIEIDTDTHSVYIHSENKKILLNLTLTEYKIISFMIDQPHKVFTRGELMNHCMNDSDALERTVDSHVSKLRKKLEEQGIFQMLINVRGVGYRLDNP

7 20Y0022 100.0% 98.0%
ARVQAVLRRTQFANKATNKNKLYKNIEIDNDTHSVYILSENKKILLNLTLTEYKIISFMIDQPHKVFTRGELMNQCMNDSDALERTVDSHVSKLRKKLEEQGIFQMLINVRGVGYRLDNP

8 20Y0060 100.0% 98.8%
ARVQAVLRRTQFANKVTNKNKLYKNIEIDTDTHSVYIHSENKKILLNLTLTEYKIISFMIDQPHKVFTRGELMNHCMNDSDALERTVDSHVSKLRKKLEEQGIFQMLINVRGVGYRLDNP

9 20Y0026 100.0% 98.8%
ARVQAVLRRTQFANKVTNKNKLYKNIEIDTDTHSVYIHSENKKILLNLTLTEYKIISFMIDQPHKVFTRGELMNHCMNDSDALERTVDSHVSKLRKKLEEQGIFQMLINVRGVGYRLDNP

10 20Y0032 100.0% 98.8%
ARVQAVLRRTQFANKVTNKNKLYKNIEIDTDTHSVYIHSENKKILLNLTLTEYKIISFMIDQPHKVFTRGELMNHCMNDSDALERTVDSHVSKLRKKLEEQGIFQMLINVRGVGYRLDNP

11 20Y0033 100.0% 98.8%
ARVQAVLRRTQFANKVTNKNKLYKNIEIDTDTHSVYIHSENKKILLNLTLTEYKIISFMIDQPHKVFTRGELMNHCMNDSDALERTVDSHVSKLRKKLEEQGIFQMLINVRGVGYRLDNP

12 20Y0035 100.0% 98.0%
ARVQAVLRRTQFANKATNKNKLYKNIEIDNDTHSVYILSENKKILLNLTLTEYKIISFMIDQPHKVFTRGELMNQCMNDSDALERTVDSHVSKLRKKLEEQGIFQMLINVRGVGYRLDNP

13 20Y0037 100.0% 98.8%
ARVQAVLRRTQFANKVTNKNKLYKNIEIDTDTHSVYIHSENKKILLNLTLTEYKIISFMIDQPHKVFTRGELMNHCMNDSDALERTVDSHVSKLRKKLEEQGIFQMLINVRGVGYRLDNP

14 20Y0038 100.0% 98.8%
ARVQAVLRRTQFANKVTNKNKLYKNIEIDTDTHSVYIHSENKKILLNLTLTEYKIISFMIDQPHKVFTRGELMNHCMNDSDALERTVDSHVSKLRKKLEEQGIFQMLINVRGVGYRLDNP

15 20Y0059 100.0% 98.8%
ARVQAVLRRTQFANKVTNKNKLYKNIEIDTDTHSVYIHSENKKILLNLTLTEYKIISFMIDQPHKVFTRGELMNHCMNDSDALERTVDSHVSKLRKKLEEQGIFQMLINVRGVGYRLDNP

16 20Y0039 100.0% 98.8%
ARVQAVLRRTQFANKVTNKNKLYKNIEIDTDTHSVYIHSENKKILLNLTLTEYKIISFMIDQPHKVFTRGELMNHCMNDSDALERTVDSHVSKLRKKLEEQGIFQMLINVRGVGYRLDNP

17 20Y0066 100.0% 98.8%
ARVQAVLRRTQFANKVTNKNKLYKNIEIDTDTHSVYIHSENKKILLNLTLTEYKIISFMIDQPHKVFTRGELMNHCMNDSDALERTVDSHVSKLRKKLEEQGIFQMLINVRGVGYRLDNP

18 20Y0078 100.0% 98.8%
ARVQAVLRRTQFANKATNKNKIYKNIEIDTDTHSVYIHSENKKILLNLTLTEYKIISFMIDQPHKVFTRGELMNHCMNDSDALERTVDSHVSKLRKKLEEQGIFQMLINVRGVGYRLDNP

19 20Y0054 100.0% 98.8%
ARVQAVLRRTQFANKVTNKNKLYKNIEIDTDTHSVYIHSENKKILLNLTLTEYKIISFMIDQPHKVFTRGELMNHCMNDSDALERTVDSHVSKLRKKLEEQGIFQMLINVRGVGYRLDNP

20 20Y0055 100.0% 98.8%
ARVQAVLRRTQFANKVTNKNKLYKNIEIDTDTHSVYIHSENKKILLNLTLTEYKIISFMIDQPHKVFTRGELMNHCMNDSDALERTVDSHVSKLRKKLEEQGIFQMLINVRGVGYRLDNP

21 20Y0056 100.0% 98.8%
ARVQAVLRRTQFANKVTNKNKLYKNIEIDTDTHSVYIHSENKKILLNLTLTEYKIISFMIDQPHKVFTRGELMNHCMNDSDALERTVDSHVSKLRKKLEEQGIFQMLINVRGVGYRLDNP

22 20Y0058 100.0% 98.0%
ARVQAVLRRTQFANKATNKNKLYKNIEIDNDTHSVYILSENKKILLNLTLTEYKIISFMIDQPHKVFTRGELMNQCMNDSDALERTVDSHVSKLRKKLEEQGIFQMLINVRGVGYRLDNP

23 20Y0063 100.0% 98.8%
ARVQAVLRRTQFANKVTNKNKLYKNIEIDTDTHSVYIHSENKKILLNLTLTEYKIISFMIDQPHKVFTRGELMNHCMNDSDALERTVDSHVSKLRKKLEEQGIFQMLINVRGVGYRLDNP

24 20Y0067 100.0% 98.8%
ARVQAVLRRTQFANKVTNKNKLYKNIEIDTDTHSVYIHSENKKILLNLTLTEYKIISFMIDQPHKVFTRGELMNHCMNDSDALERTVDSHVSKLRKKLEEQGIFQMLINVRGVGYRLDNP

25 20Y0076 100.0% 98.0%
ARVQAVLRRTQFANKATNKNKLYKNIEIDNDTHSVYILSENKKILLNLTLTEYKIISFMIDQPHKVFTRGELMNQCMNDSDALERTVDSHVSKLRKKLEEQGIFQMLINVRGVGYRLDNP

26 20Y0070 100.0% 98.8%
ARVQAVLRRTQFANKVTNKNKLYKNIEIDTDTHSVYIHSENKKILLNLTLTEYKIISFMIDQPHKVFTRGELMNHCMNDSDALERTVDSHVSKLRKKLEEQGIFQMLINVRGVGYRLDNP

27 20Y0077 100.0% 97.2%
ARVQAVLRRTQFANKAANKNKLYKNIEIDTDTHSVYIHSENKKILLNLTLTEYKIISFMIDQPHKVFTRGELMNHCMNDSDALERTVDSHVSKLRKKLEEQGIFQMLVNVRGVGYRLDNP

28 20Y0079 100.0% 98.8%
ARVQAVLRRTQFANKATNKNKIYKNIEIDTDTHSVYIHSENKKILLNLTLTEYKIISFMIDQPHKVFTRGELMNHCMNDSDALERTVDSHVSKLRKKLEEQGIFQMLINVRGVGYRLDNP

29 20Y0081 100.0% 98.0%
ARVQAVLRRTQFANKATNKNKLYKNIEIDNDTHSVYILSENKKILLNLTLTEYKIISFMIDQPHKVFTRGELMNQCMNDSDALERTVDSHVSKLRKKLEEQGIFQMLINVRGVGYRLDNP

30 20Y0057 100.0% 99.6%
ARVQAVLRRTQFANKATNKNKLYKNIEIDTDTHSVYIHSENKKILLNLTLTEYKIISFMIDQPHKVFTRGELMNHCMNDSDALERTVDSHVSKLRKKLEEQGIFQMLINVRGVGYRLDNP

31 20Y0028 100.0% 98.8%
ARVQAVLRRTQFANKVTNKNKLYKNIEIDTDTHSVYIHSENKKILLNLTLTEYKIISFMIDQPHKVFTRGELMNHCMNDSDALERTVDSHVSKLRKKLEEQGIFQMLINVRGVGYRLDNP

32 20Y0041 100.0% 98.8%
ARVQAVLRRTQFANKVTNKNKLYKNIEIDTDTHSVYIHSENKKILLNLTLTEYKIISFMIDQPHKVFTRGELMNHCMNDSDALERTVDSHVSKLRKKLEEQGIFQMLINVRGVGYRLDNP

33 20Y0031 100.0% 98.8%
ARVQAVLRRTQFANKVTNKNKLYKNIEIDTDTHSVYIHSENKKILLNLTLTEYKIISFMIDQPHKVFTRGELMNHCMNDSDALERTVDSHVSKLRKKLEEQGIFQMLINVRGVGYRLDNP

34 20Y0030 100.0% 98.8%
ARVQAVLRRTQFANKVTNKNKLYKNIEIDTDTHSVYIHSENKKILLNLTLTEYKIISFMIDQPHKVFTRGELMNHCMNDSDALERTVDSHVSKLRKKLEEQGIFQMLINVRGVGYRLDNP

35 20Y0042 100.0% 98.8%
ARVQAVLRRTQFANKVTNKNKLYKNIEIDTDTHSVYIHSENKKILLNLTLTEYKIISFMIDQPHKVFTRGELMNHCMNDSDALERTVDSHVSKLRKKLEEQGIFQMLINVRGVGYRLDNP

36 20Y0068 100.0% 98.8%
ARVQAVLRRTQFANKVTNKNKLYKNIEIDTDTHSVYIHSENKKILLNLTLTEYKIISFMIDQPHKVFTRGELMNHCMNDSDALERTVDSHVSKLRKKLEEQGIFQMLINVRGVGYRLDNP

37 20Y0062 100.0% 98.8%
ARVQAVLRRTQFANKVTNKNKLYKNIEIDTDTHSVYIHSENKKILLNLTLTEYKIISFMIDQPHKVFTRGELMNHCMNDSDALERTVDSHVSKLRKKLEEQGIFQMLINVRGVGYRLDNP

38 20Y0075 100.0% 98.8%
ARVQAVLRRTQFANKVTNKNKLYKNIEIDTDTHSVYIHSENKKILLNLTLTEYKIISFMIDQPHKVFTRGELMNHCMNDSDALERTVDSHVSKLRKKLEEQGIFQMLINVRGVGYRLDNP

39 20Y0080 100.0% 99.6%
ARVQAVLRRTQFANKATNKNKLYKNIEIDTDTHSVYIHSENKKILLNLTLTEYKIISFMIDQPHKVFTRGELMNHCMNDSDALERTVDSHVSKLRKKLEEQGIFQMLINVRGVGYRLDNP

40 20Y0082 100.0% 98.0%
ARVQAVLRRTQFANKATNKNKLYKNIEIDNDTHSVYILSENKKILLNLTLTEYKIISFMIDQPHKVFTRGELMNQCMNDSDALERTVDSHVSKLRKKLEEQGIFQMLINVRGVGYRLDNP

41 20Y0083 100.0% 98.8%
ARVQAVLRRTQFANKATNKNKIYKNIEIDTDTHSVYIHSENKKILLNLTLTEYKIISFMIDQPHKVFTRGELMNHCMNDSDALERTVDSHVSKLRKKLEEQGIFQMLINVRGVGYRLDNP

42 20Y0043 100.0% 98.8%
ARVQAVLRRTQFANKVTNKNKLYKNIEIDTDTHSVYIHSENKKILLNLTLTEYKIISFMIDQPHKVFTRGELMNHCMNDSDALERTVDSHVSKLRKKLEEQGIFQMLINVRGVGYRLDNP

43 20Y0040 100.0% 98.8%
ARVQAVLRRTQFANKVTNKNKLYKNIEIDTDTHSVYIHSENKKILLNLTLTEYKIISFMIDQPHKVFTRGELMNHCMNDSDALERTVDSHVSKLRKKLEEQGIFQMLINVRGVGYRLDNP

44 20Y0045 100.0% 98.0%
ARVQAVLRRTQFANKATNKNKLYKNIEIDNDTHSVYILSENKKILLNLTLTEYKIISFMIDQPHKVFTRGELMNQCMNDSDALERTVDSHVSKLRKKLEEQGIFQMLINVRGVGYRLDNP

45 20Y0046 100.0% 98.0%
ARVQAVLRRTQFANKATNKNKLYKNIEIDNDTHSVYILSENKKILLNLTLTEYKIISFMIDQPHKVFTRGELMNQCMNDSDALERTVDSHVSKLRKKLEEQGIFQMLINVRGVGYRLDNP

46 20Y0048 100.0% 98.8%
ARVQAVLRRTQFANKVTNKNKLYKNIEIDTDTHSVYIHSENKKILLNLTLTEYKIISFMIDQPHKVFTRGELMNHCMNDSDALERTVDSHVSKLRKKLEEQGIFQMLINVRGVGYRLDNP

47 20Y0036 100.0% 98.8%

ARVQAVLRRTQFANKVTNKNKLYKNIEIDTDTHSVYIHSENKKILLNLTLTEYKIISFMIDQPHKVFTRGELMNHCMNDSDALERTVDSHVSKLRKKLEEQGIFQMLINVRGVGYRLDNP

		cov	pid	241 247
				+-----+
1	ATCC19606	100.0%	100.0%	PAVKDDA
2	20Y0020	100.0%	98.8%	LAVKDDA
3	20Y0021	100.0%	98.0%	LAVKDDA
4	20Y0023	100.0%	98.8%	LAVKDDA
5	20Y0025	100.0%	98.8%	LAVKDDA
6	20Y0024	100.0%	98.8%	LAVKDDA
7	20Y0022	100.0%	98.0%	LAVKDDA
8	20Y0060	100.0%	98.8%	LAVKDDA
9	20Y0026	100.0%	98.8%	LAVKDDA
10	20Y0032	100.0%	98.8%	LAVKDDA
11	20Y0033	100.0%	98.8%	LAVKDDA
12	20Y0035	100.0%	98.0%	LAVKDDA
13	20Y0037	100.0%	98.8%	LAVKDDA
14	20Y0038	100.0%	98.8%	LAVKDDA
15	20Y0059	100.0%	98.8%	LAVKDDA
16	20Y0039	100.0%	98.8%	LAVKDDA
17	20Y0066	100.0%	98.8%	LAVKDDA
18	20Y0078	100.0%	98.8%	LAIKDDA
19	20Y0054	100.0%	98.8%	LAVKDDA
20	20Y0055	100.0%	98.8%	LAVKDDA
21	20Y0056	100.0%	98.8%	LAVKDDA
22	20Y0058	100.0%	98.0%	LAVKDDA
23	20Y0063	100.0%	98.8%	LAVKDDA
24	20Y0067	100.0%	98.8%	LAVKDDA
25	20Y0076	100.0%	98.0%	LAVKDDA
26	20Y0070	100.0%	98.8%	LAVKDDA
27	20Y0077	100.0%	97.2%	LAIKDDA
28	20Y0079	100.0%	98.8%	LAIKDDA
29	20Y0081	100.0%	98.0%	LAVKDDA
30	20Y0057	100.0%	99.6%	LAVKDDA
31	20Y0028	100.0%	98.8%	LAVKDDA
32	20Y0041	100.0%	98.8%	LAVKDDA
33	20Y0031	100.0%	98.8%	LAVKDDA
34	20Y0030	100.0%	98.8%	LAVKDDA
35	20Y0042	100.0%	98.8%	LAVKDDA
36	20Y0068	100.0%	98.8%	LAVKDDA
37	20Y0062	100.0%	98.8%	LAVKDDA
38	20Y0075	100.0%	98.8%	LAVKDDA
39	20Y0080	100.0%	99.6%	LAVKDDA
40	20Y0082	100.0%	98.0%	LAVKDDA
41	20Y0083	100.0%	98.8%	LAIKDDA
42	20Y0043	100.0%	98.8%	LAVKDDA
43	20Y0040	100.0%	98.8%	LAVKDDA
44	20Y0045	100.0%	98.0%	LAVKDDA
45	20Y0046	100.0%	98.0%	LAVKDDA
46	20Y0048	100.0%	98.8%	LAVKDDA
47	20Y0036	100.0%	98.8%	LAVKDDA

Figure S4: Multiple sequence alignment of the predicted amino acid sequences of AdeR in all isolates compared to that of *A. baumannii* ATCC 19606. The alignment was visualized using MView version 1.63. cov, coverage; pid, percent identity.

```
          cov    pid    1       10       20       30       40       50       60       70       80       90      100      110      120
                        +---------+---------+---------+---------+---------+---------+---------+---------+---------+---------+---------+---------+
```

1 ATCC19606 100.0% 100.0%
MKSKLGISKQLFIALTIVNLSVTLFSVVLGYVIYNYAIEKGWISLSSFQQEDWTSFHFVDWIWLATVIFCGCIISLVIGMRLAKRFIVPINFLAEAAKKISHGDLSARAYDNRIHSAEMS
2 20Y0023 100.0% 97.5%
MKSKLGISKQLFIALTIVNLSVTLFSVVLGYVIYNYAIEKGWISLSSFQQEDWTSFHFVDWIWLATVIFCGCIISLVIGMRLAKRFIVPINFLAEAAKKISHGDLSARAYDNRIHSAEMS
3 20Y0020 100.0% 97.5%
MKSKLGISKQLFIALTIVNLSVTLFSVVLGYVIYNYAIEKGWISLSSFQQEDWTSFHFVDWIWLATVIFCGCIISLVIGMRLAKRFIVPINFLAEAAKKISHGDLSARAYDNRIHSAEMS
4 20Y0021 100.0% 97.8%
MKSKLGISKQLFIALTIVNLSVTLFSVVLGYVIYNYAIEKGWISLSSFQQEDWTSFHFVDWIWLATVIFCGCIISLVIGMRLAKRFIVPINFLAEAAKKISHGDLSARAYDNRIHSAEMS
5 20Y0024 100.0% 97.5%
MKSKLGISKQLFIALTIVNLSVTLFSVVLGYVIYNYAIEKGWISLSSFQQEDWTSFHFVDWIWLATVIFCGCIISLVIGMRLAKRFIVPINFLAEAAKKISHGDLSARAYDNRIHSAEMS
6 20Y0022 100.0% 97.8%
MKSKLGISKQLFIALTIVNLSVTLFSVVLGYVIYNYAIEKGWISLSSFQQEDWTSFHFVDWIWLATVIFCGCIISLVIGMRLAKRFIVPINFLAEAAKKISHGDLSARAYDNRIHSAEMS
7 20Y0025 100.0% 97.5%
MKSKLGISKQLFIALTIVNLSVTLFSVVLGYVIYNYAIEKGWISLSSFQQEDWTSFHFVDWIWLATVIFCGCIISLVIGMRLAKRFIVPINFLAEAAKKISHGDLSARAYDNRIHSAEMS
8 20Y0026 100.0% 97.5%
MKSKLGISKQLFIALTIVNLSVTLFSVVLGYVIYNYAIEKGWISLSSFQQEDWTSFHFVDWIWLATVIFCGCIISLVIGMRLAKRFIVPINFLAEAAKKISHGDLSARAYDNRIHSAEMS
9 20Y0032 100.0% 97.5%
MKSKLGISKQLFIALTIVNLSVTLFSVVLGYVIYNYAIEKGWISLSSFQQEDWTSFHFVDWIWLATVIFCGCIISLVIGMRLAKRFIVPINFLAEAAKKISHGDLSARAYDNRIHSAEMS
10 20Y0033 100.0% 97.5%
MKSKLGISKQLFIALTIVNLSVTLFSVVLGYVIYNYAIEKGWISLSSFQQEDWTSFHFVDWIWLATVIFCGCIISLVIGMRLAKRFIVPINFLAEAAKKISHGDLSARAYDNRIHSAEMS
11 20Y0060 100.0% 97.5%
MKSKLGISKQLFIALTIVNLSVTLFSVVLGYVIYNYAIEKGWISLSSFQQEDWTSFHFVDWIWLATVIFCGCIISLVIGMRLAKRFIVPINFLAEAAKKISHGDLSARAYDNRIHSAEMS
12 20Y0035 100.0% 97.8%
MKSKLGISKQLFIALTIVNLSVTLFSVVLGYVIYNYAIEKGWISLSSFQQEDWTSFHFVDWIWLATVIFCGCIISLVIGMRLAKRFIVPINFLAEAAKKISHGDLSARAYDNRIHSAEMS
13 20Y0037 100.0% 97.5%
MKSKLGISKQLFIALTIVNLSVTLFSVVLGYVIYNYAIEKGWISLSSFQQEDWTSFHFVDWIWLATVIFCGCIISLVIGMRLAKRFIVPINFLAEAAKKISHGDLSARAYDNRIHSAEMS
14 20Y0059 100.0% 97.5%
MKSKLGISKQLFIALTIVNLSVTLFSVVLGYVIYNYAIEKGWISLSSFQQEDWTSFHFVDWIWLATVIFCGCIISLVIGMRLAKRFIVPINFLAEAAKKISHGDLSARAYDNRIHSAEMS
15 20Y0039 100.0% 97.5%
MKSKLGISKQLFIALTIVNLSVTLFSVVLGYVIYNYAIEKGWISLSSFQQEDWTSFHFVDWIWLATVIFCGCIISLVIGMRLAKRFIVPINFLAEAAKKISHGDLSARAYDNRIHSAEMS
16 20Y0038 100.0% 97.5%
MKSKLGISKQLFIALTIVNLSVTLFSVVLGYVIYNYAIEKGWISLSSFQQEDWTSFHFVDWIWLATVIFCGCIISLVIGMRLAKRFIVPINFLAEAAKKISHGDLSARAYDNRIHSAEMS
17 20Y0054 100.0% 97.5%
MKSKLGISKQLFIALTIVNLSVTLFSVVLGYVIYNYAIEKGWISLSSFQQEDWTSFHFVDWIWLATVIFCGCIISLVIGMRLAKRFIVPINFLAEAAKKISHGDLSARAYDNRIHSAEMS
18 20Y0066 100.0% 97.5%
MKSKLGISKQLFIALTIVNLSVTLFSVVLGYVIYNYAIEKGWISLSSFQQEDWTSFHFVDWIWLATVIFCGCIISLVIGMRLAKRFIVPINFLAEAAKKISHGDLSARAYDNRIHSAEMS
19 20Y0055 100.0% 97.5%
MKSKLGISKQLFIALTIVNLSVTLFSVVLGYVIYNYAIEKGWISLSSFQQEDWTSFHFVDWIWLATVIFCGCIISLVIGMRLAKRFIVPINFLAEAAKKISHGDLSARAYDNRIHSAEMS
20 20Y0078 100.0% 97.8%
MKSKLGISKQLFIALTIVNLSVTLFSVVLGYVIYNYAIEKGWISLSSFQQEDWTSFHFVDWIWLATVIFCGCIISLVIGMRLAKRFIVPINFLAEAAKKISHGDLSARAYDNRIHSAEMS
21 20Y0056 100.0% 97.5%
MKSKLGISKQLFIALTIVNLSVTLFSVVLGYVIYNYAIEKGWISLSSFQQEDWTSFHFVDWIWLATVIFCGCIISLVIGMRLAKRFIVPINFLAEAAKKISHGDLSARAYDNRIHSAEMS
22 20Y0058 100.0% 97.5%
MKSKLGISKQLFIALTIVNLSVTLFSVVLGYVIYNYAIEKGWISLSSFQQEDWTSFHFVDWIWLATVIFCGCIISLVIGMRLAKRFIVPINFLAEAAKKISHGDLSARAYDNRIHSAEMS
23 20Y0063 100.0% 97.5%
MKSKLGISKQLFIALTIVNLSVTLFSVVLGYVIYNYAIEKGWISLSSFQQEDWTSFHFVDWIWLATVIFCGCIISLVIGMRLAKRFIVPINFLAEAAKKISHGDLSARAYDNRIHSAEMS
24 20Y0057 100.0% 98.0%
MKSKLGISKQLFIALTIVNLSVTLFSVVLGYVIYNYAIEKGWISLSSFQQEDWTSFHFVDWIWLATVIFCGCIISLVIGMRLAKRFIVPINFLAEAAKKISHGDLSARAYDNRIHSAEMS
25 20Y0067 100.0% 97.5%
MKSKLGISKQLFIALTIVNLSVTLFSVVLGYVIYNYAIEKGWISLSSFQQEDWTSFHFVDWIWLATVIFCGCIISLVIGMRLAKRFIVPINFLAEAAKKISHGDLSARAYDNRIHSAEMS
26 20Y0076 100.0% 97.5%
MKSKLGISKQLFIALTIVNLSVTLFSVVLGYVIYNYAIEKGWISLSSFQQEDWTSFHFVDWIWLATVIFCGCIISLVIGMRLAKRFIVPINFLAEAAKKISHGDLSARAYDNRIHSAEMS
27 20Y0070 100.0% 97.5%
MKSKLGISKQLFIALTIVNLSVTLFSVVLGYVIYNYAIEKGWISLSSFQQEDWTSFHFVDWIWLATVIFCGCIISLVIGMRLAKRFIVPINFLAEAAKKISHGDLSARAYDNRIHSAEMS
28 20Y0079 100.0% 97.8%
MKSKLGISKQLFIALTIVNLSVTLFSVVLGYVIYNYAIEKGWISLSSFQQEDWTSFHFVDWIWLATVIFCGCIISLVIGMRLAKRFIVPINFLAEAAKKISHGDLSARAYDNRIHSAEMS
29 20Y0081 100.0% 97.5%
MKSKLGISKQLFIALTIVNLSVTLFSVVLGYVIYNYAIEKGWISLSSFQQEDWTSFHFVDWIWLATVIFCGCIISLVIGMRLAKRFIVPINFLAEAAKKISHGDLSARAYDNRIHSAEMS
30 20Y0028 100.0% 97.5%
MKSKLGISKQLFIALTIVNLSVTLFSVVLGYVIYNYAIEKGWISLSSFQQEDWTSFHFVDWIWLATVIFCGCIISLVIGMRLAKRFIVPINFLAEAAKKISHGDLSARAYDNRIHSAEMS
31 20Y0030 100.0% 97.5%
MKSKLGISKQLFIALTIVNLSVTLFSVVLGYVIYNYAIEKGWISLSSFQQEDWTSFHFVDWIWLATVIFCGCIISLVIGMRLAKRFIVPINFLAEAAKKISHGDLSARAYDNRIHSAEMS
32 20Y0031 100.0% 97.5%
MKSKLGISKQLFIALTIVNLSVTLFSVVLGYVIYNYAIEKGWISLSSFQQEDWTSFHFVDWIWLATVIFCGCIISLVIGMRLAKRFIVPINFLAEAAKKISHGDLSARAYDNRIHSAEMS
33 20Y0042 100.0% 97.5%
MKSKLGISKQLFIALTIVNLSVTLFSVVLGYVIYNYAIEKGWISLSSFQQEDWTSFHFVDWIWLATVIFCGCIISLVIGMRLAKRFIVPINFLAEAAKKISHGDLSARAYDNRIHSAEMS
34 20Y0041 100.0% 97.5%
MKSKLGISKQLFIALTIVNLSVTLFSVVLGYVIYNYAIEKGWISLSSFQQEDWTSFHFVDWIWLATVIFCGCIISLVIGMRLAKRFIVPINFLAEAAKKISHGDLSARAYDNRIHSAEMS
35 20Y0077 100.0% 95.8%
MKSKLGISKQLFIALTIVNLSVTLFSVVLGYVIYNYAIEKGWISLSSFQQEDWTSFHFVDWIWLATVIFCGCIISLVIGMRLAKRFIVPINFLAEAAKKISHGDLSARAYDNRIHSAEMS
36 20Y0062 100.0% 97.5%
MKSKLGISKQLFIALTIVNLSVTLFSVVLGYVIYNYAIEKGWISLSSFQQEDWTSFHFVDWIWLATVIFCGCIISLVIGMRLAKRFIVPINFLAEAAKKISHGDLSARAYDNRIHSAEMS
37 20Y0080 100.0% 97.8%
MKSKLGISKQLFIALTIVNLSVTLFSVVLGYVIYNYAIEKGWISLSSFQQEDWTSFHFVDWIWLATVIFCGCIISLVIGMRLAKRFIVPINFLAEAAKKISHGDLSARAYDNRIHSAEMS
38 20Y0075 100.0% 97.5%
MKSKLGISKQLFIALTIVNLSVTLFSVVLGYVIYNYAIEKGWISLSSFQQEDWTSFHFVDWIWLATVIFCGCIISLVIGMRLAKRFIVPINFLAEAAKKISHGDLSARAYDNRIHSAEMS
39 20Y0068 100.0% 97.5%
MKSKLGISKQLFIALTIVNLSVTLFSVVLGYVIYNYAIEKGWISLSSFQQEDWTSFHFVDWIWLATVIFCGCIISLVIGMRLAKRFIVPINFLAEAAKKISHGDLSARAYDNRIHSAEMS
40 20Y0082 100.0% 97.5%
MKSKLGISKQLFIALTIVNLSVTLFSVVLGYVIYNYAIEKGWISLSSFQQEDWTSFHFVDWIWLATVIFCGCIISLVIGMRLAKRFIVPINFLAEAAKKISHGDLSARAYDNRIHSAEMS
41 20Y0043 100.0% 97.5%
MKSKLGISKQLFIALTIVNLSVTLFSVVLGYVIYNYAIEKGWISLSSFQQEDWTSFHFVDWIWLATVIFCGCIISLVIGMRLAKRFIVPINFLAEAAKKISHGDLSARAYDNRIHSAEMS
42 20Y0040 100.0% 97.5%
MKSKLGISKQLFIALTIVNLSVTLFSVVLGYVIYNYAIEKGWISLSSFQQEDWTSFHFVDWIWLATVIFCGCIISLVIGMRLAKRFIVPINFLAEAAKKISHGDLSARAYDNRIHSAEMS
43 20Y0083 100.0% 97.8%
MKSKLGISKQLFIALTIVNLSVTLFSVVLGYVIYNYAIEKGWISLSSFQQEDWTSFHFVDWIWLATVIFCGCIISLVIGMRLAKRFIVPINFLAEAAKKISHGDLSARAYDNRIHSAEMS
44 20Y0045 100.0% 97.8%
MKSKLGISKQLFIALTIVNLSVTLFSVVLGYVIYNYAIEKGWISLSSFQQEDWTSFHFVDWIWLATVIFCGCIISLVIGMRLAKRFIVPINFLAEAAKKISHGDLSARAYDNRIHSAEMS
45 20Y0048 100.0% 97.5%
MKSKLGISKQLFIALTIVNLSVTLFSVVLGYVIYNYAIEKGWISLSSFQQEDWTSFHFVDWIWLATVIFCGCIISLVIGMRLAKRFIVPINFLAEAAKKISHGDLSARAYDNRIHSAEMS
46 20Y0046 100.0% 97.8%
MKSKLGISKQLFIALTIVNLSVTLFSVVLGYVIYNYAIEKGWISLSSFQQEDWTSFHFVDWIWLATVIFCGCIISLVIGMRLAKRFIVPINFLAEAAKKISHGDLSARAYDNRIHSAEMS
47 20Y0036 100.0% 97.5%
MKSKLGISKQLFIALTIVNLSVTLFSVVLGYVIYNYAIEKGWISLSSFQQEDWTSFHFVDWIWLATVIFCGCIISLVIGMRLAKRFIVPINFLAEAAKKISHGDLSARAYDNRIHSAEMS

```
             cov    pid    121   130   140   150   160   170   180   190   200   210   220   230   240
                           +--------+---------+---------+---------+---------+---------+---------+---------+---------+---------+---------+---------+
1 ATCC19606 100.0% 100.0%
ELLYNFNDMAQKLEVSVKNAQVWNAAIAHELRAPITILQGRLQGIIDGVFKPDEVLFKSLLNQVEGLSHLVEDLRTLSLVENQQLRLNYELFDLKAVVEKVLKAFEDRLDQAKLVPELDL
2 20Y0023   100.0%  97.5%
ELLYNFNDMAQKLEVSVKNAQVWNAAIAHELRTPITILQGRLQGIIDGVFKPDEVLFKSLLNQVEVLSHLVEDLRTLSLVENQQLRLNYELFDFKAVVEKVLKAFEDRLDQAKLVPELDL
3 20Y0020   100.0%  97.5%
ELLYNFNDMAQKLEVSVKNAQVWNAAIAHELRTPITILQGRLQGIIDGVFKPDEVLFKSLLNQVEVLSHLVEDLRTLSLVENQQLRLNYELFDFKAVVEKVLKAFEDRLDQAKLVPELDL
4 20Y0021   100.0%  97.8%
ELLYNFNDMAQKLEVSVKNAQVWNAAIAHELRTPITILQGRLQGIIDGVFKPDEVLFKSLLNQVEGLSHLVEDLRTLSLVENQQLRLNYELFDFKAVVEKVLKAFEHRLDQAKLVPELDL
5 20Y0024   100.0%  97.5%
ELLYNFNDMAQKLEVSVKNAQVWNAAIAHELRTPITILQGRLQGIIDGVFKPDEVLFKSLLNQVEVLSHLVEDLRTLSLVENQQLRLNYELFDFKAVVEKVLKAFEDRLDQAKLVPELDL
6 20Y0022   100.0%  97.8%
ELLYNFNDMAQKLEVSVKNAQVWNAAIAHELRTPITILQGRLQGIIDGVFKPDEVLFKSLLNQVEGLSHLVEDLRTLSLVENQQLRLNYELFDFKAVVEKVLKAFEHRLDQAKLVPELDL
7 20Y0025   100.0%  97.5%
ELLYNFNDMAQKLEVSVKNAQVWNAAIAHELRTPITILQGRLQGIIDGVFKPDEVLFKSLLNQVEVLSHLVEDLRTLSLVENQQLRLNYELFDFKAVVEKVLKAFEDRLDQAKLVPELDL
8 20Y0026   100.0%  97.5%
ELLYNFNDMAQKLEVSVKNAQVWNAAIAHELRTPITILQGRLQGIIDGVFKPDEVLFKSLLNQVEVLSHLVEDLRTLSLVENQQLRLNYELFDFKAVVEKVLKAFEDRLDQAKLVPELDL
9 20Y0032   100.0%  97.5%
ELLYNFNDMAQKLEVSVKNAQVWNAAIAHELRTPITILQGRLQGIIDGVFKPDEVLFKSLLNQVEVLSHLVEDLRTLSLVENQQLRLNYELFDFKAVVEKVLKAFEDRLDQAKLVPELDL
10 20Y0033   100.0%  97.5%
ELLYNFNDMAQKLEVSVKNAQVWNAAIAHELRTPITILQGRLQGIIDGVFKPDEVLFKSLLNQVEVLSHLVEDLRTLSLVENQQLRLNYELFDFKAVVEKVLKAFEDRLDQAKLVPELDL
11 20Y0060   100.0%  97.5%
ELLYNFNDMAQKLEVSVKNAQVWNAAIAHELRTPITILQGRLQGIIDGVFKPDEVLFKSLLNQVEVLSHLVEDLRTLSLVENQQLRLNYELFDFKAVVEKVLKAFEDRLDQAKLVPELDL
12 20Y0035   100.0%  97.8%
ELLYNFNDMAQKLEVSVKNAQVWNAAIAHELRTPITILQGRLQGIIDGVFKPDEVLFKSLLNQVEGLSHLVEDLRTLSLVENQQLRLNYELFDFKAVVEKVLKAFEHRLDQAKLVPELDL
13 20Y0037   100.0%  97.5%
ELLYNFNDMAQKLEVSVKNAQVWNAAIAHELRTPITILQGRLQGIIDGVFKPDEVLFKSLLNQVEVLSHLVEDLRTLSLVENQQLRLNYELFDFKAVVEKVLKAFEDRLDQAKLVPELDL
14 20Y0059   100.0%  97.5%
ELLYNFNDMAQKLEVSVKNAQVWNAAIAHELRTPITILQGRLQGIIDGVFKPDEVLFKSLLNQVEVLSHLVEDLRTLSLVENQQLRLNYELFDFKAVVEKVLKAFEDRLDQAKLVPELDL
15 20Y0039   100.0%  97.5%
ELLYNFNDMAQKLEVSVKNAQVWNAAIAHELRTPITILQGRLQGIIDGVFKPDEVLFKSLLNQVEVLSHLVEDLRTLSLVENQQLRLNYELFDFKAVVEKVLKAFEDRLDQAKLVPELDL
16 20Y0038   100.0%  97.5%
ELLYNFNDMAQKLEVSVKNAQVWNAAIAHELRTPITILQGRLQGIIDGVFKPDEVLFKSLLNQVEVLSHLVEDLRTLSLVENQQLRLNYELFDFKAVVEKVLKAFEDRLDQAKLVPELDL
17 20Y0054   100.0%  97.5%
ELLYNFNDMAQKLEVSVKNAQVWNAAIAHELRTPITILQGRLQGIIDGVFKPDEVLFKSLLNQVEVLSHLVEDLRTLSLVENQQLRLNYELFDFKAVVEKVLKAFEDRLDQAKLVPELDL
18 20Y0066   100.0%  97.5%
ELLYNFNDMAQKLEVSVKNAQVWNAAIAHELRTPITILQGRLQGIIDGVFKPDEVLFKSLLNQVEVLSHLVEDLRTLSLVENQQLRLNYELFDFKAVVEKVLKAFEDRLDQAKLVPELDL
19 20Y0055   100.0%  97.5%
ELLYNFNDMAQKLEVSVKNAQVWNAAIAHELRTPITILQGRLQGIIDGVFKPDEVLFKSLLNQVEVLSHLVEDLRTLSLVENQQLRLNYELFDFKAVVEKVLKAFEDRLDQAKLVPELDL
20 20Y0078   100.0%  97.8%
ELLYNFNDMAQKLEVSVKNAQVWNAAIAHELRTPITILQGRLQGIIDGVFKPDEVLFKSLLNQVEGLSHLVEDLRTLSLVENQQLRLNYELFDFKAVVEKVLKAFEDRLDQAKLVPELDL
21 20Y0056   100.0%  97.5%
ELLYNFNDMAQKLEVSVKNAQVWNAAIAHELRTPITILQGRLQGIIDGVFKPDEVLFKSLLNQVEVLSHLVEDLRTLSLVENQQLRLNYELFDFKAVVEKVLKAFEDRLDQAKLVPELDL
22 20Y0058   100.0%  97.5%
ELLYNFNDMAQKLEVSVKNAQVWNAAIAHELRTPITILQGRLQGIIDGVFKPDEVLFKSLLNQVEGLSHLVEDLRTFSLVENQQLRLNYELFDFKAVVEKVLKAFEHRLDQAKLVPELDL
23 20Y0063   100.0%  97.5%
ELLYNFNDMAQKLEVSVKNAQVWNAAIAHELRTPITILQGRLQGIIDGVFKPDEVLFKSLLNQVEVLSHLVEDLRTLSLVENQQLRLNYELFDFKAVVEKVLKAFEDRLDQAKLVPELDL
24 20Y0057   100.0%  98.0%
ELLYNFNDMAQKLEVSVKNAQVWNAAIAHELRTPITILQGRLQGIIDGVFKPDEVLFKSLLNQVEGLSHLVEDLRTLSLVENQQLRLNYELFDLKAVVEKVLKAFEDRLDQAKLVPELDL
25 20Y0067   100.0%  97.5%
ELLYNFNDMAQKLEVSVKNAQVWNAAIAHELRTPITILQGRLQGIIDGVFKPDEVLFKSLLNQVEVLSHLVEDLRTLSLVENQQLRLNYELFDFKAVVEKVLKAFEDRLDQAKLVPELDL
26 20Y0076   100.0%  97.5%
ELLYNFNDMAQKLEVSVKNAQVWNAAIAHELRTPITILQGRLQGIIDGVFKPDEVLFKSLLNQVEGLSHLVEDLRTFSLVENQQLRLNYELFDFKAVVEKVLKAFEHRLDQAKLVPELDL
27 20Y0070   100.0%  97.5%
ELLYNFNDMAQKLEVSVKNAQVWNAAIAHELRTPITILQGRLQGIIDGVFKPDEVLFKSLLNQVEVLSHLVEDLRTLSLVENQQLRLNYELFDFKAVVEKVLKAFEDRLDQAKLVPELDL
28 20Y0079   100.0%  97.8%
ELLYNFNDMAQKLEVSVKNAQVWNAAIAHELRTPITILQGRLQGIIDGVFKPDEVLFKSLLNQVEGLSHLVEDLRTLSLVENQQLRLNYELFDFKAVVEKVLKAFEDRLDQAKLVPELDL
29 20Y0081   100.0%  97.5%
ELLYNFNDMAQKLEVSVKNAQVWNAAIAHELRTPITILQGRLQGIIDGVFKPDEVLFKSLLNQVEGLSHLVEDLRTFSLVENQQLRLNYELFDFKAVVEKVLKAFEHRLDQAKLVPELDL
30 20Y0028   100.0%  97.5%
ELLYNFNDMAQKLEVSVKNAQVWNAAIAHELRTPITILQGRLQGIIDGVFKPDEVLFKSLLNQVEVLSHLVEDLRTLSLVENQQLRLNYELFDFKAVVEKVLKAFEDRLDQAKLVPELDL
31 20Y0030   100.0%  97.5%
ELLYNFNDMAQKLEVSVKNAQVWNAAIAHELRTPITILQGRLQGIIDGVFKPDEVLFKSLLNQVEVLSHLVEDLRTLSLVENQQLRLNYELFDFKAVVEKVLKAFEDRLDQAKLVPELDL
32 20Y0031   100.0%  97.5%
ELLYNFNDMAQKLEVSVKNAQVWNAAIAHELRTPITILQGRLQGIIDGVFKPDEVLFKSLLNQVEVLSHLVEDLRTLSLVENQQLRLNYELFDFKAVVEKVLKAFEDRLDQAKLVPELDL
33 20Y0042   100.0%  97.5%
ELLYNFNDMAQKLEVSVKNAQVWNAAIAHELRTPITILQGRLQGIIDGVFKPDEVLFKSLLNQVEVLSHLVEDLRTLSLVENQQLRLNYELFDFKAVVEKVLKAFEDRLDQAKLVPELDL
34 20Y0041   100.0%  97.5%
ELLYNFNDMAQKLEVSVKNAQVWNAAIAHELRTPITILQGRLQGIIDGVFKPDEVLFKSLLNQVEVLSHLVEDLRTLSLVENQQLRLNYELFDFKAVVEKVLKAFEDRLDQAKLVPELDL
35 20Y0077   100.0%  95.8%
ELLYNFNDMAQKLEVSVKNAQVWNAAIAHELRTPITILQGRLQGIIDGVFKPDEVLFKSLLNQVEGLSHLVEDLRTLSLVENQQLRLNYELFDFKAVVEKVLKAFEHRLDQAKLVPELDL
36 20Y0062   100.0%  97.5%
ELLYNFNDMAQKLEVSVKNAQVWNAAIAHELRTPITILQGRLQGIIDGVFKPDEVLFKSLLNQVEVLSHLVEDLRTLSLVENQQLRLNYELFDFKAVVEKVLKAFEDRLDQAKLVPELDL
37 20Y0080   100.0%  97.8%
ELLYNFNDMAQKLEVSVKNAQVWNAAIAHELRTPITILQGRLQGIIDGVFKPDEVLFKSLLNQVEGLSHLVEDLRTLSLVENQQLRLNYELFDLKAVVEKVLKAFEDRLDQAKLVPELDL
38 20Y0075   100.0%  97.5%
ELLYNFNDMAQKLEVSVKNAQVWNAAIAHELRTPITILQGRLQGIIDGVFKPDEVLFKSLLNQVEVLSHLVEDLRTLSLVENQQLRLNYELFDFKAVVEKVLKAFEDRLDQAKLVPELDL
39 20Y0068   100.0%  97.5%
ELLYNFNDMAQKLEVSVKNAQVWNAAIAHELRTPITILQGRLQGIIDGVFKPDEVLFKSLLNQVEVLSHLVEDLRTLSLVENQQLRLNYELFDFKAVVEKVLKAFEDRLDQAKLVPELDL
40 20Y0082   100.0%  97.5%
ELLYNFNDMAQKLEVSVKNAQVWNAAIAHELRTPITILQGRLQGIIDGVFKPDEVLFKSLLNQVEGLSHLVEDLRTFSLVENQQLRLNYELFDFKAVVEKVLKAFEHRLDQAKLVPELDL
41 20Y0043   100.0%  97.5%
ELLYNFNDMAQKLEVSVKNAQVWNAAIAHELRTPITILQGRLQGIIDGVFKPDEVLFKSLLNQVEVLSHLVEDLRTLSLVENQQLRLNYELFDFKAVVEKVLKAFEDRLDQAKLVPELDL
42 20Y0040   100.0%  97.5%
ELLYNFNDMAQKLEVSVKNAQVWNAAIAHELRTPITILQGRLQGIIDGVFKPDEVLFKSLLNQVEVLSHLVEDLRTLSLVENQQLRLNYELFDFKAVVEKVLKAFEDRLDQAKLVPELDL
43 20Y0083   100.0%  97.8%
ELLYNFNDMAQKLEVSVKNAQVWNAAIAHELRTPITILQGRLQGIIDGVFKPDEVLFKSLLNQVEGLSHLVEDLRTLSLVENQQLRLNYELFDFKAVVEKVLKAFEDRLDQAKLVPELDL
44 20Y0045   100.0%  97.8%
ELLYNFNDMAQKLEVSVKNAQVWNAAIAHELRTPITILQGRLQGIIDGVFKPDEVLFKSLLNQVEGLSHLVEDLRTLSLVENQQLRLNYELFDFKAVVEKVLKAFEHRLDQAKLVPELDL
45 20Y0048   100.0%  97.5%
ELLYNFNDMAQKLEVSVKNAQVWNAAIAHELRTPITILQGRLQGIIDGVFKPDEVLFKSLLNQVEVLSHLVEDLRTLSLVENQQLRLNYELFDFKAVVEKVLKAFEDRLDQAKLVPELDL
46 20Y0046   100.0%  97.8%
ELLYNFNDMAQKLEVSVKNAQVWNAAIAHELRTPITILQGRLQGIIDGVFKPDEVLFKSLLNQVEGLSHLVEDLRTLSLVENQQLRLNYELFDFKAVVEKVLKAFEHRLDQAKLVPELDL
```

47 20Y0036 100.0% 97.5%

ELLYNFNDMAQKLEVSVKNAQVWNAAIAHELRTPITILQGRLQGIIDGVFKPDEVLFKSLLNQVEVLSHLVEDLRTLSLVENQQLRLNYELFDFKAVVEKVLKAFEDRLDQAKLVPELDL

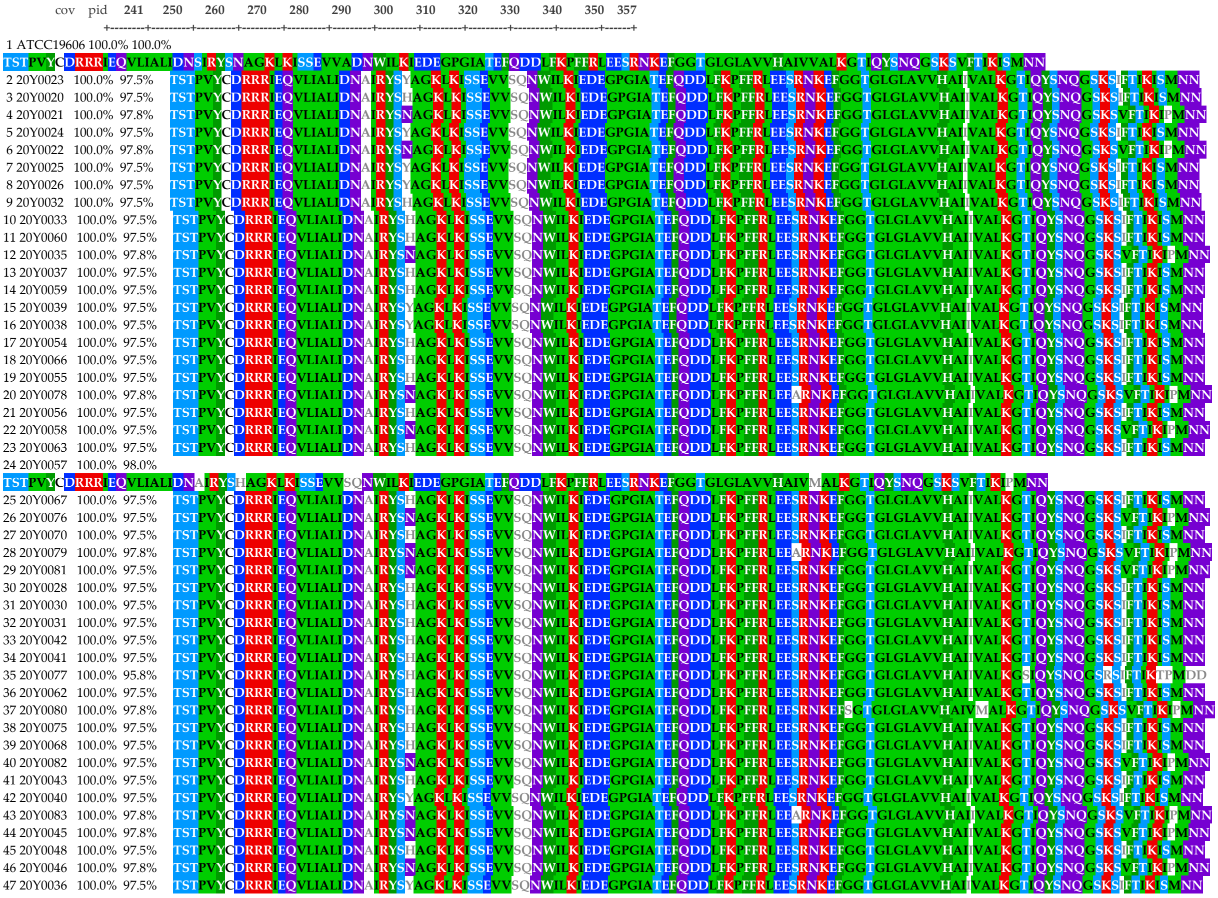

Figure S5: Multiple sequence alignment of the predicted amino acid sequences of AdeS in all isolates compared to that of *A. baumannii* ATCC 19606. The alignment was visualized using MView version 1.63. cov, coverage; pid, percent identity.

cov pid 1 10 20 30 40 50 60 70 80 90 100 110 120
+---------+---------+---------+---------+---------+---------+---------+---------+---------+---------+---------+---------+---------+

1 ATCC19606 100.0% 100.0%
MKHIMLVEDEVELAHLVRDYLEAAGFEVSMFHDGQDAYTSFQQRKPNLMILDLMVPRMDGLTICRKVREQSDLPIIMVTARTEEIDRVLGLNMGADDYVCKPFSPKELVARVQAVLRRLE

2 20Y0024 100.0% 100.0%
MKHIMLVEDEVELAHLVRDYLEAAGFEVSMFHDGQDAYTSFQQRKPNLMILDLMVPRMDGLTICRKVREQSDLPIIMVTARTEEIDRVLGLNMGADDYVCKPFSPKELVARVQAVLRRLE

3 20Y0056 100.0% 100.0%
MKHIMLVEDEVELAHLVRDYLEAAGFEVSMFHDGQDAYTSFQQRKPNLMILDLMVPRMDGLTICRKVREQSDLPIIMVTARTEEIDRVLGLNMGADDYVCKPFSPKELVARVQAVLRRLE

4 20Y0055 100.0% 100.0%
MKHIMLVEDEVELAHLVRDYLEAAGFEVSMFHDGQDAYTSFQQRKPNLMILDLMVPRMDGLTICRKVREQSDLPIIMVTARTEEIDRVLGLNMGADDYVCKPFSPKELVARVQAVLRRLE

5 20Y0063 100.0% 100.0%
MKHIMLVEDEVELAHLVRDYLEAAGFEVSMFHDGQDAYTSFQQRKPNLMILDLMVPRMDGLTICRKVREQSDLPIIMVTARTEEIDRVLGLNMGADDYVCKPFSPKELVARVQAVLRRLE

6 20Y0077 100.0% 99.6%
MKHIMLVEDEVELAHLVRDYLEAAGFEVSMFHDGQDAYTSFQQRKPNLMILDLMVPRMDGLTICRKVREQSDLPIIMVTARTEEIDRVLGLNMGADDYVCKPFSPKELVARVQAVLRRLE

7 20Y0037 100.0% 100.0%
MKHIMLVEDEVELAHLVRDYLEAAGFEVSMFHDGQDAYTSFQQRKPNLMILDLMVPRMDGLTICRKVREQSDLPIIMVTARTEEIDRVLGLNMGADDYVCKPFSPKELVARVQAVLRRLE

8 20Y0032 100.0% 100.0%
MKHIMLVEDEVELAHLVRDYLEAAGFEVSMFHDGQDAYTSFQQRKPNLMILDLMVPRMDGLTICRKVREQSDLPIIMVTARTEEIDRVLGLNMGADDYVCKPFSPKELVARVQAVLRRLE

9 20Y0059 100.0% 100.0%
MKHIMLVEDEVELAHLVRDYLEAAGFEVSMFHDGQDAYTSFQQRKPNLMILDLMVPRMDGLTICRKVREQSDLPIIMVTARTEEIDRVLGLNMGADDYVCKPFSPKELVARVQAVLRRLE

10 20Y0081 100.0% 100.0%
MKHIMLVEDEVELAHLVRDYLEAAGFEVSMFHDGQDAYTSFQQRKPNLMILDLMVPRMDGLTICRKVREQSDLPIIMVTARTEEIDRVLGLNMGADDYVCKPFSPKELVARVQAVLRRLE

11 20Y0076 100.0% 100.0%
MKHIMLVEDEVELAHLVRDYLEAAGFEVSMFHDGQDAYTSFQQRKPNLMILDLMVPRMDGLTICRKVREQSDLPIIMVTARTEEIDRVLGLNMGADDYVCKPFSPKELVARVQAVLRRLE

12 20Y0036 100.0% 100.0%
MKHIMLVEDEVELAHLVRDYLEAAGFEVSMFHDGQDAYTSFQQRKPNLMILDLMVPRMDGLTICRKVREQSDLPIIMVTARTEEIDRVLGLNMGADDYVCKPFSPKELVARVQAVLRRLE

13 20Y0030 100.0% 100.0%
MKHIMLVEDEVELAHLVRDYLEAAGFEVSMFHDGQDAYTSFQQRKPNLMILDLMVPRMDGLTICRKVREQSDLPIIMVTARTEEIDRVLGLNMGADDYVCKPFSPKELVARVQAVLRRLE

14 20Y0033 100.0% 100.0%
MKHIMLVEDEVELAHLVRDYLEAAGFEVSMFHDGQDAYTSFQQRKPNLMILDLMVPRMDGLTICRKVREQSDLPIIMVTARTEEIDRVLGLNMGADDYVCKPFSPKELVARVQAVLRRLE

15 20Y0068 100.0% 100.0%
MKHIMLVEDEVELAHLVRDYLEAAGFEVSMFHDGQDAYTSFQQRKPNLMILDLMVPRMDGLTICRKVREQSDLPIIMVTARTEEIDRVLGLNMGADDYVCKPFSPKELVARVQAVLRRLE

16 20Y0042 100.0% 100.0%
MKHIMLVEDEVELAHLVRDYLEAAGFEVSMFHDGQDAYTSFQQRKPNLMILDLMVPRMDGLTICRKVREQSDLPIIMVTARTEEIDRVLGLNMGADDYVCKPFSPKELVARVQAVLRRLE

17 20Y0062 100.0% 100.0%
MKHIMLVEDEVELAHLVRDYLEAAGFEVSMFHDGQDAYTSFQQRKPNLMILDLMVPRMDGLTICRKVREQSDLPIIMVTARTEEIDRVLGLNMGADDYVCKPFSPKELVARVQAVLRRLE

18 20Y0066 100.0% 100.0%
MKHIMLVEDEVELAHLVRDYLEAAGFEVSMFHDGQDAYTSFQQRKPNLMILDLMVPRMDGLTICRKVREQSDLPIIMVTARTEEIDRVLGLNMGADDYVCKPFSPKELVARVQAVLRRLE

19 20Y0045 100.0% 100.0%
MKHIMLVEDEVELAHLVRDYLEAAGFEVSMFHDGQDAYTSFQQRKPNLMILDLMVPRMDGLTICRKVREQSDLPIIMVTARTEEIDRVLGLNMGADDYVCKPFSPKELVARVQAVLRRLE

20 20Y0020 100.0% 100.0%
MKHIMLVEDEVELAHLVRDYLEAAGFEVSMFHDGQDAYTSFQQRKPNLMILDLMVPRMDGLTICRKVREQSDLPIIMVTARTEEIDRVLGLNMGADDYVCKPFSPKELVARVQAVLRRLE

21 20Y0070 100.0% 100.0%
MKHIMLVEDEVELAHLVRDYLEAAGFEVSMFHDGQDAYTSFQQRKPNLMILDLMVPRMDGLTICRKVREQSDLPIIMVTARTEEIDRVLGLNMGADDYVCKPFSPKELVARVQAVLRRLE

22 20Y0041 100.0% 100.0%
MKHIMLVEDEVELAHLVRDYLEAAGFEVSMFHDGQDAYTSFQQRKPNLMILDLMVPRMDGLTICRKVREQSDLPIIMVTARTEEIDRVLGLNMGADDYVCKPFSPKELVARVQAVLRRLE

23 20Y0038 100.0% 100.0%
MKHIMLVEDEVELAHLVRDYLEAAGFEVSMFHDGQDAYTSFQQRKPNLMILDLMVPRMDGLTICRKVREQSDLPIIMVTARTEEIDRVLGLNMGADDYVCKPFSPKELVARVQAVLRRLE

24 20Y0025 100.0% 100.0%
MKHIMLVEDEVELAHLVRDYLEAAGFEVSMFHDGQDAYTSFQQRKPNLMILDLMVPRMDGLTICRKVREQSDLPIIMVTARTEEIDRVLGLNMGADDYVCKPFSPKELVARVQAVLRRLE

25 20Y0022 100.0% 100.0%
MKHIMLVEDEVELAHLVRDYLEAAGFEVSMFHDGQDAYTSFQQRKPNLMILDLMVPRMDGLTICRKVREQSDLPIIMVTARTEEIDRVLGLNMGADDYVCKPFSPKELVARVQAVLRRLE

26 20Y0079 100.0% 100.0%
MKHIMLVEDEVELAHLVRDYLEAAGFEVSMFHDGQDAYTSFQQRKPNLMILDLMVPRMDGLTICRKVREQSDLPIIMVTARTEEIDRVLGLNMGADDYVCKPFSPKELVARVQAVLRRLE

27 20Y0043 100.0% 100.0%
MKHIMLVEDEVELAHLVRDYLEAAGFEVSMFHDGQDAYTSFQQRKPNLMILDLMVPRMDGLTICRKVREQSDLPIIMVTARTEEIDRVLGLNMGADDYVCKPFSPKELVARVQAVLRRLE

28 20Y0040 100.0% 100.0%
MKHIMLVEDEVELAHLVRDYLEAAGFEVSMFHDGQDAYTSFQQRKPNLMILDLMVPRMDGLTICRKVREQSDLPIIMVTARTEEIDRVLGLNMGADDYVCKPFSPKELVARVQAVLRRLE

29 20Y0054 100.0% 100.0%
MKHIMLVEDEVELAHLVRDYLEAAGFEVSMFHDGQDAYTSFQQRKPNLMILDLMVPRMDGLTICRKVREQSDLPIIMVTARTEEIDRVLGLNMGADDYVCKPFSPKELVARVQAVLRRLE

30 20Y0057 100.0% 100.0%
MKHIMLVEDEVELAHLVRDYLEAAGFEVSMFHDGQDAYTSFQQRKPNLMILDLMVPRMDGLTICRKVREQSDLPIIMVTARTEEIDRVLGLNMGADDYVCKPFSPKELVARVQAVLRRLE

31 20Y0028 100.0% 100.0%
MKHIMLVEDEVELAHLVRDYLEAAGFEVSMFHDGQDAYTSFQQRKPNLMILDLMVPRMDGLTICRKVREQSDLPIIMVTARTEEIDRVLGLNMGADDYVCKPFSPKELVARVQAVLRRLE

32 20Y0080 100.0% 100.0%
MKHIMLVEDEVELAHLVRDYLEAAGFEVSMFHDGQDAYTSFQQRKPNLMILDLMVPRMDGLTICRKVREQSDLPIIMVTARTEEIDRVLGLNMGADDYVCKPFSPKELVARVQAVLRRLE

33 20Y0078 100.0% 100.0%
MKHIMLVEDEVELAHLVRDYLEAAGFEVSMFHDGQDAYTSFQQRKPNLMILDLMVPRMDGLTICRKVREQSDLPIIMVTARTEEIDRVLGLNMGADDYVCKPFSPKELVARVQAVLRRLE

34 20Y0023 100.0% 100.0%
MKHIMLVEDEVELAHLVRDYLEAAGFEVSMFHDGQDAYTSFQQRKPNLMILDLMVPRMDGLTICRKVREQSDLPIIMVTARTEEIDRVLGLNMGADDYVCKPFSPKELVARVQAVLRRLE

35 20Y0058 100.0% 100.0%
MKHIMLVEDEVELAHLVRDYLEAAGFEVSMFHDGQDAYTSFQQRKPNLMILDLMVPRMDGLTICRKVREQSDLPIIMVTARTEEIDRVLGLNMGADDYVCKPFSPKELVARVQAVLRRLE

36 20Y0075 100.0% 100.0%
MKHIMLVEDEVELAHLVRDYLEAAGFEVSMFHDGQDAYTSFQQRKPNLMILDLMVPRMDGLTICRKVREQSDLPIIMVTARTEEIDRVLGLNMGADDYVCKPFSPKELVARVQAVLRRLE

37 20Y0083 100.0% 100.0%
MKHIMLVEDEVELAHLVRDYLEAAGFEVSMFHDGQDAYTSFQQRKPNLMILDLMVPRMDGLTICRKVREQSDLPIIMVTARTEEIDRVLGLNMGADDYVCKPFSPKELVARVQAVLRRLE

38 20Y0048 100.0% 100.0%
MKHIMLVEDEVELAHLVRDYLEAAGFEVSMFHDGQDAYTSFQQRKPNLMILDLMVPRMDGLTICRKVREQSDLPIIMVTARTEEIDRVLGLNMGADDYVCKPFSPKELVARVQAVLRRLE

39 20Y0035 100.0% 100.0%
MKHIMLVEDEVELAHLVRDYLEAAGFEVSMFHDGQDAYTSFQQRKPNLMILDLMVPRMDGLTICRKVREQSDLPIIMVTARTEEIDRVLGLNMGADDYVCKPFSPKELVARVQAVLRRLE

40 20Y0046 100.0% 100.0%
MKHIMLVEDEVELAHLVRDYLEAAGFEVSMFHDGQDAYTSFQQRKPNLMILDLMVPRMDGLTICRKVREQSDLPIIMVTARTEEIDRVLGLNMGADDYVCKPFSPKELVARVQAVLRRLE

41 20Y0031 100.0% 100.0%
MKHIMLVEDEVELAHLVRDYLEAAGFEVSMFHDGQDAYTSFQQRKPNLMILDLMVPRMDGLTICRKVREQSDLPIIMVTARTEEIDRVLGLNMGADDYVCKPFSPKELVARVQAVLRRLE

42 20Y0067 100.0% 100.0%
MKHIMLVEDEVELAHLVRDYLEAAGFEVSMFHDGQDAYTSFQQRKPNLMILDLMVPRMDGLTICRKVREQSDLPIIMVTARTEEIDRVLGLNMGADDYVCKPFSPKELVARVQAVLRRLE

43 20Y0026 100.0% 100.0%
MKHIMLVEDEVELAHLVRDYLEAAGFEVSMFHDGQDAYTSFQQRKPNLMILDLMVPRMDGLTICRKVREQSDLPIIMVTARTEEIDRVLGLNMGADDYVCKPFSPKELVARVQAVLRRLE

44 20Y0082 100.0% 100.0%
MKHIMLVEDEVELAHLVRDYLEAAGFEVSMFHDGQDAYTSFQQRKPNLMILDLMVPRMDGLTICRKVREQSDLPIIMVTARTEEIDRVLGLNMGADDYVCKPFSPKELVARVQAVLRRLE

45 20Y0060 100.0% 100.0%
MKHIMLVEDEVELAHLVRDYLEAAGFEVSMFHDGQDAYTSFQQRKPNLMILDLMVPRMDGLTICRKVREQSDLPIIMVTARTEEIDRVLGLNMGADDYVCKPFSPKELVARVQAVLRRLE

46 20Y0021 100.0% 100.0%
MKHIMLVEDEVELAHLVRDYLEAAGFEVSMFHDGQDAYTSFQQRKPNLMILDLMVPRMDGLTICRKVREQSDLPIIMVTARTEEIDRVLGLNMGADDYVCKPFSPKELVARVQAVLRRLE

47 20Y0039 100.0% 100.0%
MKHIMLVEDEVELAHLVRDYLEAAGFEVSMFHDGQDAYTSFQQRKPNLMILDLMVPRMDGLTICRKVREQSDLPIIMVTARTEEIDRVLGLNMGADDYVCKPFSPKELVARVQAVLRRLE

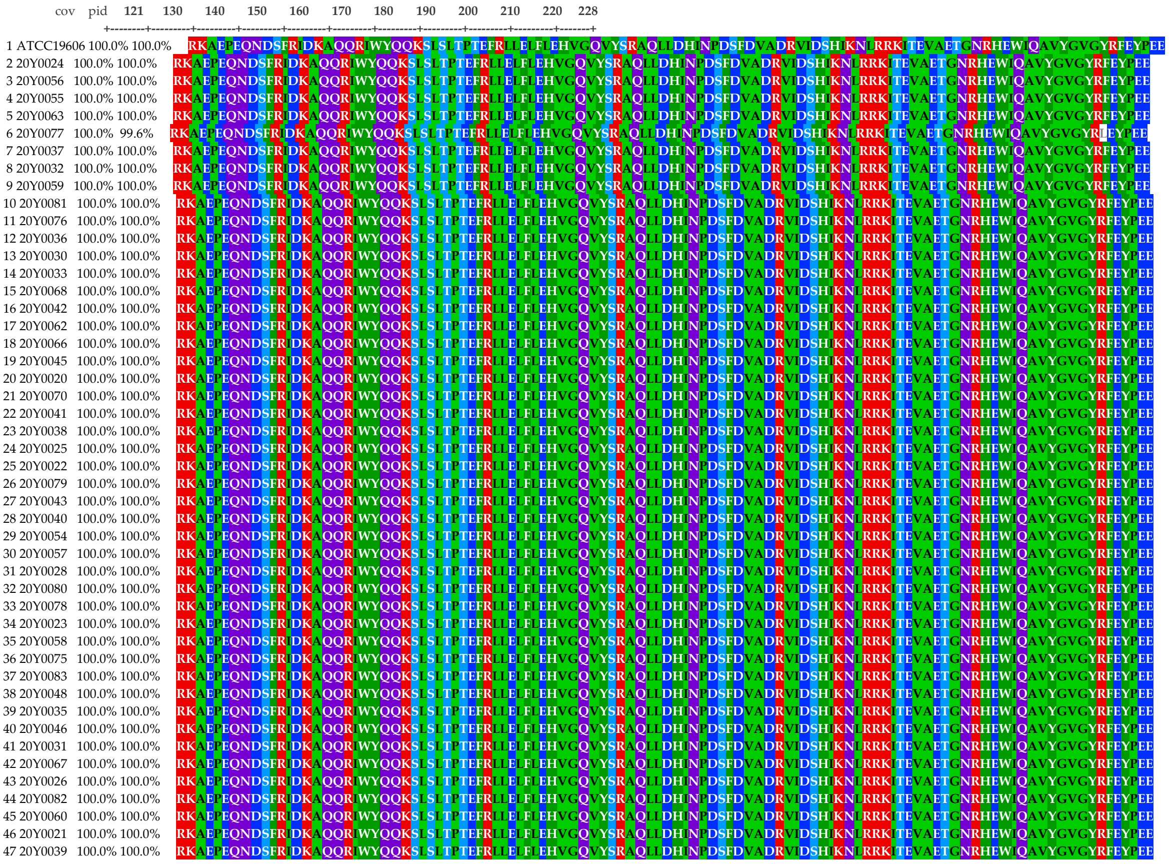

Figure S6: Multiple sequence alignment of the predicted amino acid sequences of BaeR in all isolates compared to that of *A. baumannii* ATCC 19606. The alignment was visualized using MView version 1.63. cov, coverage; pid, percent identity.

```
           cov    pid    1        10        20        30        40        50        60        70        80        90        100       110       120
                         +---------+---------+---------+---------+---------+---------+---------+---------+---------+---------+---------+---------+---------+
1 ATCC19606 100.0% 100.0%
MNVLRVPIALRLFLTVLLTTLLIATASLSVLHWTMQKNFAKYVADVEMQKLDRLITNLGNVYTVYHDWGNAIQAQILQIEGTAAPDDYDRLSQWWLRRQYDIAIQQHSFDEHTLINISPD
2 20Y0056    93.8%  99.4%  ----------------------------------MQKNFAKYVADVEMQKLDRLITNLGNVYTVYHDWGNAIQAQILQIEGTAAPDDYDRLSQWWLRRQYDIAIQQHSFDEHTLINISPD
3 20Y0079   100.0%  99.6%
MNVLRVPIALRLFLTVLLTTLLIATASLSVLHWTMQKNFAKYVADVEMQKLDRLITNLGNVYTVYHDWGNAIQAQILQIEGTAAPDDYDRLSQWWLRRQYDIAIQQHSFDEHTLINISPD
4 20Y0022    93.8%  99.6%  ----------------------------------MQKNFAKYVADVEMQKLDRLITNLGNVYTVYHDWGNAIQAQILQIEGTAAPDDYDRLSQWWLRRQYDIAIQQHSFDEHTLINISPD
5 20Y0046    93.8%  99.6%  ----------------------------------MQKNFAKYVADVEMQKLDRLITNLGNVYTVYHDWGNAIQAQILQIEGTAAPDDYDRLSQWWLRRQYDIAIQQHSFDEHTLINISPD
6 20Y0037    93.8%  99.4%  ----------------------------------MQKNFAKYVADVEMQKLDRLITNLGNVYTVYHDWGNAIQAQILQIEGTAAPDDYDRLSQWWLRRQYDIAIQQHSFDEHTLINISPD
7 20Y0063    93.8%  99.4%  ----------------------------------MQKNFAKYVADVEMQKLDRLITNLGNVYTVYHDWGNAIQAQILQIEGTAAPDDYDRLSQWWLRRQYDIAIQQHSFDEHTLINISPD
8 20Y0023    93.8%  99.4%  ----------------------------------MQKNFAKYVADVEMQKLDRLITNLGNVYTVYHDWGNAIQAQILQIEGTAAPDDYDRLSQWWLRRQYDIAIQQHSFDEHTLINISPD
9 20Y0041    93.8%  99.4%  ----------------------------------MQKNFAKYVADVEMQKLDRLITNLGNVYTVYHDWGNAIQAQILQIEGTAAPDDYDRLSQWWLRRQYDIAIQQHSFDEHTLINISPD
10 20Y0042   93.8%  99.4%  ----------------------------------MQKNFAKYVADVEMQKLDRLITNLGNVYTVYHDWGNAIQAQILQIEGTAAPDDYDRLSQWWLRRQYDIAIQQHSFDEHTLINISPD
11 20Y0025   93.8%  99.4%  ----------------------------------MQKNFAKYVADVEMQKLDRLITNLGNVYTVYHDWGNAIQAQILQIEGTAAPDDYDRLSQWWLRRQYDIAIQQHSFDEHTLINISPD
12 20Y0045   93.8%  99.6%  ----------------------------------MQKNFAKYVADVEMQKLDRLITNLGNVYTVYHDWGNAIQAQILQIEGTAAPDDYDRLSQWWLRRQYDIAIQQHSFDEHTLINISPD
13 20Y0060   93.8%  99.4%  ----------------------------------MQKNFAKYVADVEMQKLDRLITNLGNVYTVYHDWGNAIQAQILQIEGTAAPDDYDRLSQWWLRRQYDIAIQQHSFDEHTLINISPD
14 20Y0035   93.8%  99.6%  ----------------------------------MQKNFAKYVADVEMQKLDRLITNLGNVYTVYHDWGNAIQAQILQIEGTAAPDDYDRLSQWWLRRQYDIAIQQHSFDEHTLINISPD
15 20Y0028   93.8%  99.4%  ----------------------------------MQKNFAKYVADVEMQKLDRLITNLGNVYTVYHDWGNAIQAQILQIEGTAAPDDYDRLSQWWLRRQYDIAIQQHSFDEHTLINISPD
16 20Y0068   93.8%  99.4%  ----------------------------------MQKNFAKYVADVEMQKLDRLITNLGNVYTVYHDWGNAIQAQILQIEGTAAPDDYDRLSQWWLRRQYDIAIQQHSFDEHTLINISPD
17 20Y0057   93.8%  99.6%  ----------------------------------MQKNFAKYVADVEMQKLDRLITNLGNVYTVYHDWGNAIQAQILQIEGTAAPDDYDRLSQWWLRRQYDIAIQQHSFDEHTLINISPD
18 20Y0070   93.8%  99.4%  ----------------------------------MQKNFAKYVADVEMQKLDRLITNLGNVYTVYHDWGNAIQAQILQIEGTAAPDDYDRLSQWWLRRQYDIAIQQHSFDEHTLINISPD
19 20Y0040   93.8%  99.4%  ----------------------------------MQKNFAKYVADVEMQKLDRLITNLGNVYTVYHDWGNAIQAQILQIEGTAAPDDYDRLSQWWLRRQYDIAIQQHSFDEHTLINISPD
20 20Y0066   93.8%  99.4%  ----------------------------------MQKNFAKYVADVEMQKLDRLITNLGNVYTVYHDWGNAIQAQILQIEGTAAPDDYDRLSQWWLRRQYDIAIQQHSFDEHTLINISPD
21 20Y0021   93.8%  99.6%  ----------------------------------MQKNFAKYVADVEMQKLDRLITNLGNVYTVYHDWGNAIQAQILQIEGTAAPDDYDRLSQWWLRRQYDIAIQQHSFDEHTLINISPD
22 20Y0081   93.8%  99.6%  ----------------------------------MQKNFAKYVADVEMQKLDRLITNLGNVYTVYHDWGNAIQAQILQIEGTAAPDDYDRLSQWWLRRQYDIAIQQHSFDEHTLINISPD
23 20Y0032   93.8%  99.4%  ----------------------------------MQKNFAKYVADVEMQKLDRLITNLGNVYTVYHDWGNAIQAQILQIEGTAAPDDYDRLSQWWLRRQYDIAIQQHSFDEHTLINISPD
24 20Y0054   93.8%  99.4%  ----------------------------------MQKNFAKYVADVEMQKLDRLITNLGNVYTVYHDWGNAIQAQILQIEGTAAPDDYDRLSQWWLRRQYDIAIQQHSFDEHTLINISPD
25 20Y0039   93.8%  99.4%  ----------------------------------MQKNFAKYVADVEMQKLDRLITNLGNVYTVYHDWGNAIQAQILQIEGTAAPDDYDRLSQWWLRRQYDIAIQQHSFDEHTLINISPD
26 20Y0031   93.8%  99.4%  ----------------------------------MQKNFAKYVADVEMQKLDRLITNLGNVYTVYHDWGNAIQAQILQIEGTAAPDDYDRLSQWWLRRQYDIAIQQHSFDEHTLINISPD
27 20Y0075   93.8%  99.4%  ----------------------------------MQKNFAKYVADVEMQKLDRLITNLGNVYTVYHDWGNAIQAQILQIEGTAAPDDYDRLSQWWLRRQYDIAIQQHSFDEHTLINISPD
28 20Y0080   93.8%  99.6%  ----------------------------------MQKNFAKYVADVEMQKLDRLITNLGNVYTVYHDWGNAIQAQILQIEGTAAPDDYDRLSQWWLRRQYDIAIQQHSFDEHTLINISPD
29 20Y0020   93.8%  99.4%  ----------------------------------MQKNFAKYVADVEMQKLDRLITNLGNVYTVYHDWGNAIQAQILQIEGTAAPDDYDRLSQWWLRRQYDIAIQQHSFDEHTLINISPD
30 20Y0036   93.8%  99.4%  ----------------------------------MQKNFAKYVADVEMQKLDRLITNLGNVYTVYHDWGNAIQAQILQIEGTAAPDDYDRLSQWWLRRQYDIAIQQHSFDEHTLINISPD
31 20Y0083   93.8%  99.6%  ----------------------------------MQKNFAKYVADVEMQKLDRLITNLGNVYTVYHDWGNAIQAQILQIEGTAAPDDYDRLSQWWLRRQYDIAIQQHSFDEHTLINISPD
32 20Y0076   93.8%  99.6%  ----------------------------------MQKNFAKYVADVEMQKLDRLITNLGNVYTVYHDWGNAIQAQILQIEGTAAPDDYDRLSQWWLRRQYDIAIQQHSFDEHTLINISPD
33 20Y0078   93.8%  99.6%  ----------------------------------MQKNFAKYVADVEMQKLDRLITNLGNVYTVYHDWGNAIQAQILQIEGTAAPDDYDRLSQWWLRRQYDIAIQQHSFDEHTLINISPD
34 20Y0043   93.8%  99.4%  ----------------------------------MQKNFAKYVADVEMQKLDRLITNLGNVYTVYHDWGNAIQAQILQIEGTAAPDDYDRLSQWWLRRQYDIAIQQHSFDEHTLINISPD
35 20Y0048   93.8%  99.4%  ----------------------------------MQKNFAKYVADVEMQKLDRLITNLGNVYTVYHDWGNAIQAQILQIEGTAAPDDYDRLSQWWLRRQYDIAIQQHSFDEHTLINISPD
36 20Y0026   93.8%  99.4%  ----------------------------------MQKNFAKYVADVEMQKLDRLITNLGNVYTVYHDWGNAIQAQILQIEGTAAPDDYDRLSQWWLRRQYDIAIQQHSFDEHTLINISPD
37 20Y0030   93.8%  99.4%  ----------------------------------MQKNFAKYVADVEMQKLDRLITNLGNVYTVYHDWGNAIQAQILQIEGTAAPDDYDRLSQWWLRRQYDIAIQQHSFDEHTLINISPD
38 20Y0082   93.8%  99.6%  ----------------------------------MQKNFAKYVADVEMQKLDRLITNLGNVYTVYHDWGNAIQAQILQIEGTAAPDDYDRLSQWWLRRQYDIAIQQHSFDEHTLINISPD
39 20Y0024   93.8%  99.4%  ----------------------------------MQKNFAKYVADVEMQKLDRLITNLGNVYTVYHDWGNAIQAQILQIEGTAAPDDYDRLSQWWLRRQYDIAIQQHSFDEHTLINISPD
40 20Y0038   93.8%  99.4%  ----------------------------------MQKNFAKYVADVEMQKLDRLITNLGNVYTVYHDWGNAIQAQILQIEGTAAPDDYDRLSQWWLRRQYDIAIQQHSFDEHTLINISPD
41 20Y0059   93.8%  99.4%  ----------------------------------MQKNFAKYVADVEMQKLDRLITNLGNVYTVYHDWGNAIQAQILQIEGTAAPDDYDRLSQWWLRRQYDIAIQQHSFDEHTLINISPD
42 20Y0067   93.8%  99.4%  ----------------------------------MQKNFAKYVADVEMQKLDRLITNLGNVYTVYHDWGNAIQAQILQIEGTAAPDDYDRLSQWWLRRQYDIAIQQHSFDEHTLINISPD
43 20Y0055   93.8%  99.4%  ----------------------------------MQKNFAKYVADVEMQKLDRLITNLGNVYTVYHDWGNAIQAQILQIEGTAAPDDYDRLSQWWLRRQYDIAIQQHSFDEHTLINISPD
44 20Y0077   98.4%  99.4%  ---------
MRLFLTVLLTTLLIATASLSVLHWTMQKNFAKYVADVEMQKLDRLITNLGNVYTVYHDWGNAIQAQILQIEGTAAPDDYDRLSQWWLRRQYDIAIQQHSFDEHTLINISPD
45 20Y0058   93.8%  99.6%  ----------------------------------MQKNFAKYVADVEMQKLDRLITNLGNVYTVYHDWGNAIQAQILQIEGTAAPDDYDRLSQWWLRRQYDIAIQQHSFDEHTLINISPD
46 20Y0062   93.8%  99.4%  ----------------------------------MQKNFAKYVADVEMQKLDRLITNLGNVYTVYHDWGNAIQAQILQIEGTAAPDDYDRLSQWWLRRQYDIAIQQHSFDEHTLINISPD
47 20Y0033   93.8%  99.4%  ----------------------------------MQKNFAKYVADVEMQKLDRLITNLGNVYTVYHDWGNAIQAQILQIEGTAAPDDYDRLSQWWLRRQYDIAIQQHSFDEHTLINISPD

           cov    pid    121      130       140       150       160       170       180       190       200       210       220       230       240
                         +---------+---------+---------+---------+---------+---------+---------+---------+---------+---------+---------+---------+---------+
1 ATCC19606 100.0% 100.0%
AGTVNKNSIFSDEELRTLEQNLPSEYQPFEGLRFPLSANQFRPSDDKNKSKKGSLKDIETQNGKKRFIALPDRLGLSSRLSLYDAKHQFVVGEPPSSDQMSYRPIMVNSRVVGYLGLKPV
2 20Y0056    93.8%  99.4%
AGTVNKNSIFSDEELRTLEQNLPSEYQPFEGLRFPLSANQFRPSDDKNKSKKGSLKDIETQNGKKRFIALPDRLGLSSRLSLYDAKHQFVVGEPPSSDQMSYRPIMVNSQVVGYLGLKPV
3 20Y0079   100.0%  99.6%
AGTVNKNSIFSDEELRTLEQNLPSEYQPFEGLRFPLSANQFRPSDDKNKSKKGSLKDIETQNGKKRFIALPDRLGLSSRLSLYDAKHQFVVGEPPSSDQMSYRPIMVNSQVVGYLGLKPV
4 20Y0022    93.8%  99.6%
AGTVNKNSIFSDEELRTLEQNLPSEYQPFEGLRFPLSANQFRPSDDKNKSKKGSLKDIETQNGKKRFIALPDRLGLSSRLSLYDAKHQFVVGEPPSSDQMSYRPIMVNSQVVGYLGLKPV
5 20Y0046    93.8%  99.6%
AGTVNKNSIFSDEELRTLEQNLPSEYQPFEGLRFPLSANQFRPSDDKNKSKKGSLKDIETQNGKKRFIALPDRLGLSSRLSLYDAKHQFVVGEPPSSDQMSYRPIMVNSQVVGYLGLKPV
6 20Y0037    93.8%  99.4%
AGTVNKNSIFSDEELRTLEQNLPSEYQPFEGLRFPLSANQFRPSDDKNKSKKGSLKDIETQNGKKRFIALPDRLGLSSRLSLYDAKHQFVVGEPPSSDQMSYRPIMVNSQVVGYLGLKPV
7 20Y0063    93.8%  99.4%
AGTVNKNSIFSDEELRTLEQNLPSEYQPFEGLRFPLSANQFRPSDDKNKSKKGSLKDIETQNGKKRFIALPDRLGLSSRLSLYDAKHQFVVGEPPSSDQMSYRPIMVNSQVVGYLGLKPV
8 20Y0023    93.8%  99.4%
AGTVNKNSIFSDEELRTLEQNLPSEYQPFEGLRFPLSANQFRPSDDKNKSKKGSLKDIETQNGKKRFIALPDRLGLSSRLSLYDAKHQFVVGEPPSSDQMSYRPIMVNSQVVGYLGLKPV
9 20Y0041    93.8%  99.4%
AGTVNKNSIFSDEELRTLEQNLPSEYQPFEGLRFPLSANQFRPSDDKNKSKKGSLKDIETQNGKKRFIALPDRLGLSSRLSLYDAKHQFVVGEPPSSDQMSYRPIMVNSQVVGYLGLKPV
10 20Y0042   93.8%  99.4%
AGTVNKNSIFSDEELRTLEQNLPSEYQPFEGLRFPLSANQFRPSDDKNKSKKGSLKDIETQNGKKRFIALPDRLGLSSRLSLYDAKHQFVVGEPPSSDQMSYRPIMVNSQVVGYLGLKPV
11 20Y0025   93.8%  99.4%
AGTVNKNSIFSDEELRTLEQNLPSEYQPFEGLRFPLSANQFRPSDDKNKSKKGSLKDIETQNGKKRFIALPDRLGLSSRLSLYDAKHQFVVGEPPSSDQMSYRPIMVNSQVVGYLGLKPV
12 20Y0045   93.8%  99.6%
AGTVNKNSIFSDEELRTLEQNLPSEYQPFEGLRFPLSANQFRPSDDKNKSKKGSLKDIETQNGKKRFIALPDRLGLSSRLSLYDAKHQFVVGEPPSSDQMSYRPIMVNSQVVGYLGLKPV
13 20Y0060   93.8%  99.4%
AGTVNKNSIFSDEELRTLEQNLPSEYQPFEGLRFPLSANQFRPSDDKNKSKKGSLKDIETQNGKKRFIALPDRLGLSSRLSLYDAKHQFVVGEPPSSDQMSYRPIMVNSQVVGYLGLKPV
14 20Y0035   93.8%  99.6%
AGTVNKNSIFSDEELRTLEQNLPSEYQPFEGLRFPLSANQFRPSDDKNKSKKGSLKDIETQNGKKRFIALPDRLGLSSRLSLYDAKHQFVVGEPPSSDQMSYRPIMVNSQVVGYLGLKPV
15 20Y0028   93.8%  99.4%
AGTVNKNSIFSDEELRTLEQNLPSEYQPFEGLRFPLSANQFRPSDDKNKSKKGSLKDIETQNGKKRFIALPDRLGLSSRLSLYDAKHQFVVGEPPSSDQMSYRPIMVNSQVVGYLGLKPV
16 20Y0068   93.8%  99.4%
AGTVNKNSIFSDEELRTLEQNLPSEYQPFEGLRFPLSANQFRPSDDKNKSKKGSLKDIETQNGKKRFIALPDRLGLSSRLSLYDAKHQFVVGEPPSSDQMSYRPIMVNSQVVGYLGLKPV
17 20Y0057   93.8%  99.6%
AGTVNKNSIFSDEELRTLEQNLPSEYQPFEGLRFPLSANQFRPSDDKNKSKKGSLKDIETQNGKKRFIALPDRLGLSSRLSLYDAKHQFVVGEPPSSDQMSYRPIMVNSQVVGYLGLKPV
18 20Y0070   93.8%  99.4%
AGTVNKNSIFSDEELRTLEQNLPSEYQPFEGLRFPLSANQFRPSDDKNKSKKGSLKDIETQNGKKRFIALPDRLGLSSRLSLYDAKHQFVVGEPPSSDQMSYRPIMVNSQVVGYLGLKPV
19 20Y0040   93.8%  99.4%
AGTVNKNSIFSDEELRTLEQNLPSEYQPFEGLRFPLSANQFRPSDDKNKSKKGSLKDIETQNGKKRFIALPDRLGLSSRLSLYDAKHQFVVGEPPSSDQMSYRPIMVNSQVVGYLGLKPV
20 20Y0066   93.8%  99.4%
AGTVNKNSIFSDEELRTLEQNLPSEYQPFEGLRFPLSANQFRPSDDKNKSKKGSLKDIETQNGKKRFIALPDRLGLSSRLSLYDAKHQFVVGEPPSSDQMSYRPIMVNSQVVGYLGLKPV
```

21 20Y0021 93.8% 99.6%

AGTVNKNSIFSDEELRTLEQNLPSEYQPFEGLRFPLSANQFRPSDDKNKSKKGSLKDIETQNGKKRFIALPDRLGLSSRLSLYDAKHQFVVGEPPSSDQMSYRPIMVNSQVVGYLGLKPV

22 20Y0081 93.8% 99.6%

AGTVNKNSIFSDEELRTLEQNLPSEYQPFEGLRFPLSANQFRPSDDKNKSKKGSLKDIETQNGKKRFIALPDRLGLSSRLSLYDAKHQFVVGEPPSSDQMSYRPIMVNSQVVGYLGLKPV

23 20Y0032 93.8% 99.4%

AGTVNKNSIFSDEELRTLEQNLPSEYQPFEGLRFPLSANQFRPSDDKNKSKKGSLKDIETQNGKKRFIALPDRLGLSSRLSLYDAKHQFVVGEPPSSDQMSYRPIMVNSQVVGYLGLKPV

24 20Y0054 93.8% 99.4%

AGTVNKNSIFSDEELRTLEQNLPSEYQPFEGLRFPLSANQFRPSDDKNKSKKGSLKDIETQNGKKRFIALPDRLGLSSRLSLYDAKHQFVVGEPPSSDQMSYRPIMVNSQVVGYLGLKPV

25 20Y0039 93.8% 99.4%

AGTVNKNSIFSDEELRTLEQNLPSEYQPFEGLRFPLSANQFRPSDDKNKSKKGSLKDIETQNGKKRFIALPDRLGLSSRLSLYDAKHQFVVGEPPSSDQMSYRPIMVNSQVVGYLGLKPV

26 20Y0031 93.8% 99.4%

AGTVNKNSIFSDEELRTLEQNLPSEYQPFEGLRFPLSANQFRPSDDKNKSKKGSLKDIETQNGKKRFIALPDRLGLSSRLSLYDAKHQFVVGEPPSSDQMSYRPIMVNSQVVGYLGLKPV

27 20Y0075 93.8% 99.4%

AGTVNKNSIFSDEELRTLEQNLPSEYQPFEGLRFPLSANQFRPSDDKNKSKKGSLKDIETQNGKKRFIALPDRLGLSSRLSLYDAKHQFVVGEPPSSDQMSYRPIMVNSQVVGYLGLKPV

28 20Y0080 93.8% 99.6%

AGTVNKNSIFSDEELRTLEQNLPSEYQPFEGLRFPLSANQFRPSDDKNKSKKGSLKDIETQNGKKRFIALPDRLGLSSRLSLYDAKHQFVVGEPPSSDQMSYRPIMVNSQVVGYLGLKPV

29 20Y0020 93.8% 99.4%

AGTVNKNSIFSDEELRTLEQNLPSEYQPFEGLRFPLSANQFRPSDDKNKSKKGSLKDIETQNGKKRFIALPDRLGLSSRLSLYDAKHQFVVGEPPSSDQMSYRPIMVNSQVVGYLGLKPV

30 20Y0036 93.8% 99.4%

AGTVNKNSIFSDEELRTLEQNLPSEYQPFEGLRFPLSANQFRPSDDKNKSKKGSLKDIETQNGKKRFIALPDRLGLSSRLSLYDAKHQFVVGEPPSSDQMSYRPIMVNSQVVGYLGLKPV

31 20Y0083 93.8% 99.6%

AGTVNKNSIFSDEELRTLEQNLPSEYQPFEGLRFPLSANQFRPSDDKNKSKKGSLKDIETQNGKKRFIALPDRLGLSSRLSLYDAKHQFVVGEPPSSDQMSYRPIMVNSQVVGYLGLKPV

32 20Y0076 93.8% 99.6%

AGTVNKNSIFSDEELRTLEQNLPSEYQPFEGLRFPLSANQFRPSDDKNKSKKGSLKDIETQNGKKRFIALPDRLGLSSRLSLYDAKHQFVVGEPPSSDQMSYRPIMVNSQVVGYLGLKPV

33 20Y0078 93.8% 99.6%

AGTVNKNSIFSDEELRTLEQNLPSEYQPFEGLRFPLSANQFRPSDDKNKSKKGSLKDIETQNGKKRFIALPDRLGLSSRLSLYDAKHQFVVGEPPSSDQMSYRPIMVNSQVVGYLGLKPV

34 20Y0043 93.8% 99.4%

AGTVNKNSIFSDEELRTLEQNLPSEYQPFEGLRFPLSANQFRPSDDKNKSKKGSLKDIETQNGKKRFIALPDRLGLSSRLSLYDAKHQFVVGEPPSSDQMSYRPIMVNSQVVGYLGLKPV

35 20Y0048 93.8% 99.4%

AGTVNKNSIFSDEELRTLEQNLPSEYQPFEGLRFPLSANQFRPSDDKNKSKKGSLKDIETQNGKKRFIALPDRLGLSSRLSLYDAKHQFVVGEPPSSDQMSYRPIMVNSQVVGYLGLKPV

36 20Y0026 93.8% 99.4%

AGTVNKNSIFSDEELRTLEQNLPSEYQPFEGLRFPLSANQFRPSDDKNKSKKGSLKDIETQNGKKRFIALPDRLGLSSRLSLYDAKHQFVVGEPPSSDQMSYRPIMVNSQVVGYLGLKPV

37 20Y0030 93.8% 99.4%

AGTVNKNSIFSDEELRTLEQNLPSEYQPFEGLRFPLSANQFRPSDDKNKSKKGSLKDIETQNGKKRFIALPDRLGLSSRLSLYDAKHQFVVGEPPSSDQMSYRPIMVNSQVVGYLGLKPV

38 20Y0082 93.8% 99.6%

AGTVNKNSIFSDEELRTLEQNLPSEYQPFEGLRFPLSANQFRPSDDKNKSKKGSLKDIETQNGKKRFIALPDRLGLSSRLSLYDAKHQFVVGEPPSSDQMSYRPIMVNSQVVGYLGLKPV

39 20Y0024 93.8% 99.4%

AGTVNKNSIFSDEELRTLEQNLPSEYQPFEGLRFPLSANQFRPSDDKNKSKKGSLKDIETQNGKKRFIALPDRLGLSSRLSLYDAKHQFVVGEPPSSDQMSYRPIMVNSQVVGYLGLKPV

40 20Y0038 93.8% 99.4%

AGTVNKNSIFSDEELRTLEQNLPSEYQPFEGLRFPLSANQFRPSDDKNKSKKGSLKDIETQNGKKRFIALPDRLGLSSRLSLYDAKHQFVVGEPPSSDQMSYRPIMVNSQVVGYLGLKPV

41 20Y0059 93.8% 99.4%

AGTVNKNSIFSDEELRTLEQNLPSEYQPFEGLRFPLSANQFRPSDDKNKSKKGSLKDIETQNGKKRFIALPDRLGLSSRLSLYDAKHQFVVGEPPSSDQMSYRPIMVNSQVVGYLGLKPV

42 20Y0067 93.8% 99.4%

AGTVNKNSIFSDEELRTLEQNLPSEYQPFEGLRFPLSANQFRPSDDKNKSKKGSLKDIETQNGKKRFIALPDRLGLSSRLSLYDAKHQFVVGEPPSSDQMSYRPIMVNSQVVGYLGLKPV

43 20Y0055 93.8% 99.4%

AGTVNKNSIFSDEELRTLEQNLPSEYQPFEGLRFPLSANQFRPSDDKNKSKKGSLKDIETQNGKKRFIALPDRLGLSSRLSLYDAKHQFVVGEPPSSDQMSYRPIMVNSQVVGYLGLKPV

44 20Y0077 98.4% 99.4%

AGTVNKNSIFSDEELRTLEQNLPSEYQPFEGLRFPLSANQFRPSDDKNKSKKGSLKDIETQNGKKRFIALPDRLGLSSRLSLYDAKHQFVVGEPPSSDQMSYRPIMVNSQVVGYLGLKPV

45 20Y0058 93.8% 99.6%

AGTVNKNSIFSDEELRTLEQNLPSEYQPFEGLRFPLSANQFRPSDDKNKSKKGSLKDIETQNGKKRFIALPDRLGLSSRLSLYDAKHQFVVGEPPSSDQMSYRPIMVNSQVVGYLGLKPV

46 20Y0062 93.8% 99.4%

AGTVNKNSIFSDEELRTLEQNLPSEYQPFEGLRFPLSANQFRPSDDKNKSKKGSLKDIETQNGKKRFIALPDRLGLSSRLSLYDAKHQFVVGEPPSSDQMSYRPIMVNSQVVGYLGLKPV

47 20Y0033 93.8% 99.4%

AGTVNKNSIFSDEELRTLEQNLPSEYQPFEGLRFPLSANQFRPSDDKNKSKKGSLKDIETQNGKKRFIALPDRLGLSSRLSLYDAKHQFVVGEPPSSDQMSYRPIMVNSQVVGYLGLKPV

```
              cov    pid   241     250     260     270     280     290     300     310     320     330     340     350     360
                           +--------+---------+---------+---------+---------+---------+---------+---------+---------+---------+---------+---------+
1 ATCC19606 100.0% 100.0%
LDKEDALSINFFSNQKRYLLLIYALTLLSSLVAALLMATYFKKPIQRLLNATNELTKGNYQHQVVIKRNDELGDLSTQLNHLAEILHQHEESRRQWVADTSHELKTPLAVLQAQIEAMQD
2 20Y0056    93.8%  99.4%
LDKEDALSINFFSNQKRYLLLIYALTLLSSLVAALLMATYFKKPIQRLLNATNELTKGNYQHQVVIKRNDELGDLSTQLNHLAEILHQHEESRRQWVADTSHELKTPLAVLQAQIEAMQD
3 20Y0079   100.0%  99.6%
LDKEDALSINFFSNQKRYLLLIYALTLLSSLVAALLMATYFKKPIQRLLNATNELTKGNYQHQVVIKRNDELGDLSTQLNHLAEILHQHEESRRQWVADTSHELKTPLAVLQAQIEAMQD
4 20Y0022    93.8%  99.6%
LDKEDALSINFFSNQKRYLLLIYALTLLSSLVAALLMATYFKKPIQRLLNATNELTKGNYQHQVVIKRNDELGDLSTQLNHLAEILHQHEESRRQWVADTSHELKTPLAVLQAQIEAMQD
5 20Y0046    93.8%  99.6%
LDKEDALSINFFSNQKRYLLLIYALTLLSSLVAALLMATYFKKPIQRLLNATNELTKGNYQHQVVIKRNDELGDLSTQLNHLAEILHQHEESRRQWVADTSHELKTPLAVLQAQIEAMQD
6 20Y0037    93.8%  99.4%
LDKEDALSINFFSNQKRYLLLIYALTLLSSLVAALLMATYFKKPIQRLLNATNELTKGNYQHQVVIKRNDELGDLSTQLNHLAEILHQHEESRRQWVADTSHELKTPLAVLQAQIEAMQD
7 20Y0063    93.8%  99.4%
LDKEDALSINFFSNQKRYLLLIYALTLLSSLVAALLMATYFKKPIQRLLNATNELTKGNYQHQVVIKRNDELGDLSTQLNHLAEILHQHEESRRQWVADTSHELKTPLAVLQAQIEAMQD
8 20Y0023    93.8%  99.4%
LDKEDALSINFFSNQKRYLLLIYALTLLSSLVAALLMATYFKKPIQRLLNATNELTKGNYQHQVVIKRNDELGDLSTQLNHLAEILHQHEESRRQWVADTSHELKTPLAVLQAQIEAMQD
9 20Y0041    93.8%  99.4%
LDKEDALSINFFSNQKRYLLLIYALTLLSSLVAALLMATYFKKPIQRLLNATNELTKGNYQHQVVIKRNDELGDLSTQLNHLAEILHQHEESRRQWVADTSHELKTPLAVLQAQIEAMQD
10 20Y0042    93.8%  99.4%
LDKEDALSINFFSNQKRYLLLIYALTLLSSLVAALLMATYFKKPIQRLLNATNELTKGNYQHQVVIKRNDELGDLSTQLNHLAEILHQHEESRRQWVADTSHELKTPLAVLQAQIEAMQD
11 20Y0025    93.8%  99.4%
LDKEDALSINFFSNQKRYLLLIYALTLLSSLVAALLMATYFKKPIQRLLNATNELTKGNYQHQVVIKRNDELGDLSTQLNHLAEILHQHEESRRQWVADTSHELKTPLAVLQAQIEAMQD
12 20Y0045    93.8%  99.6%
LDKEDALSINFFSNQKRYLLLIYALTLLSSLVAALLMATYFKKPIQRLLNATNELTKGNYQHQVVIKRNDELGDLSTQLNHLAEILHQHEESRRQWVADTSHELKTPLAVLQAQIEAMQD
13 20Y0060    93.8%  99.4%
LDKEDALSINFFSNQKRYLLLIYALTLLSSLVAALLMATYFKKPIQRLLNATNELTKGNYQHQVVIKRNDELGDLSTQLNHLAEILHQHEESRRQWVADTSHELKTPLAVLQAQIEAMQD
14 20Y0035    93.8%  99.6%
LDKEDALSINFFSNQKRYLLLIYALTLLSSLVAALLMATYFKKPIQRLLNATNELTKGNYQHQVVIKRNDELGDLSTQLNHLAEILHQHEESRRQWVADTSHELKTPLAVLQAQIEAMQD
15 20Y0028    93.8%  99.4%
LDKEDALSINFFSNQKRYLLLIYALTLLSSLVAALLMATYFKKPIQRLLNATNELTKGNYQHQVVIKRNDELGDLSTQLNHLAEILHQHEESRRQWVADTSHELKTPLAVLQAQIEAMQD
16 20Y0068    93.8%  99.4%
LDKEDALSINFFSNQKRYLLLIYALTLLSSLVAALLMATYFKKPIQRLLNATNELTKGNYQHQVVIKRNDELGDLSTQLNHLAEILHQHEESRRQWVADTSHELKTPLAVLQAQIEAMQD
17 20Y0057    93.8%  99.6%
LDKEDALSINFFSNQKRYLLLIYALTLLSSLVAALLMATYFKKPIQRLLNATNELTKGNYQHQVVIKRNDELGDLSTQLNHLAEILHQHEESRRQWVADTSHELKTPLAVLQAQIEAMQD
18 20Y0070    93.8%  99.4%
LDKEDALSINFFSNQKRYLLLIYALTLLSSLVAALLMATYFKKPIQRLLNATNELTKGNYQHQVVIKRNDELGDLSTQLNHLAEILHQHEESRRQWVADTSHELKTPLAVLQAQIEAMQD
19 20Y0040    93.8%  99.4%
LDKEDALSINFFSNQKRYLLLIYALTLLSSLVAALLMATYFKKPIQRLLNATNELTKGNYQHQVVIKRNDELGDLSTQLNHLAEILHQHEESRRQWVADTSHELKTPLAVLQAQIEAMQD
20 20Y0066    93.8%  99.4%
LDKEDALSINFFSNQKRYLLLIYALTLLSSLVAALLMATYFKKPIQRLLNATNELTKGNYQHQVVIKRNDELGDLSTQLNHLAEILHQHEESRRQWVADTSHELKTPLAVLQAQIEAMQD
21 20Y0021    93.8%  99.6%
LDKEDALSINFFSNQKRYLLLIYALTLLSSLVAALLMATYFKKPIQRLLNATNELTKGNYQHQVVIKRNDELGDLSTQLNHLAEILHQHEESRRQWVADTSHELKTPLAVLQAQIEAMQD
22 20Y0081    93.8%  99.6%
LDKEDALSINFFSNQKRYLLLIYALTLLSSLVAALLMATYFKKPIQRLLNATNELTKGNYQHQVVIKRNDELGDLSTQLNHLAEILHQHEESRRQWVADTSHELKTPLAVLQAQIEAMQD
23 20Y0032    93.8%  99.4%
LDKEDALSINFFSNQKRYLLLIYALTLLSSLVAALLMATYFKKPIQRLLNATNELTKGNYQHQVVIKRNDELGDLSTQLNHLAEILHQHEESRRQWVADTSHELKTPLAVLQAQIEAMQD
24 20Y0054    93.8%  99.4%
LDKEDALSINFFSNQKRYLLLIYALTLLSSLVAALLMATYFKKPIQRLLNATNELTKGNYQHQVVIKRNDELGDLSTQLNHLAEILHQHEESRRQWVADTSHELKTPLAVLQAQIEAMQD
25 20Y0039    93.8%  99.4%
LDKEDALSINFFSNQKRYLLLIYALTLLSSLVAALLMATYFKKPIQRLLNATNELTKGNYQHQVVIKRNDELGDLSTQLNHLAEILHQHEESRRQWVADTSHELKTPLAVLQAQIEAMQD
26 20Y0031    93.8%  99.4%
LDKEDALSINFFSNQKRYLLLIYALTLLSSLVAALLMATYFKKPIQRLLNATNELTKGNYQHQVVIKRNDELGDLSTQLNHLAEILHQHEESRRQWVADTSHELKTPLAVLQAQIEAMQD
27 20Y0075    93.8%  99.4%
LDKEDALSINFFSNQKRYLLLIYALTLLSSLVAALLMATYFKKPIQRLLNATNELTKGNYQHQVVIKRNDELGDLSTQLNHLAEILHQHEESRRQWVADTSHELKTPLAVLQAQIEAMQD
28 20Y0080    93.8%  99.6%
LDKEDALSINFFSNQKRYLLLIYALTLLSSLVAALLMATYFKKPIQRLLNATNELTKGNYQHQVVIKRNDELGDLSTQLNHLAEILHQHEESRRQWVADTSHELKTPLAVLQAQIEAMQD
29 20Y0020    93.8%  99.4%
LDKEDALSINFFSNQKRYLLLIYALTLLSSLVAALLMATYFKKPIQRLLNATNELTKGNYQHQVVIKRNDELGDLSTQLNHLAEILHQHEESRRQWVADTSHELKTPLAVLQAQIEAMQD
30 20Y0036    93.8%  99.4%
LDKEDALSINFFSNQKRYLLLIYALTLLSSLVAALLMATYFKKPIQRLLNATNELTKGNYQHQVVIKRNDELGDLSTQLNHLAEILHQHEESRRQWVADTSHELKTPLAVLQAQIEAMQD
31 20Y0083    93.8%  99.6%
LDKEDALSINFFSNQKRYLLLIYALTLLSSLVAALLMATYFKKPIQRLLNATNELTKGNYQHQVVIKRNDELGDLSTQLNHLAEILHQHEESRRQWVADTSHELKTPLAVLQAQIEAMQD
32 20Y0076    93.8%  99.6%
LDKEDALSINFFSNQKRYLLLIYALTLLSSLVAALLMATYFKKPIQRLLNATNELTKGNYQHQVVIKRNDELGDLSTQLNHLAEILHQHEESRRQWVADTSHELKTPLAVLQAQIEAMQD
33 20Y0078    93.8%  99.6%
LDKEDALSINFFSNQKRYLLLIYALTLLSSLVAALLMATYFKKPIQRLLNATNELTKGNYQHQVVIKRNDELGDLSTQLNHLAEILHQHEESRRQWVADTSHELKTPLAVLQAQIEAMQD
34 20Y0043    93.8%  99.4%
LDKEDALSINFFSNQKRYLLLIYALTLLSSLVAALLMATYFKKPIQRLLNATNELTKGNYQHQVVIKRNDELGDLSTQLNHLAEILHQHEESRRQWVADTSHELKTPLAVLQAQIEAMQD
35 20Y0048    93.8%  99.4%
LDKEDALSINFFSNQKRYLLLIYALTLLSSLVAALLMATYFKKPIQRLLNATNELTKGNYQHQVVIKRNDELGDLSTQLNHLAEILHQHEESRRQWVADTSHELKTPLAVLQAQIEAMQD
36 20Y0026    93.8%  99.4%
LDKEDALSINFFSNQKRYLLLIYALTLLSSLVAALLMATYFKKPIQRLLNATNELTKGNYQHQVVIKRNDELGDLSTQLNHLAEILHQHEESRRQWVADTSHELKTPLAVLQAQIEAMQD
37 20Y0030    93.8%  99.4%
LDKEDALSINFFSNQKRYLLLIYALTLLSSLVAALLMATYFKKPIQRLLNATNELTKGNYQHQVVIKRNDELGDLSTQLNHLAEILHQHEESRRQWVADTSHELKTPLAVLQAQIEAMQD
38 20Y0082    93.8%  99.6%
LDKEDALSINFFSNQKRYLLLIYALTLLSSLVAALLMATYFKKPIQRLLNATNELTKGNYQHQVVIKRNDELGDLSTQLNHLAEILHQHEESRRQWVADTSHELKTPLAVLQAQIEAMQD
39 20Y0024    93.8%  99.4%
LDKEDALSINFFSNQKRYLLLIYALTLLSSLVAALLMATYFKKPIQRLLNATNELTKGNYQHQVVIKRNDELGDLSTQLNHLAEILHQHEESRRQWVADTSHELKTPLAVLQAQIEAMQD
40 20Y0038    93.8%  99.4%
LDKEDALSINFFSNQKRYLLLIYALTLLSSLVAALLMATYFKKPIQRLLNATNELTKGNYQHQVVIKRNDELGDLSTQLNHLAEILHQHEESRRQWVADTSHELKTPLAVLQAQIEAMQD
41 20Y0059    93.8%  99.4%
LDKEDALSINFFSNQKRYLLLIYALTLLSSLVAALLMATYFKKPIQRLLNATNELTKGNYQHQVVIKRNDELGDLSTQLNHLAEILHQHEESRRQWVADTSHELKTPLAVLQAQIEAMQD
42 20Y0067    93.8%  99.4%
LDKEDALSINFFSNQKRYLLLIYALTLLSSLVAALLMATYFKKPIQRLLNATNELTKGNYQHQVVIKRNDELGDLSTQLNHLAEILHQHEESRRQWVADTSHELKTPLAVLQAQIEAMQD
43 20Y0055    93.8%  99.4%
LDKEDALSINFFSNQKRYLLLIYALTLLSSLVAALLMATYFKKPIQRLLNATNELTKGNYQHQVVIKRNDELGDLSTQLNHLAEILHQHEESRRQWVADTSHELKTPLAVLQAQIEAMQD
44 20Y0077    98.4%  99.4%
LDKEDALSINFFSNQKRYLLLIYALTLLSSLVAALLMATYFKKPIQRLLNATNELTKGNYQHQVVIKRNDELGDLSTQLNHLAEILHQHEESRRQWVADTSHELKTPLAVLQAQIEAMQD
45 20Y0058    93.8%  99.6%
LDKEDALSINFFSNQKRYLLLIYALTLLSSLVAALLMATYFKKPIQRLLNATNELTKGNYQHQVVIKRNDELGDLSTQLNHLAEILHQHEESRRQWVADTSHELKTPLAVLQAQIEAMQD
46 20Y0062    93.8%  99.4%
LDKEDALSINFFSNQKRYLLLIYALTLLSSLVAALLMATYFKKPIQRLLNATNELTKGNYQHQVVIKRNDELGDLSTQLNHLAEILHQHEESRRQWVADTSHELKTPLAVLQAQIEAMQD
47 20Y0033    93.8%  99.4%
LDKEDALSINFFSNQKRYLLLIYALTLLSSLVAALLMATYFKKPIQRLLNATNELTKGNYQHQVVIKRNDELGDLSTQLNHLAEILHQHEESRRQWVADTSHELKTPLAVLQAQIEAMQD
```

```
            cov    pid    361    370    380    390    400    410    420    430    440    450    460    470    480
                          +---------+---------+---------+---------+---------+---------+---------+---------+---------+---------+---------+---------+
1 ATCC19606 100.0% 100.0%
GIRKATPEHLDAMMRQVSSLKKLTQDLADLAQADAQQLKCYLSSVNPWEVILQEVENFRPKLEQAGLEIEVEGEGASLLLDRDRFKQIIVNLIGNSIRYTETGGKIHIHTNQTPQEWTLY
2 20Y0056    93.8%  99.4%
GIRKATPEHLDAMMRQVSSLKKLTQDLADLAQADAQQLKCYLSSVNPWEVILQEVENFRPKLEQAGLEIEVEGEGATLLLDRDRFKQIIVNLIGNSIRYTETGGKIHIHTSQTPQEWTLY
3 20Y0079   100.0%  99.6%
GIRKATPEHLDAMMRQVSSLKKLTQDLADLAQADAQQLKCYLSSVNPWEVILQEVENFRPKLEQAGLEIEVEGEGASLLLDRDRFKQIIVNLIGNSIRYTETGGKIHIHTNQTPQEWTLY
4 20Y0022    93.8%  99.6%
GIRKATPEHLDAMMRQVSSLKKLTQDLADLAQADAQQLKCYLSSVNPWEVILQEVENFRPKLEQAGLEIEVEGEGASLLLDRDRFKQIIVNLIGNSIRYTETGGKIHIHTSQTPQEWTLY
5 20Y0046    93.8%  99.6%
GIRKATPEHLDAMMRQVSSLKKLTQDLADLAQADAQQLKCYLSSVNPWEVILQEVENFRPKLEQAGLEIEVEGEGASLLLDRDRFKQIIVNLIGNSIRYTETGGKIHIHTSQTPQEWTLY
6 20Y0037    93.8%  99.4%
GIRKATPEHLDAMMRQVSSLKKLTQDLADLAQADAQQLKCYLSSVNPWEVILQEVENFRPKLEQAGLEIEVEGEGATLLLDRDRFKQIIVNLIGNSIRYTETGGKIHIHTSQTPQEWTLY
7 20Y0063    93.8%  99.4%
GIRKATPEHLDAMMRQVSSLKKLTQDLADLAQADAQQLKCYLSSVNPWEVILQEVENFRPKLEQAGLEIEVEGEGATLLLDRDRFKQIIVNLIGNSIRYTETGGKIHIHTSQTPQEWTLY
8 20Y0023    93.8%  99.4%
GIRKATPEHLDAMMRQVSSLKKLTQDLADLAQADAQQLKCYLSSVNPWEVILQEVENFRPKLEQAGLEIEVEGEGATLLLDRDRFKQIIVNLIGNSIRYTETGGKIHIHTSQTPQEWTLY
9 20Y0041    93.8%  99.4%
GIRKATPEHLDAMMRQVSSLKKLTQDLADLAQADAQQLKCYLSSVNPWEVILQEVENFRPKLEQAGLEIEVEGEGATLLLDRDRFKQIIVNLIGNSIRYTETGGKIHIHTSQTPQEWTLY
10 20Y0042   93.8%  99.4%
GIRKATPEHLDAMMRQVSSLKKLTQDLADLAQADAQQLKCYLSSVNPWEVILQEVENFRPKLEQAGLEIEVEGEGATLLLDRDRFKQIIVNLIGNSIRYTETGGKIHIHTSQTPQEWTLY
11 20Y0025   93.8%  99.4%
GIRKATPEHLDAMMRQVSSLKKLTQDLADLAQADAQQLKCYLSSVNPWEVILQEVENFRPKLEQAGLEIEVEGEGATLLLDRDRFKQIIVNLIGNSIRYTETGGKIHIHTSQTPQEWTLY
12 20Y0045   93.8%  99.6%
GIRKATPEHLDAMMRQVSSLKKLTQDLADLAQADAQQLKCYLSSVNPWEVILQEVENFRPKLEQAGLEIEVEGEGASLLLDRDRFKQIIVNLIGNSIRYTETGGKIHIHTSQTPQEWTLY
13 20Y0060   93.8%  99.4%
GIRKATPEHLDAMMRQVSSLKKLTQDLADLAQADAQQLKCYLSSVNPWEVILQEVENFRPKLEQAGLEIEVEGEGATLLLDRDRFKQIIVNLIGNSIRYTETGGKIHIHTSQTPQEWTLY
14 20Y0035   93.8%  99.6%
GIRKATPEHLDAMMRQVSSLKKLTQDLADLAQADAQQLKCYLSSVNPWEVILQEVENFRPKLEQAGLEIEVEGEGASLLLDRDRFKQIIVNLIGNSIRYTETGGKIHIHTSQTPQEWTLY
15 20Y0028   93.8%  99.4%
GIRKATPEHLDAMMRQVSSLKKLTQDLADLAQADAQQLKCYLSSVNPWEVILQEVENFRPKLEQAGLEIEVEGEGATLLLDRDRFKQIIVNLIGNSIRYTETGGKIHIHTSQTPQEWTLY
16 20Y0068   93.8%  99.4%
GIRKATPEHLDAMMRQVSSLKKLTQDLADLAQADAQQLKCYLSSVNPWEVILQEVENFRPKLEQAGLEIEVEGEGATLLLDRDRFKQIIVNLIGNSIRYTETGGKIHIHTSQTPQEWTLY
17 20Y0057   93.8%  99.6%
GIRKATPEHLDAMMRQVSSLKKLTQDLADLAQADAQQLKCYLSSVNPWEVILQEVENFRPKLEQAGLEIEVEGEGTSLLLDRDRFKQIIVNLIGNSIRYTETGGKIHIHTNQTPQEWTLY
18 20Y0070   93.8%  99.4%
GIRKATPEHLDAMMRQVSSLKKLTQDLADLAQADAQQLKCYLSSVNPWEVILQEVENFRPKLEQAGLEIEVEGEGATLLLDRDRFKQIIVNLIGNSIRYTETGGKIHIHTSQTPQEWTLY
19 20Y0040   93.8%  99.4%
GIRKATPEHLDAMMRQVSSLKKLTQDLADLAQADAQQLKCYLSSVNPWEVILQEVENFRPKLEQAGLEIEVEGEGATLLLDRDRFKQIIVNLIGNSIRYTETGGKIHIHTSQTPQEWTLY
20 20Y0066   93.8%  99.4%
GIRKATPEHLDAMMRQVSSLKKLTQDLADLAQADAQQLKCYLSSVNPWEVILQEVENFRPKLEQAGLEIEVEGEGATLLLDRDRFKQIIVNLIGNSIRYTETGGKIHIHTSQTPQEWTLY
21 20Y0021   93.8%  99.6%
GIRKATPEHLDAMMRQVSSLKKLTQDLADLAQADAQQLKCYLSSVNPWEVILQEVENFRPKLEQAGLEIEVEGEGASLLLDRDRFKQIIVNLIGNSIRYTETGGKIHIHTSQTPQEWTLY
22 20Y0081   93.8%  99.6%
GIRKATPEHLDAMMRQVSSLKKLTQDLADLAQADAQQLKCYLSSVNPWEVILQEVENFRPKLEQAGLEIEVEGEGASLLLDRDRFKQIIVNLIGNSIRYTETGGKIHIHTSQTPQEWTLY
23 20Y0032   93.8%  99.4%
GIRKATPEHLDAMMRQVSSLKKLTQDLADLAQADAQQLKCYLSSVNPWEVILQEVENFRPKLEQAGLEIEVEGEGATLLLDRDRFKQIIVNLIGNSIRYTETGGKIHIHTSQTPQEWTLY
24 20Y0054   93.8%  99.4%
GIRKATPEHLDAMMRQVSSLKKLTQDLADLAQADAQQLKCYLSSVNPWEVILQEVENFRPKLEQAGLEIEVEGEGATLLLDRDRFKQIIVNLIGNSIRYTETGGKIHIHTSQTPQEWTLY
25 20Y0039   93.8%  99.4%
GIRKATPEHLDAMMRQVSSLKKLTQDLADLAQADAQQLKCYLSSVNPWEVILQEVENFRPKLEQAGLEIEVEGEGATLLLDRDRFKQIIVNLIGNSIRYTETGGKIHIHTSQTPQEWTLY
26 20Y0031   93.8%  99.4%
GIRKATPEHLDAMMRQVSSLKKLTQDLADLAQADAQQLKCYLSSVNPWEVILQEVENFRPKLEQAGLEIEVEGEGATLLLDRDRFKQIIVNLIGNSIRYTETGGKIHIHTSQTPQEWTLY
27 20Y0075   93.8%  99.4%
GIRKATPEHLDAMMRQVSSLKKLTQDLADLAQADAQQLKCYLSSVNPWEVILQEVENFRPKLEQAGLEIEVEGEGATLLLDRDRFKQIIVNLIGNSIRYTETGGKIHIHTSQTPQEWTLY
28 20Y0080   93.8%  99.6%
GIRKATPEHLDAMMRQVSSLKKLTQDLADLAQADAQQLKCYLSSVNPWEVILQEVENFRPKLEQAGLEIEVEGEGASLLLDRDRFKQIIVNLIGNSIRYTETGGKIHIHTSQTPQEWTLY
29 20Y0020   93.8%  99.4%
GIRKATPEHLDAMMRQVSSLKKLTQDLADLAQADAQQLKCYLSSVNPWEVILQEVENFRPKLEQAGLEIEVEGEGATLLLDRDRFKQIIVNLIGNSIRYTETGGKIHIHTSQTPQEWTLY
30 20Y0036   93.8%  99.4%
GIRKATPEHLDAMMRQVSSLKKLTQDLADLAQADAQQLKCYLSSVNPWEVILQEVENFRPKLEQAGLEIEVEGEGATLLLDRDRFKQIIVNLIGNSIRYTETGGKIHIHTSQTPQEWTLY
31 20Y0083   93.8%  99.6%
GIRKATPEHLDAMMRQVSSLKKLTQDLADLAQADAQQLKCYLSSVNPWEVILQEVENFRPKLEQAGLEIEVEGEGASLLLDRDRFKQIIVNLIGNSIRYTETGGKIHIHTNQTPQEWTLY
32 20Y0076   93.8%  99.6%
GIRKATPEHLDAMMRQVSSLKKLTQDLADLAQADAQQLKCYLSSVNPWEVILQEVENFRPKLEQAGLEIEVEGEGASLLLDRDRFKQIIVNLIGNSIRYTETGGKIHIHTSQTPQEWTLY
33 20Y0078   93.8%  99.6%
GIRKATPEHLDAMMRQVSSLKKLTQDLADLAQADAQQLKCYLSSVNPWEVILQEVENFRPKLEQAGLEIEVEGEGASLLLDRDRFKQIIVNLIGNSIRYTETGGKIHIHTNQTPQEWTLY
34 20Y0043   93.8%  99.4%
GIRKATPEHLDAMMRQVSSLKKLTQDLADLAQADAQQLKCYLSSVNPWEVILQEVENFRPKLEQAGLEIEVEGEGATLLLDRDRFKQIIVNLIGNSIRYTETGGKIHIHTSQTPQEWTLY
35 20Y0048   93.8%  99.4%
GIRKATPEHLDAMMRQVSSLKKLTQDLADLAQADAQQLKCYLSSVNPWEVILQEVENFRPKLEQAGLEIEVEGEGATLLLDRDRFKQIIVNLIGNSIRYTETGGKIHIHTSQTPQEWTLY
36 20Y0026   93.8%  99.4%
GIRKATPEHLDAMMRQVSSLKKLTQDLADLAQADAQQLKCYLSSVNPWEVILQEVENFRPKLEQAGLEIEVEGEGATLLLDRDRFKQIIVNLIGNSIRYTETGGKIHIHTSQTPQEWTLY
37 20Y0030   93.8%  99.4%
GIRKATPEHLDAMMRQVSSLKKLTQDLADLAQADAQQLKCYLSSVNPWEVILQEVENFRPKLEQAGLEIEVEGEGATLLLDRDRFKQIIVNLIGNSIRYTETGGKIHIHTSQTPQEWTLY
38 20Y0082   93.8%  99.6%
GIRKATPEHLDAMMRQVSSLKKLTQDLADLAQADAQQLKCYLSSVNPWEVILQEVENFRPKLEQAGLEIEVEGEGASLLLDRDRFKQIIVNLIGNSIRYTETGGKIHIHTSQTPQEWTLY
39 20Y0024   93.8%  99.4%
GIRKATPEHLDAMMRQVSSLKKLTQDLADLAQADAQQLKCYLSSVNPWEVILQEVENFRPKLEQAGLEIEVEGEGATLLLDRDRFKQIIVNLIGNSIRYTETGGKIHIHTSQTPQEWTLY
40 20Y0038   93.8%  99.4%
GIRKATPEHLDAMMRQVSSLKKLTQDLADLAQADAQQLKCYLSSVNPWEVILQEVENFRPKLEQAGLEIEVEGEGATLLLDRDRFKQIIVNLIGNSIRYTETGGKIHIHTSQTPQEWTLY
41 20Y0059   93.8%  99.4%
GIRKATPEHLDAMMRQVSSLKKLTQDLADLAQADAQQLKCYLSSVNPWEVILQEVENFRPKLEQAGLEIEVEGEGATLLLDRDRFKQIIVNLIGNSIRYTETGGKIHIHTSQTPQEWTLY
42 20Y0067   93.8%  99.4%
GIRKATPEHLDAMMRQVSSLKKLTQDLADLAQADAQQLKCYLSSVNPWEVILQEVENFRPKLEQAGLEIEVEGEGATLLLDRDRFKQIIVNLIGNSIRYTETGGKIHIHTSQTPQEWTLY
43 20Y0055   93.8%  99.4%
GIRKATPEHLDAMMRQVSSLKKLTQDLADLAQADAQQLKCYLSSVNPWEVILQEVENFRPKLEQAGLEIEVEGEGATLLLDRDRFKQIIVNLIGNSIRYTETGGKIHIHTSQTPQEWTLY
44 20Y0077   98.4%  99.4%
GIRKATPEHLDAMMRQVSSLKKLTQDLADLAQADAQQLKCYLSSVNPWEVILQEVENFRPKLEQAGLEIEVEGEGASLLLDRDRFKQIIVNLIGNSIRYTETSGKIHIHTNQTPQEWTLY
45 20Y0058   93.8%  99.6%
GIRKATPEHLDAMMRQVSSLKKLTQDLADLAQADAQQLKCYLSSVNPWEVILQEVENFRPKLEQAGLEIEVEGEGASLLLDRDRFKQIIVNLIGNSIRYTETGGKIHIHTSQTPQEWTLY
46 20Y0062   93.8%  99.4%
GIRKATPEHLDAMMRQVSSLKKLTQDLADLAQADAQQLKCYLSSVNPWEVILQEVENFRPKLEQAGLEIEVEGEGATLLLDRDRFKQIIVNLIGNSIRYTETGGKIHIHTSQTPQEWTLY
```

47 20Y0033   93.8% 99.4%

GIRKATPEHLDAMMRQVSSLKKLTQDLADLAQADAQQLKCYLSSVNPWEVILQEVENFRPKLEQAGLEIEVEGEGATLLLDRDRFKQIIVNLIGNSIRYTETGGKIHIHTSQTPQEWTLY

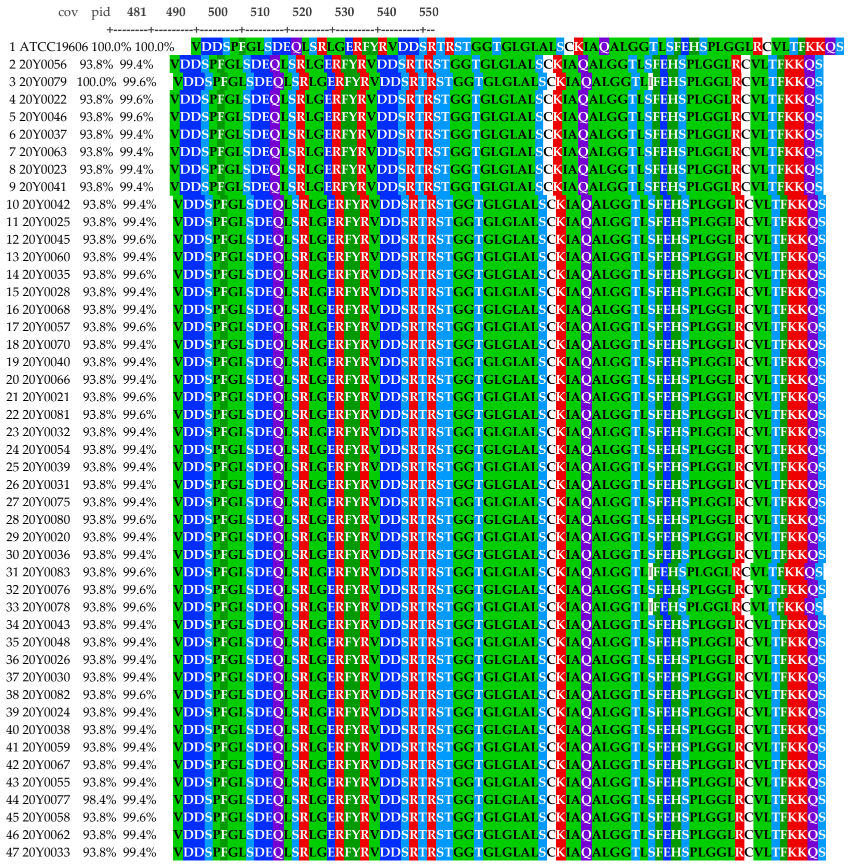

Figure S7: Multiple sequence alignment of the predicted amino acid sequences of BaeS in all isolates compared to that of *A. baumannii* ATCC 19606. The alignment was visualized using MView version 1.63. cov, coverage; pid, percent identity.

Table S4: Summary of mutations affecting *adeRS* and *baeRS* and correlation to tigecycline resistance

Gene	Codon	Wildtype	Mutation	No. of Affected Strains	GC	TG^R
adeR	24	Ile	Met	1	7	100.0%
	56	Pro	Ser	1	7	100.0%
	120	Val	Ile	41	All except 4 and 9	75.6%
	136	Ala	Val	31	2	70.9%
	137	Thr	Ala	1	7	100.0%
	142	Leu	Ile	3	4	66.7%
	150	Thr	Asn	9	5	88.9%
	158	His	Leu	9	5	88.9%
	195	His	Gln	9	5	88.9%
	228	Ile	Val	1	7	100.0%
	241	Pro	Leu	46	All	74.0%
	243	Val	Ile	4	4 and 7	75.0%
AdeS	153	Ala	Thr	46	All	74.0%
	186	Gly	Val	15	All except 2	80.0%
	197	Leu	Phe	4	Subset of GC5	75.0%
	214	Leu	Phe	44	All except 9	75.0%
	227	Asp	His	10	5 and 7	90.0%
	263	Ser	Ala	46	All	74.0%
	268	Asn	His	26	Subset of GC2, GC9 and GC7	61.5%
			Tyr	8	Subset of GC2	100.0%*
	280	Ala	Ser	46	All	74.0%
	281	Asp	Gln	46	All	74.0%
	312	Ser	Ala	3	4	66.7%
	318	Gly	Ser	1	One isolate of GC9 (20Y0080)	0.0%
	331	Val	Ile	44	All except GC9	72.7%
	332	Val	Met	2	9	50.0%
	337	Thr	Ser	1	7	100.0%
	346	Lys	Arg	1	7	100.0%
	348	Val	Ile	32	2 and 7	71.8%
	353	Ile	Thr	1	7	100.0%
	354	Ser	Pro	15	All except GC2	80.0%
	356	Asn	Asp	1	7	100.0%
	357	Asn	Asp	1	7	100.0%
baeR	223	Phe	Leu	1	7	100.0%
baeS	10	Leu	Met	1	7	100.0%
	230	Arg	Gln	46	All	74.0%
	436	Ala	Thr	1	One GC9 isolate (20Y0057)	100.0%*
	437	Ser	Thr	31	2	70.9%
	463	Gly	Ser	1	7	100.0%
	471	Asn	Ser	41	5, 2 and one GC9 isolate (20Y0080)	75.6%
	533	Ser	Ile	3	4	66.7%

*Mutations were only found in TG-resistant but not in TG-sensitive strains of the same GC/ST.
